# Molecular Architecture of Spinal Cord Injury Protein Interaction Network

**DOI:** 10.1371/journal.pone.0135024

**Published:** 2015-08-04

**Authors:** Ali Alawieh, Mohammed Sabra, Zahraa Sabra, Stephen Tomlinson, Fadi A. Zaraket

**Affiliations:** 1 Neuroscience Institute, Department of Neurosciences, Medical University of South Carolina, Charleston, SC 29425, United States of America; 2 Department of Electrical and Computer Engineering, American University of Beirut, Beirut, Lebanon; 3 Department of Microbiology and Immunology, Children’s Research Institute, Medical University of South Carolina, Charleston, SC 29425, United States of America; 4 Ralph H. Johnson Veteran Affairs Medical Center, Charleston, SC 29425, United States of America; Rutgers-Robert Wood Johnson Medical School, UNITED STATES

## Abstract

Spinal cord injury (SCI) is associated with complex pathophysiological processes that follow the primary traumatic event and determine the extent of secondary damage and functional recovery. Numerous reports have used global and hypothesis-driven approaches to identify protein changes that contribute to the overall pathology of SCI in an effort to identify potential therapeutic interventions. In this study, we use a semi-automatic annotation approach to detect terms referring to genes or proteins dysregulated in the SCI literature and develop a curated SCI interactome. Network analysis of the SCI interactome revealed the presence of a rich-club organization corresponding to a “powerhouse” of highly interacting hub-proteins. Studying the modular organization of the network have shown that rich-club proteins cluster into modules that are specifically enriched for biological processes that fall under the categories of cell death, inflammation, injury recognition and systems development. Pathway analysis of the interactome and the rich-club revealed high similarity indicating the role of the rich-club proteins as hubs of the most prominent pathways in disease pathophysiology and illustrating the centrality of pro-and anti-survival signal competition in the pathology of SCI. In addition, evaluation of centrality measures of single nodes within the rich-club have revealed that neuronal growth factor (NGF), caspase 3, and H-Ras are the most central nodes and potentially an interesting targets for therapy. Our integrative approach uncovers the molecular architecture of SCI interactome, and provide an essential resource for evaluating significant therapeutic candidates.

## Introduction

Spinal cord injury (SCI) is a prominent cause of disability worldwide, and in the U.S. is second only to stroke as a cause of disability, accounting for 23% of all cases of paralysis [1]. Following mechanical injury to the spinal cord, a post-traumatic inflammatory response occurs which is thought to play an important role in secondary neuronal injury and impairment of functional recovery. While nothing can be done for the initial trauma, the subsequent inflammatory response represents a therapeutic target. With the optimization of management protocols, the survival rate of SCI has increased, but around 60% of SCI patients still develop paraplegia [2]. The fact that there is no effective therapeutic intervention for the treatment of SCI highlights the need for a better and more integrative understanding of the molecular mechanisms that promote pathology and determine recovery.

The physiological sequence of events that occur after SCI include spinal shock, autonomic disturbances and spasticity, and gross pathological changes [3]. Individual contributing mechanisms to disease progression after SCI include excitotoxicity, astrogliosis, and inflammation [4–6], but there is limited information available on how pathways involved in these processes connect with each other to result in the pathological outcome. With the aim of providing insight into the interdependency of prominent pathophysiological processes and the molecular disturbances that affect neuronal survival and axonal regeneration, we investigated the full molecular architecture of SCI.

In this work, we use a semi-automatic annotation approach to extract information from the scientific literature on protein and gene disturbances after SCI. We use systems biology tools and resources to map interactions among proteins reported to be dysregulated after SCI, and we use network analysis and graph theoretical algorithms to study the network of protein interactions after SCI. We identify hub proteins, critical pathways, and modules involved in SCI pathogenesis, as well as potential therapeutic targets.

## Materials and Methods

### Extraction of Target Dataset

Literature on spinal cord injury was extracted from PubMed using the MeSH term (Spinal Cord Injuries) and the search key ("Traumatic Spinal Cord"[Title/Abstract] OR "Spinal Cord Trauma"[Title/Abstract]]), as well as from references of reviews and extracted papers. We have used this broad search key to ensure the highest sensitivity in detecting relevant literature while using manual filtering to warrant specificity. Two scientific curators (AA and ZS) with experience in neurosciences and systems biology screened the extracted papers using titles and abstracts to exclude reports that do not study protein or gene involvement in SCI pathogenesis. Examples of excluded papers include those reporting imaging findings after SCI, describing clinical diagnostic and management approaches for SCI patients, studying the epidemiology of SCI, or studying a different disease than SCI. Therefore, only papers that report specific genes or proteins to be involved in SCI pathophysiology were including regardless of the studied species. The reason for pooling data from studies in multiple species is to curate all protein/gene changes that are involved in SCI and determine whether these genes/proteins form a distinctive network topology that may reveal central and major contributor to the overall pathology. An in-house annotator tool that visualizes the abstracts and allows the authors to decide rapidly on whether to include or exclude an abstract was used to speed the selection process. To ensure agreement between the two curators, inter-annotator agreement analysis was performed, variations across the two curators were detected, and final decisions were discussed among authors. Selected papers were then processed into an annotator tool (designed by authors) for extraction of protein and gene terms reported in association with SCI.

### Text Annotation and Accession Mapping Tools

**Fig 1:**
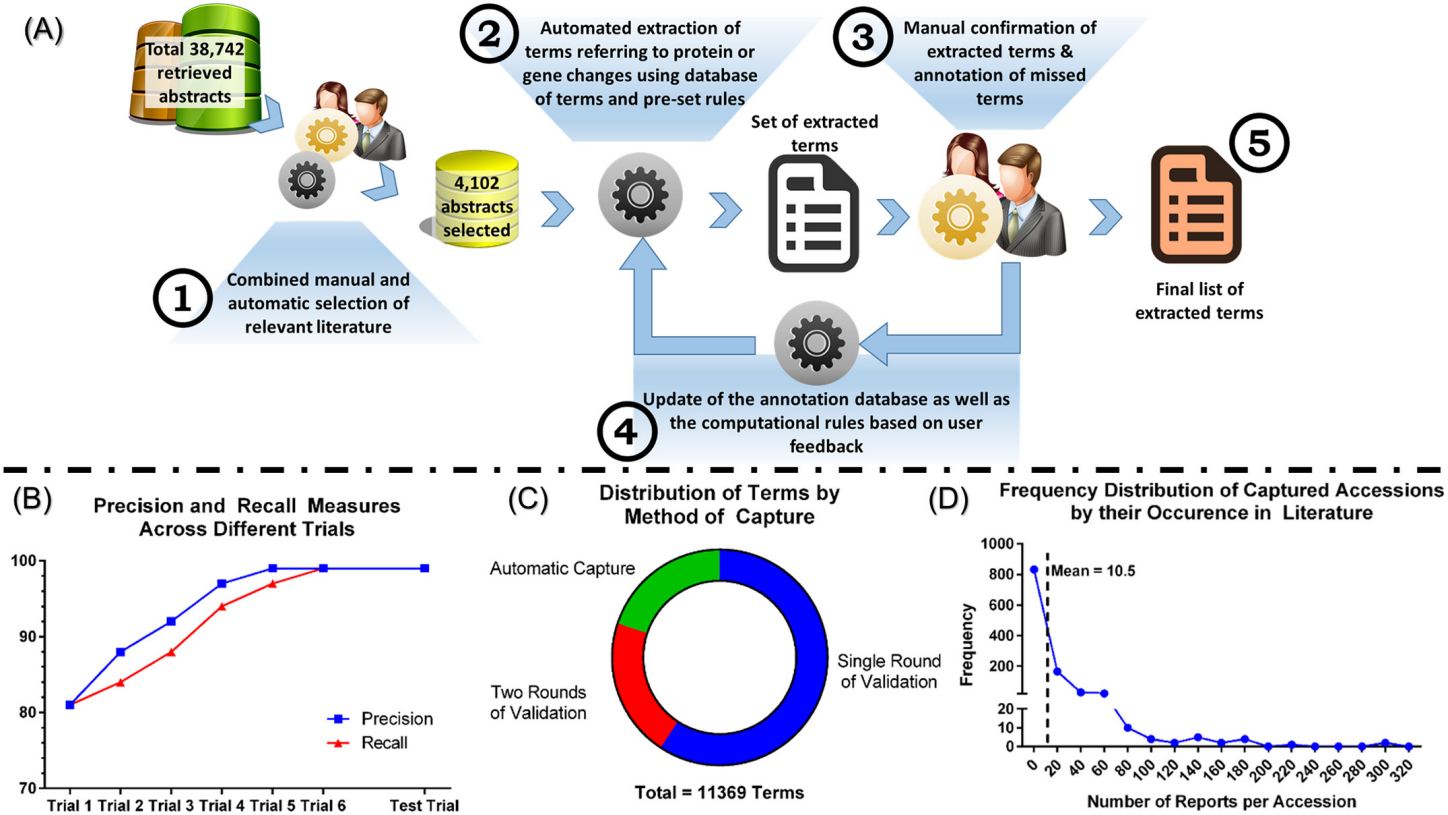
Design and output of the curation protocol. (A) Overall Scheme of the Annotation and Term Detection Process. After articles are selected from PubMed using the search key, (1) a simultaneous manual and automatic selection of abstracts of papers referring to protein/gene changes after SCI to ensure best sensitivity and specificity of capture. Manual selection was performed by human annotators while automatic selection was performed through detection of desired terms in abstract (protein names, terms indicating changes in regulation, etc…). (2) We used an in-house tool that reads protein and genes names from online ontologies and then automatically detect exact or similar matches in the abstracts using pre-set rules. Then, (3) human annotators confirmed the extracted terms, delete false positives and (4) updated the tool with missed terms or rules for future use. Finally, (5) a full list of extracted terms is reported and referred to as the SCI meta-proteome (S3 Table). (B) Precision and recall results at different trials of annotation. Six trials of manual cross-validation and optimization were required to achieve more than 99.5% precision and recall of the tool (see methods for details). Test trial was performed to confirm the results. (C) Distribution of terms by method of capture. Around 60% of captured terms were validated by a single round of manual validation (by two human annotators), another 20% were validated by two rounds of manual validation due to low frequency or incomplete protein name description, and remaining 20% were automatically captured. (D) Frequency distribution of captured accessions by their literature occurrence.

To extract protein and gene names and identifiers from the text, we adopted a semi-automatic annotation protocol using an in-house annotator to ensure specificity and sensitivity of capture. The annotator: 1. Extracts different versions of gene and protein names and abbreviations from UniProt (http://www.uniprot.org) and HGNC (http://www.genenames.org) databases, 2. Checks the text of the extracted abstracts with the gene and protein terms extracted from the databases to annotate exact matches and, 3. Detects and annotates close matches based on variations of the extracted terms. Variations were defined in the form of computational rules to detect differences in spelling, the mentioning of specific and multiple subunits, and variability in the use of special characters and abbreviations (S1 Table). The detected and annotated terms were extracted into separate datasets, each identified by the ID of the source paper. A human annotator with experience in both neurotrauma and proteomics verified the captured terms and inspected the abstracts using the same annotator tool for additional terms. The annotators ran several trials of manual annotation to assess the precision and recall of the tool. Each trial included around 1000 annotated terms with their corresponding abstracts. After each trial, the set of rules was updated based on annotators’ input. Trials were performed with rules updates until the tool achieved more than 99.5% on precision and recall measures. Fig 1a summarizes the overall approach used for term extraction. Next, an in-house C# program communicated with UniProt and HGNC to retrieve the corresponding accessions of captured terms. The frequency of each accession was computed as the number of distinct reports of accession in question. To avoid being biased by the literature, there was no assignment of weights for frequency in our network analysis, but terms with a frequency less than three were cross-validated by a second annotator to ensure minimal false positive captures (Fig 1c).

### Functional Annotation and Interactome Data

We used the Database for Annotation, Visualization and Integrated Discovery (DAVID) [7,8] to annotate the retrieved list of accession for GO (Gene Ontology) cellular component, GO biological processes, and KEGG (Kyoto Encyclopaedia of Genes and Genomes) pathways. Functional annotation clustering was also performed through DAVID to identify enrichment scores for different groups of annotations. For clarity of presentation, we report p-values of corrected Fischer exact t-test for enrichment analyses as enrichment scores which are computed as log10(1/p-value). Protein-protein interactions (PPI) were obtained from STRING databases by including interactions among the list of accessions with a combined score higher than 0.4 [9,10]. S2 Table summarizes the different tools and resources used in this paper.

### Graph Theory Measures

#### General Network Properties

After construction of the SCI interactome, graph theory was used to examine the topology of the network as previously described [11]. Calculated graph metrics include: node-specific degree (k), clustering coefficient, average path length, and network efficiency. These measures, in addition to power-law distribution metric, were computed using the Systems Biology and Evolution (SBE) Toolbox in MATLAB [12]. To confirm the presence of small-world organization in the network, the graph’s average path length and clustering coefficient was compared to that of 1000 randomly generated and comparable networks, and the small-world index was calculated as previously described [13].

#### Centrality Measures

In addition to degree centrality (k), other centrality metrics were computed for the different nodes in the network, including closeness centrality and betweenness centrality. Brokering coefficients were also calculated for each node as previously described [14].

#### Rich-Club Detection

Investigation and analysis of rich-club organization within the SCI interactome was a major aim of this paper. A rich-club organization is present in a network when high degree nodes are heavily interconnected among each other, and more so than predicted by chance. A rich-club coefficient for a certain degree k, referred to as φ(k), is defined as the ratio of connections present between nodes with degree > k (E>k) to the total number of possible interactions between those nodes [11,15,16]. This is given by: φ(k) = 2* E>k / (Nk(Nk-1); where N>k is the total number of nodes with degree above k. However, because random networks may also have high interconnections among high degree nodes, rich-club coefficients, φ(k) are usually normalized to a set of comparable random networks with equal size and similar degree distribution; a normalized rich-club coefficient, ρ(k), is defined as the ratio of φ(k) of the network to φrandom(k), which is the average rich-club coefficient of a set of comparable randomly generated networks. In this report, our φ(k) was compared to that of 1000 randomly generated networks for calculating normalized ρ(k). To assess statistical significance of rich-club organization, we computed the distribution of φrandom(k) for the 1000 generated random networks and used a one-sample t-test to assess whether φ(k) significantly exceeded φrandom(k) over the range of the rich-club.

#### Knotty centrality

A knotty centre in a network is a subgraph that acts as a topologically central connective core. We used the heuristic algorithm developed by Shanahan et al [17] to find the subgraph in our network and in the network of the rich-club having the highest knotty centrality.

#### Module organization

To study modular organization of the full network, as well as the network of the rich-club, the previously described MCODE algorithm was used [18]. Further, after nodes were clustered into modules, functional annotation for the most enriched GO biological processes in each module was performed.

### Network Visualization and Statistical Analyses

Network visualization was performed in Cytoscape 3.1.1 (Cytoscape Consortium) [19]. Statistical analysis was performed through Graphpad Prism 6 (GraphPad Software, Inc.). One-sample t-test was used to compare network metrics to those of comparable randomly generated networks. Standard student t-test was used to compare literature frequency of rich-club vs. non-rich club nodes. Modified Fischer exact t-test was used for enrichment analyses [8].

## Results

### Evaluation and validation of term capture

We retrieved 38,742 abstracts from PubMed searches of which 4,102 abstracts were found to discuss relevant information on protein changes in the context of SCI. After six trials of manual cross-validation and tool optimization (Fig 1a), we were able to achieve more than 99.5% on both precision and recall measures, which was further confirmed by a test trial (Fig 1b). We then captured a total of 11,369 terms that included automatically captured and manually verified terms (Fig 1c). Captured terms were mapped to 1,083 unique accessions for proteins or genes associated with SCI pathogenesis. The distribution of those accessions by occurrence in the literature was right skewed with a mean of 10.5 reports per accession (Fig 1d, See Methods). The full list of curated accessions along with their frequencies and references are shown in S3 Table. We named this list an SCI meta-proteome, defined as the protein complement of SCI changes curated from experiments in different animal species.

### General description of SCI meta-proteome and interactome

Accessions with the highest frequency in the literature were those of GFAP (Glial Fibrillary Acidic Protein), followed by BDNF (Brain Derived Neurotrophic Factor) and iNOS (inducible Nitric Oxide Synthase). However, frequency of encounter of these accessions only reflects the interest of the scientific community and may not be an accurate measure of the contribution of different proteins to the overall pathogenesis of SCI. Therefore, using network analysis of the interacting proteins in SCI interactome (SCII), we performed an assessment of the relative contribution of each of the curated proteins to disease pathogenesis using objective and quantitative measures. To do this, we constructed an SCI interactome from published PPI information, including interactions among the curated 1,083 protein accessions. The resulting SCII includes 22,341 interactions among 1,050 connected nodes (S1 Fig). We then used the SCI meta-proteome data for functional annotation and term enrichment analysis, while we used the SCII for analysis of network topology, modularity and rich-club organization.

**Fig 2:**
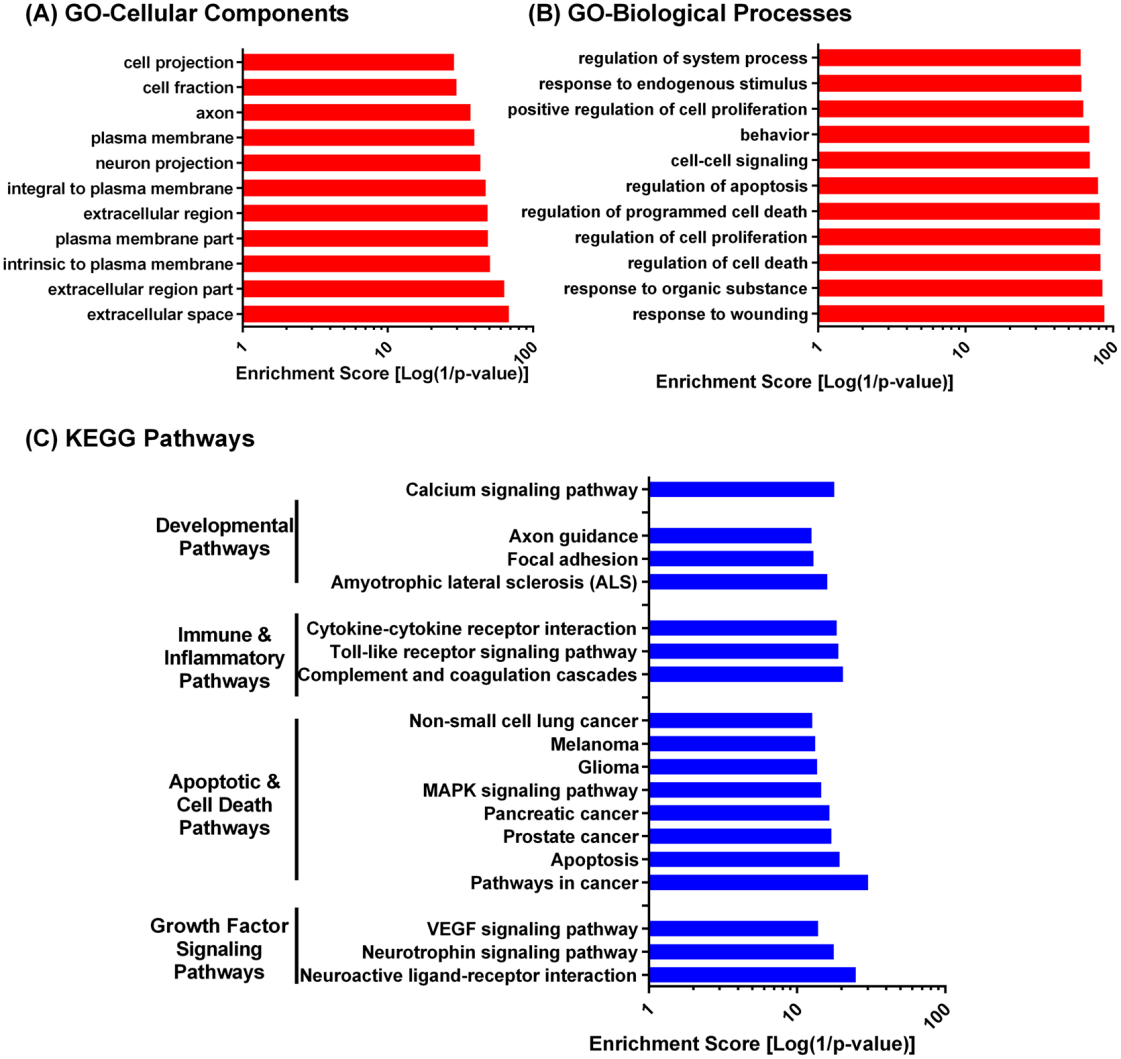
Functional annotation of the SCI meta-proteome. Enriched GO cellular components (A), GO biological processes (B), and KEGG pathways (C). Terms restricted to most significantly enriched terms (p-value <1E-10). For clarity of presentation, enrichment scores were reported as log (1/p-value). Full list of DAVID enrichment analysis is available in S4, S5 and S6 Tables.

Functional annotation of the SCI meta-proteome was performed to provide a global overview of the pathophysiological processes and pathways over-represented in the SCI meta-proteome (Fig 2). Gene Ontology analysis [7,8] showed significant overrepresentation of proteins located in the extracellular, plasma membrane and neuronal compartments (Fig 2a), as well as those involved in response to wounding and organic stimulus, cellular death and proliferation, and cell-cell signaling (Fig 2b). We also analyzed biological and disease pathway enrichment using the KEGG database. The top enriched pathways fell into four major categories: 1. Growth factor signaling pathways, 2. Apoptotic and cell death pathways, 3. Inflammatory and immune pathways and, 4. Developmental pathways and calcium signaling pathway (Fig 2c). These findings fit with our current understanding of SCI injury, hallmarks of which include apoptosis and cell death, immune system activation and calcium signaling [4,20].

### Rich-club organization within the SCII network topology

**Fig 3:**
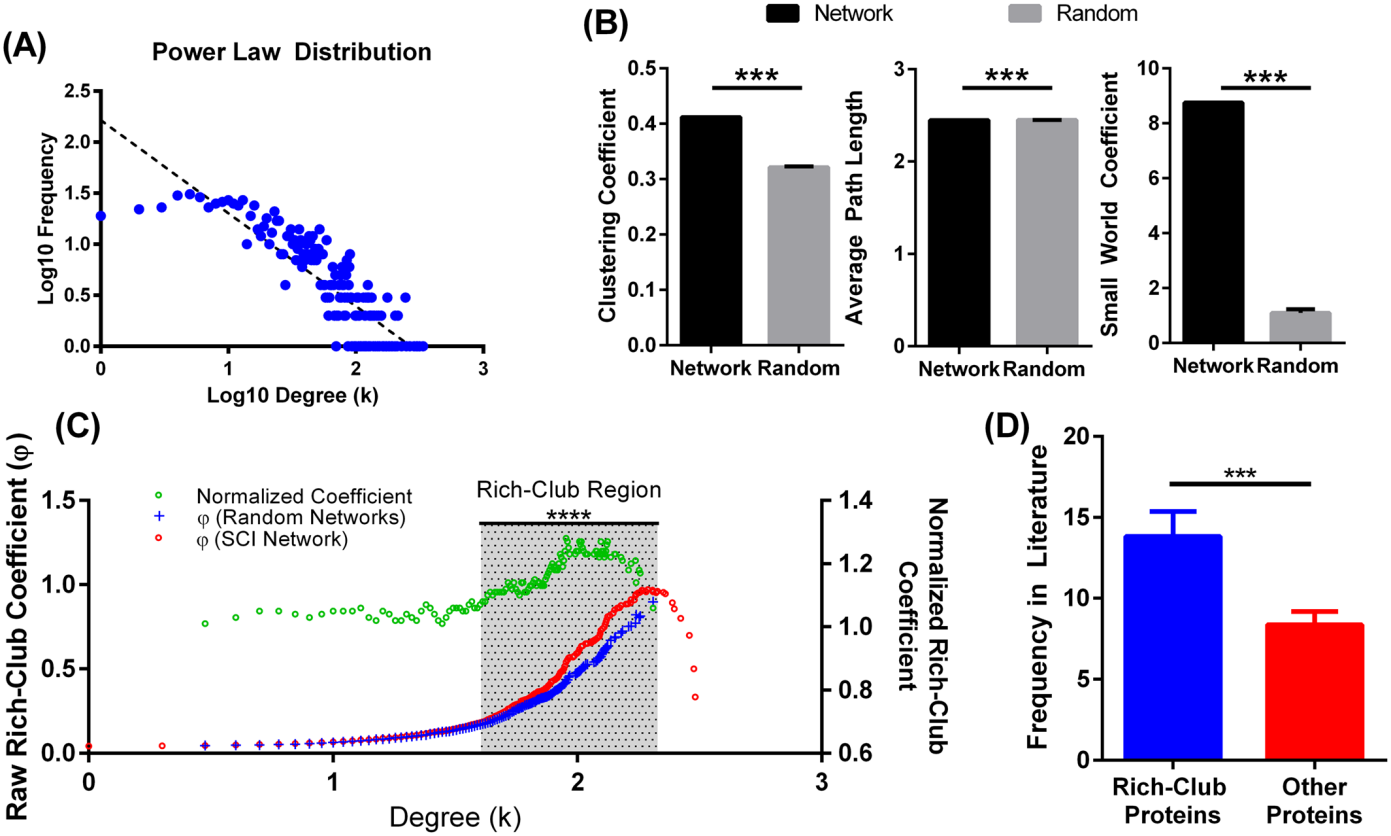
Network properties of the SCI interactome. (A) Power-Law distribution of nodes in the SCII showing a pattern typical of a scale free network: High frequency of non-hub nodes (nodes with few connections) and low frequency of hubs (nodes with high connections). (B) Detection of rich-club organization in the SCII. As described in methods, rich-club coefficient (φ(k)) was computed across the range of degrees in the network and compared to the rich-club coefficient (φ¬random(k)) of 1000 comparable random networks. Raw rich-club coefficient measures mapped to right vertical axis. Normalized coefficient mapped to the left vertical axis. A normalized coefficient > 1 indicates an RC organization. One sample t-test, ****average p-value = 2 E-12. (D) Frequency in literature of rich-club vs. non-rich-club proteins. Mean +/- SEM. Student t-test. *** p<0.0001.

Similar to other biological networks, we found that the SCI interactome exhibited both scale-free and small-word properties. Degree distribution of SCII nodes show a power-law distribution consisting a high frequency of proteins with low connections, and very few genes with high connections, i.e. hubs [21] (Fig 3a). General features of the network are also compatible with small-world organization with a relatively low path length (s = 2.45) and high clustering coefficient (t = 0.42), yielding a significantly higher small-world coefficient compared to random networks [22] (Fig 3b). Since scale-free networks can exhibit special topological orders, such as knotty centres (a connective core within the network [17]) or a rich-club (a dense core of highly interconnected nodes [16]), we assayed for the presence of such organizations in the SCII. A principal finding of this paper is the identification and characterization of a rich-club organization in the SCII. (Fig 3c) shows the normalized rich-club coefficient (ρ(k)) of the SCII network over the range of node-specific degrees (k) along with the raw rich-club coefficient (φ(k)) and random rich club coefficient (φrandom(k)), derived from 1000 randomly generated networks. The shaded region indicates the region of the rich-club covering the p(k) range 36–203. For statistical confirmation, a one-sample t-test was carried out over the range of the rich-club; comparing network coefficients to that of random networks revealed a high significance (average p-value across rich-club range = 2xE-12). In the context of disease related protein interactome, the characterization of the rich-club provides avenues for the exploration of pathways and proteins that form the powerhouse of the overall network of pathophysiology.

Although the frequency of occurrence of the extracted terms in the literature was not incorporated in network analysis (see Methods), comparison of frequency of occurrence of rich-club and non-rich-club proteins show that rich-club proteins were twice more frequently studied in the literature (Fig 3d). This indicates that researches have targeted the set of rich-club proteins, potentially due to their biological impact prior to the characterization of the rich-club, a finding that supports the significance of our characterization of the SCI rich-club.

### Functional and modular dissection of the SCII rich-club

**Fig 4:**
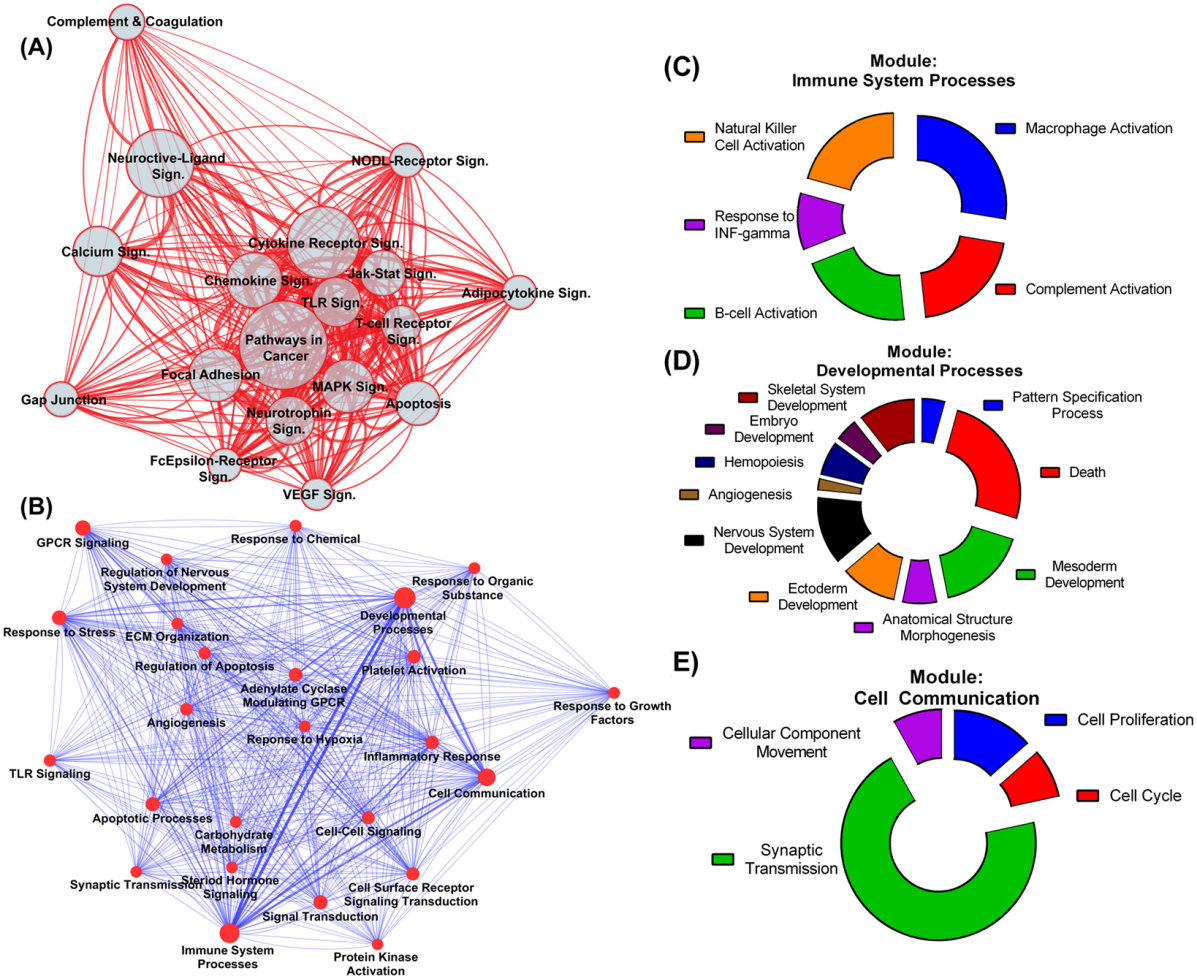
Interaction networks of rich-club pathways and modules. (A) Network of interacting pathways among rich-club nodes. Visualized pathways are those with an over-representation p-value <1E-10. Node size (circles) are mapped to the number of nodes within each pathway while the edge width is mapped to the number of interactions among components of the corresponding pathways. The network is shown as a weighted layout with pathways densely interconnected are clustered together. Enrichment statistics are shown in S9 Table. Sign = Signaling. (B) Rich-club modules enriched for specific GO biological processes. Network style and layout is similar to (A). S1 File allows for exploring the different subnetworks for each module in addition to the enrichment statistics and the number of genes within each pathway. Weighted node-specific degrees for the different modules is available in S9 Table. (C-E) Detailed gene distribution within the most over-represented GO biological processes for select modules with highest weighted node-specific degrees; immune system processes (C), developmental processes (D) and cell communication (E).

We assessed whether the rich-club subnetwork had a different set of enriched KEGG pathways compared to the full network. The most significantly enriched pathways in the rich-club subnetwork (p-value <1E-10) are shown in Fig 4a and were found to have significant overlap with those enriched in the full network (100% overlap in top 10 pathways), clearly indicating that the rich-club involves the major hubs of the key pathways in the network. We further studied the interaction among the prominent rich-club pathways, and used weighted topological visualization to examine the inter-relations among those pathways. We found dense interconnections between the different rich-club pathways centered on a densely connected core of pathways involved in the final death or survival decision in the cell (Fig 4a). Peripheral pathways involve those responsible for recognition of micro-environmental changes, including injury detection (complement and coagulation cascade, NOD-like receptor signaling, Fc-epsilon receptor signaling), growth factor recognition (VEGF and neuroactive ligand signaling), and response to extracellular changes (calcium signaling, gap junctions). These peripheral pathways are less densely interconnected and serve as relays of micro-environment information to the central core of signal transduction pathways to favour an ultimate outcome of neuronal degradation or survival. Further breakdown of the central core into specific pathways using Panther DB illustrates the specific components of cell death pathways, cell communication, growth factor signaling and immune cell signaling that are major determinants of the output of the central core (S8 Table).

In addition to pathway analysis, analysis of network modularity, as well as assessment of biological process over-representation within modules, may reveal clusters that are both topologically and functionally distinct. We used the MCODE algorithm and identified 28 different modules, 25 of which had more than three nodes and were functionally annotated for enriched biological processes. Indeed, each of the 25 different modules was enriched for a specific GO-biological process, indicating that the modules are both topologically and functionally distinct. Fig 4b shows a hierarchical weighted layout of the different rich-club modules labelled by their corresponding enriched GO processes. S1 File allows for inspection of individual subnetworks of the different modules. Such modular organizations provide insight into the contributing communities within the rich-club, and allow for a more detailed analysis of pathogenic mechanisms. Within the network of rich-club modules, the three top degree modules were those of developmental processes (including nervous and skeletal system developments and angiogenesis), immune system processes (including complement and immune cell activation) and cell communication (including synaptic transmission) (Fig 4c–4e). Interestingly, cellular signaling modules are the most frequent modules (44%) in the rich-club, matching the findings obtained through pathway enrichment. In addition, Fig 4b shows other less prominent modules/processes that are also involved in responses after SCI, such as platelet activation, angiogenesis, response to hypoxia, and extracellular matrix organization.

### Knotty Centrality assessment

**Fig 5:**
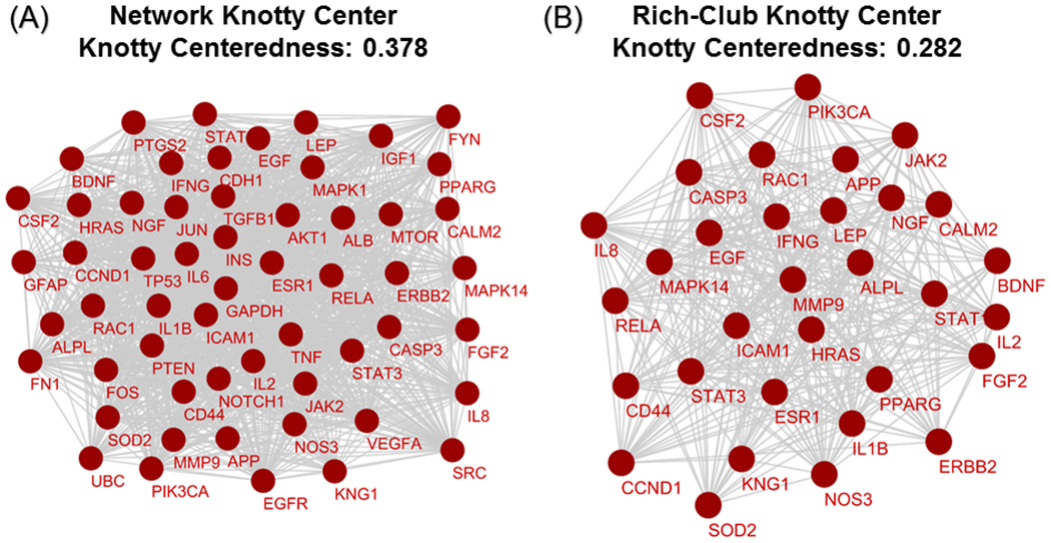
Knotty centre sub-networks. (A) Interactions among components of network’s knotty centre. (B) Interactions among components of rich-club’s knotty centre.

We assessed whether the SCII exhibits a significant knotty centre in addition to the rich-club organization, which acts as the network’s connectivity core. Compared to rich-club coefficients, knotty centrality is not based on the node-specific degree measures and would thus provide distinct information from that provided by the rich-club [17]. Although the rich-club captures the dense interconnected core of the network, it provides little information on the relation of this core to other non-rich-club components of the network, a relation that can be explored through knotty centrality measures. We performed knotty centrality analysis on both the entire network and on the rich-club, and report one subnetwork of each that has the highest knotty centeredness. Fig 5a and 5b show the most significant knotty centre subnetwork of the entire SCII and of the rich-club, respectively. We found a significant overlap between both the network knotty centre and the rich-club knotty centre (common nodes are ~ 67% of total nodes of each subnetwork). This indicates that in addition to its role as a powerhouse of SCI pathophysiology, the SCII rich-club is also central to the connectivity of the entire network through acting as a highway system for communication of non-rich-club nodes. In order to confirm this role, we collapsed the rich-club nodes into their corresponding modules in order to dilute the effect of intra-rich-club connectivity, and studied the collapsed network of non-rich-club nodes and rich-club module interactions for the presence and identity of the knotty centre. Indeed, around 65% of this knotty centre was composed of the rich-club modules, supporting the aforementioned role of the rich-club in linking both hub and non-hub nodes in the network.

### Analysis of individual nodes in the network

**Fig 6:**
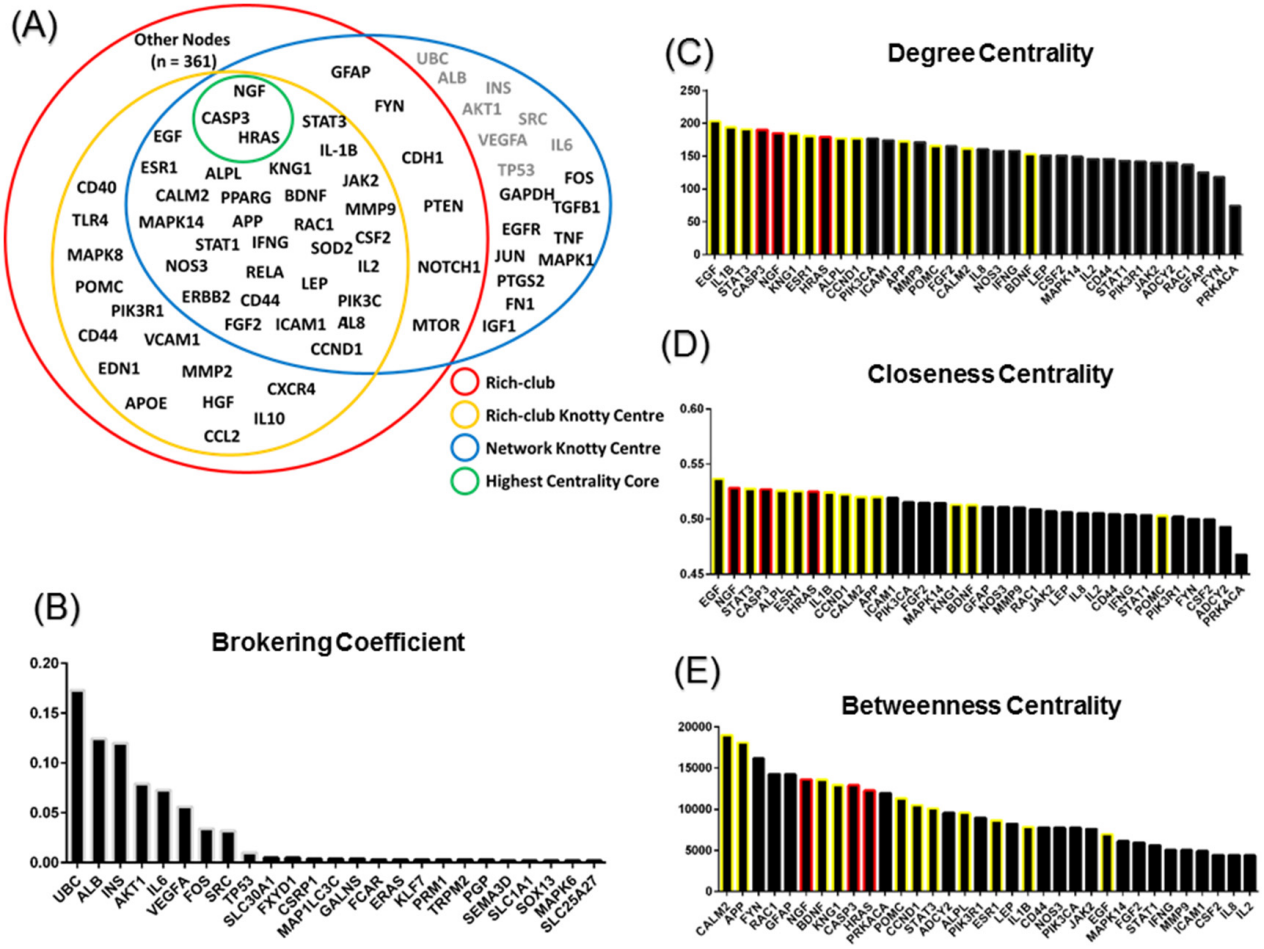
Centrality measures for individual nodes in the SCI meta-proteome. (A) Distribution of protein nodes among the network topological features. Rich-club in red, rich-club knotty centre in gold, network knotty centre in blue and the highest centrality core in green. The highest centrality core includes three nodes that scored more than two standard deviations above average on three different centrality measures. In grey are top brokers in the network. (B) Brokering coefficients for top brokers in the network. Grey border indicates top broker nodes in the network. (C-E) Distribution of nodes by centrality measures. Visualized are nodes having the top 34 scores on three centrality measures (degree centrality, closeness centrality and betweenness centrality. Yellow border indicates nodes that are two standard deviations above average of two different centrality measures. Red border indicates nodes that are two standard deviations above average of all three different centrality measures.

**Fig 7:**
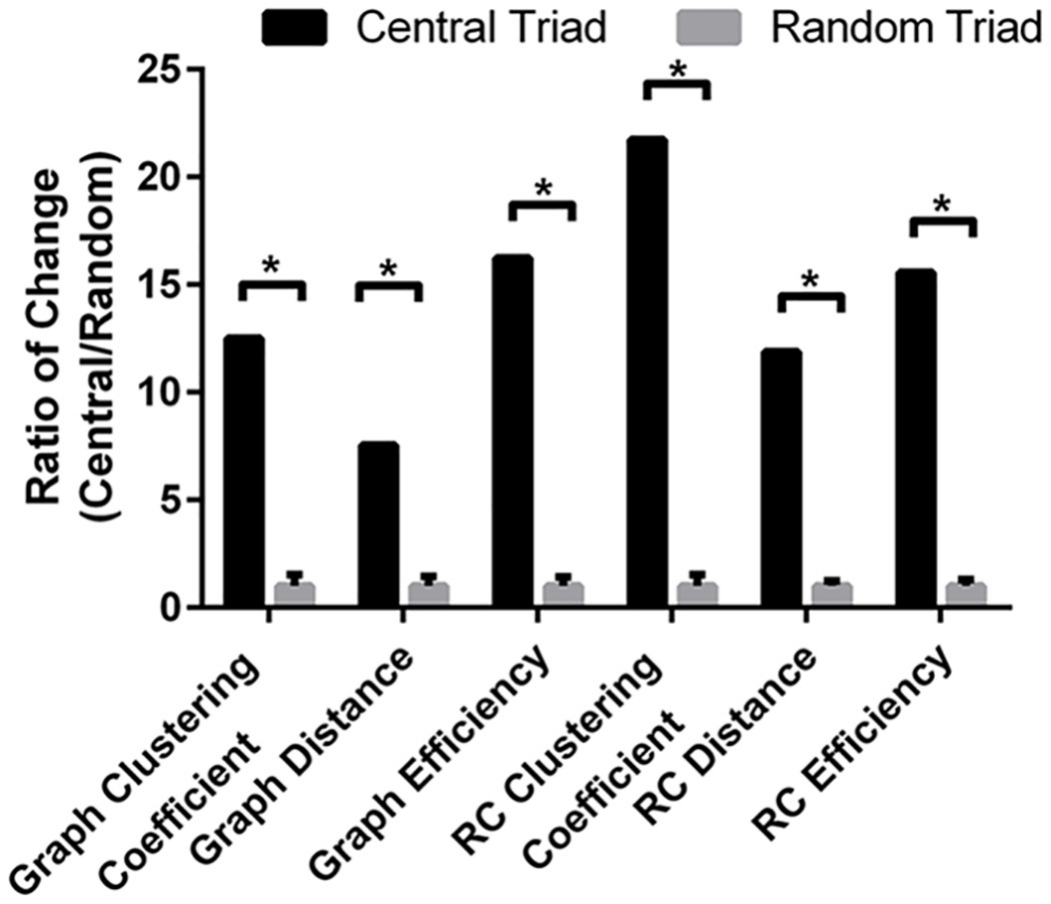
Effect of removing central triad compared to random triad removal on network and rich-club graph measures. Central triad (Neuronal Growth Factor, H-Ras, and Caspase-3). Plotted is the ratio of change caused by central triad removal compared to change induced by random triad removal. Mean +/- SEM. One sample t-test. * p<0.05.

To further study the individual contributions of rich-club and non-rich club nodes, we first examined non-rich club nodes using the collapsed network of non-rich-club nodes and rich-club modules. We found that this network does not exhibit significant modularity; MCODE analysis revealed around 300 modules with less than four nodes each, indicating that non-rich-club nodes include peripheral nodes that contribute to the overall network through interaction with rich-club. However, few non-rich-club nodes still exhibited high centrality measures and were part of the network’s knotty centre (Fig 6a). We therefore hypothesized that these nodes are essential linkers between the rich-club and peripheral nodes in the network. To test this, we calculated the brokering coefficient for the different nodes in the network, and found that most rich-club nodes were not among the high broker genes, whereas the top network brokers were non-rich-club knotty centre genes (Fig 6b). This result confirmed our hypothesis that the role of these genes is connective in nature, and that they link the rich-club nodes to non-rich-club nodes. Fig 6a provides a summary of the nodes that belong to the studied topological organization. In addition, we scored rich-club nodes by their centrality measures, and we highlight three core nodes (NGF, H-Ras, and Caspase-3) of the rich-club that score more than 3 standard deviations above average on all 3 different centrality measures compared to other rich-club nodes (Fig 6c–6e). Interestingly, this central triad (NGF, H-Ras, Caspase-3) reflects the centrality of neurotrophic and neurodegenerative signaling in the overall SCII. To better assess the influence of this triad on the overall network and rich-club, we calculated changes in network and rich-club parameters after removal of this central triad compared to random removal of three rich-club nodes. Removal of the central triad had significantly more impact on measures of network efficiency (Fig 7), further confirming the critical contribution of these nodes to the network and the rich-club.

## Discussion

We used a semi-automatic annotation approach to curate data on proteins and genes implicated in SCI pathogenesis, and we have developed the first curated SCI meta-proteome. We identified 1083 proteins and constructed their corresponding SCI interactome, creating the largest spinal cord injury interactome to date. We also used detailed and complex graph theoretical analyses on the SCI interactome to identify key topological signatures of the interaction network. We report the novel discovery of a rich-club organization within the SCI interactome, and show that it covers the hubs of key pathogenic processes and pathways in SCI. We were also able to study the modularity of the SCI rich-club and report on a distinct organization of topologically and functionally distinct modules within the rich-club. Our analyses revealed that pathways of cell death, cell survival and neurodegeneration form the core of the rich-club, while pathways involved in recognition of injury and response to the environment have more peripheral topology. We also discovered a knotty centre, or a connectivity core, within the network that serves as a major link between the rich-club and peripheral nodes. Finally, we were able to identify a central triad of three nodes (NGF, H-Ras, and Caspase-3) that had a more pronounced effect on the rich-club compared to other nodes.

A key motivation for our work was to obtain an integrative, quantitative and un-biased approach to curate the different findings in molecular SCI and perform secondary analyses. A need for this kind of study is emphasized by the absence of curated SCI gene or protein signatures in databases of gene-disease associations and pathways [23–25]. Our approach integrates findings from the majority of published reports including investigations performed in different species, leading to the first SCI meta-proteome. The term meta-proteome is used to indicate that data originated from separate experiments in different species. Compared to previous literature-based studies that used data from microarray and proteomics databases (Examples [26,27]), a novel feature of our approach is to incorporate data originating from “non-omics” (genomics, transcriptomics or proteomics) approaches, including experiments on single or a limited number of protein changes after SCI. In addition, the fact that curated meta-proteome includes data from multiple species including experimental models of SCI allows both for capturing mediators of injury that cannot be easily probed in human subjects and for identifying proteins whose involvement in SCI has been validated across multiple studies.

In this study, we perform a thorough graph theoretical analysis of the SCI interactome constructed using public PPI datasets. Although previous studies have applied graph theoretical algorithms to study modularity in interactomic datasets [26,28,29], this report is the first to extensively study a disease related interactome using graph theory. We uncover the presence of both a rich-club organization and a knotty centre within the SCI interactome. The presence of a rich-club is highly significant in relation to a disease interactome, as it indicates the presence of a powerhouse of action that groups the most prominent and heavily connected hubs. In the case of complex disease states, such as SCI where thousands of proteins partake in the pathophysiological process and contribute differently to the final outcome, the identification of a rich-club subnetwork within the full SCI network will narrow the pool of therapeutic targets by limiting it to the most influential components. In addition, the fact that the multiple pathophysiological processes over-represented in the SCI interactome were also over-represented within the rich-club, further confirms the pleiotropic nature of the secondary pathophysiological response after SCI and indicates the need for versatile multi-action multi-target therapeutics.

Through the functional annotation of the network by pathways and disease processes using systems biology tools, and through the functional and topological study of rich-club modules, this study highlights core pathogenic mechanisms of SCI. Previous studies have indicated a key role for apoptotic and cell death cascades in the pathogenesis of SCI [30,31], along with a potential role for neuronal growth factors in promoting recovery [32,33]. Studies on animal models of SCI have also identified an early role of changes in extracellular environment after SCI including calcium excitotoxicity [4,34], permeability and disturbances [35,36], vascular abnormalities [37], and oxidative and metabolic stress [38,39]. These changes are also associated with robust activation of immune and inflammatory cascades [40–42]. In addition to supporting the involvement of the discussed mechanisms in SCI, a major contribution of this study to understanding SCI pathophysiology is the assessment of interactions, interdependencies and weighted contributions of these pathways. Network analysis of the modules within the SCI rich-club revealed that pro-and anti-apoptotic mechanisms are at the core of the SCI pathophysiological network, and those mechanisms cluster together in a tight core of modular interactions between cell signaling cascades that hold the final decision of cell death vs. survival. This is also reflected by our finding that the highest centrality triad of NGF, H-Ras and Caspase-3, are prominent components of apoptotic and cell growth cascades, and these proteins had the most significant contribution to the SCI rich-club and interactome. This cell fate decision core represents a major target for SCI therapeutics, especially with current interest in the use of growth factor therapies and stem cell transplantation for treating SCI. We also discovered pathways that do not fall within this core, but were highly over-represented in the rich-club and that at the same time have high centrality measures. Although these pathways fail to form a core and are less heavily interconnected compared to the core pathways, they are comprised of immune system recognition components (complement and coagulation cascade, TLR signaling, NODL receptor signaling), growth factor receptor complexes (neurotrophic growth factors, VEGF), and calcium signaling cascade. A common feature among these elements is that they all include cellular responses to extracellular changes, including recognition of stress antigens by immune cells, recognition of ion disturbances (i.e. calcium), and recognition of growth factor availability. Therefore, the high level of communication between these pathways and the cell survival core, and the minimal interconnection among themselves, point out that these pathway relay different and independent information from the extracellular environment to the decision core to influence cell survival or death. We can describe the role of these pathways as a modulatory effect that can influence the balance of the cell survival decision, and this makes these pathways interesting from a therapeutic perspective. From our data, interventions to reduce immune system activation and recognition of death patterns in the early phases of injury, interventions to improve availability and delivery of growth factors, and interventions to maintain ionic homeostasis would appear to offer better therapeutic approaches compared to interference with cell signaling cascades.

Previous studies involving proteomics and transcriptomics analysis in SCI models have emphasized the contribution of one or some of aforementioned pathways in separate individual studies. For instance, Chen et al studied acute protein dysregulation that occur after SCI in rat using liquid chromatography followed by tandem mass spectrometry and identified a significant involvement of inflammatory, vascular, metabolic and cell trafficking pathways early after SCI [43]. However, studying acute and sub-acute phase of SCI, and Kang et al and Ding et al have respectively identified the significant contribution of neuronal survival and degeneration pathways as well as axon growth pathways in injury and recovery after SCI [44,45]. More relevant to our work is the recent study by Sengupta et al performed proteomic profiling of CSF to identify dysregulated proteins after SCI followed by analysis of modularity among the interaction network of identified proteins. Enrichment analysis of significant pathways among the identified 31 modules revealed the involvement of growth factor signaling, cell adhesion, WNT and p53 signaling, axon guidance, apoptosis and cellular metabolism [46]. Interestingly, many of these pathways were similarly detected in our curated network and identified to be components of the SCI rich-club.

In conclusion, this study establishes the first repository of curated gene and protein changes in SCI. In addition, our work provides a detailed analysis of the topological and functional features of the SCI interactome, and identifies a powerhouse of hub proteins representing the most prominent pathophysiological processes that occur after injury. Given that the primary injury in SCI is predominantly mechanical in nature, the curated interactome serves as the machinery behind secondary injury, which is a major determinant of outcome and recovery. We were able to analyze the contribution of different node clusters to the overall network and to interpret how the different SCI pathophysiological mechanisms interact. We also identified a central triad of three proteins that are most influential in the network. Despite the advantage of our approach in curating the entire SCI literature, mining data only from abstracts without curating additional information from papers’ results including involvement of additional proteins and the time after injury at which each protein is involved is still a limitation of our study. In the present study, we have limited our extraction datasets to the texts of the selected 4,102 abstracts without referring to other sections of the manuscript. The assumption behind this approach is that most authors mention their most significant findings in their abstract which is the reason why current systems biology databases predominantly on mining abstracts for information extraction especially when large datasets of published reports are involved. Although the time of dysregulation of proteins after injury does not affect its centrality in protein interaction network, curating data on the temporal profile of changes after SCI still allows for time-dynamic reconstruction of the SCI network disturbances after injury. Therefore, future work will aim at curating time dependent changes of protein expression or activation after SCI.

## Supporting Information

S1 Fig Raw graph of the SCI interactome. (PNG)

S1 File Individual sub-networks of the different modules within the rich-club. (PDF)

S1 Table List of annotation rules used in term curation. (PDF)

S2 Table List of systems biology tools and databases used in this study. (PDF)

S3 Table Datasets of all captured terms listed by their UniProt protein accessions and including their frequencies and corresponding references. (PDF)

S4 Table Gene Ontology (GO) biological processes over-representation in the entire 1083 accessions performed through DAVID. (PDF)

S5 Table Gene Ontology (GO) cellular components over-representation in the entire 1083 accessions performed through DAVID. (PDF)

S6 Table KEGG pathway enrichment on the entire 1083 accessions performed through DAVID. (PDF)

S7 Table KEGG pathway enrichment on rich-club accessions performed through DAVID. (PDF)

S8 Table Distribution of core rich-club proteins across biological pathways using PantherDB. (PDF)

S9 Table Weighted degrees of the different rich-club modules within the module interaction network. (PDF)
