# Supplementary material for: Molecular Architecture of Spinal Cord Injury Protein Interaction Network
Source: PLoS One. 2015 Aug 4;10(8):e0135024. doi: 10.1371/journal.pone.0135024 (PMC4524728; doi:10.1371/journal.pone.0135024)

Supplementary Tool S1

Interface for visualizing the subnetworks within each of the rich-club modules

Notes:
- Clicking on each module will show its corresponding subnetwork
- Subnetworks were drawn using STRING evidence view layout

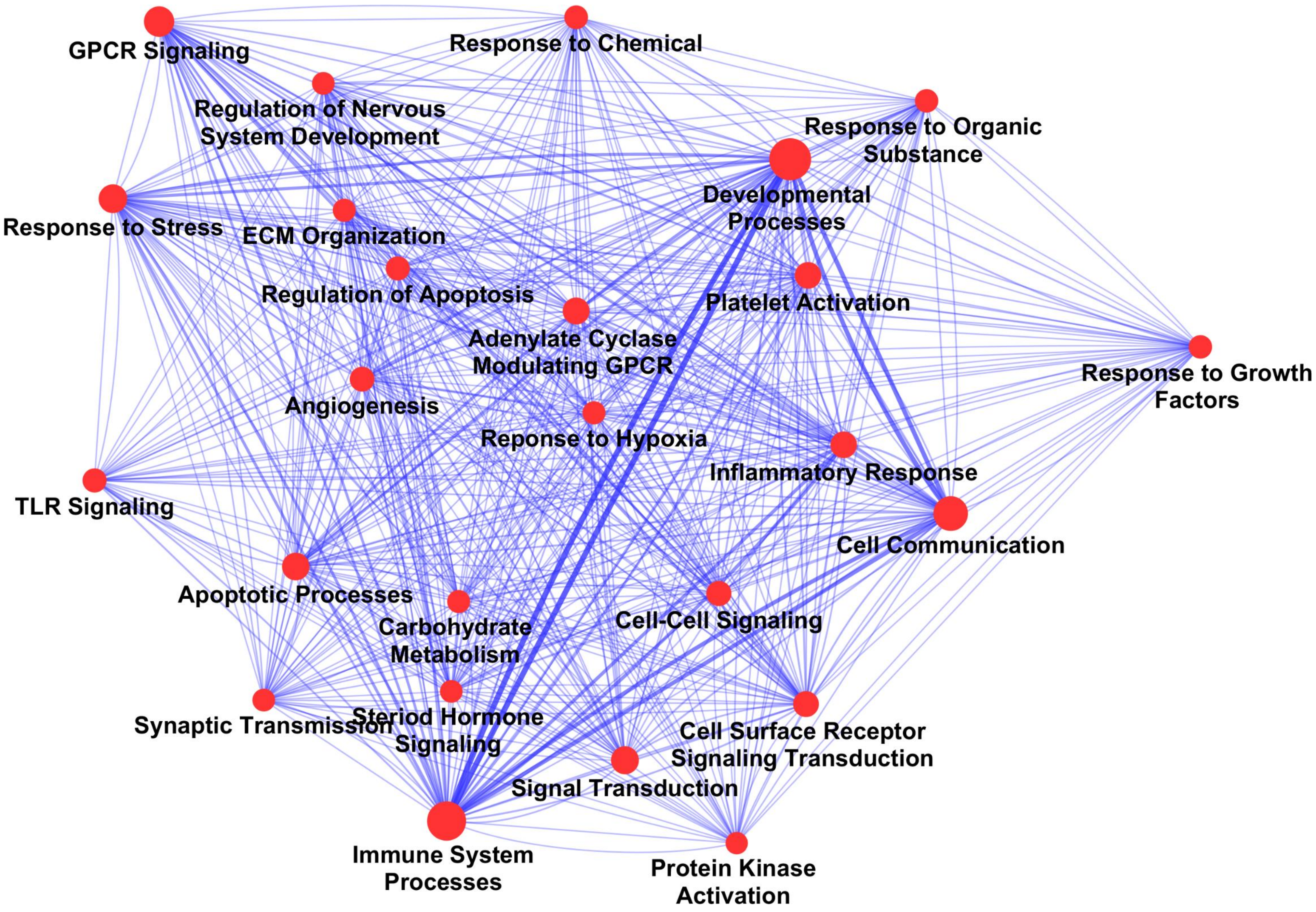
GPCR Signaling
Response to Chemical
Regulation of Nervous System Development
Response to Organic Substance
Developmental Processes
Response to Stress
ECM Organization
Regulation of Apoptosis
Platelet Activation
Adenylate Cyclase Modulating GPCR
Response to Growth Factors
Angiogenesis
Reponse to Hypoxia
Inflammatory Response
TLR Signaling
Cell Communication
Apoptotic Processes
Carbohydrate Metabolism
Cell-Cell Signaling
Synaptic Transmission
Steriod Hormone Signaling
Cell Surface Receptor Signaling Transduction
Signal Transduction
Immune System Processes
Protein Kinase Activation

MODULE 1: GPCR Signaling

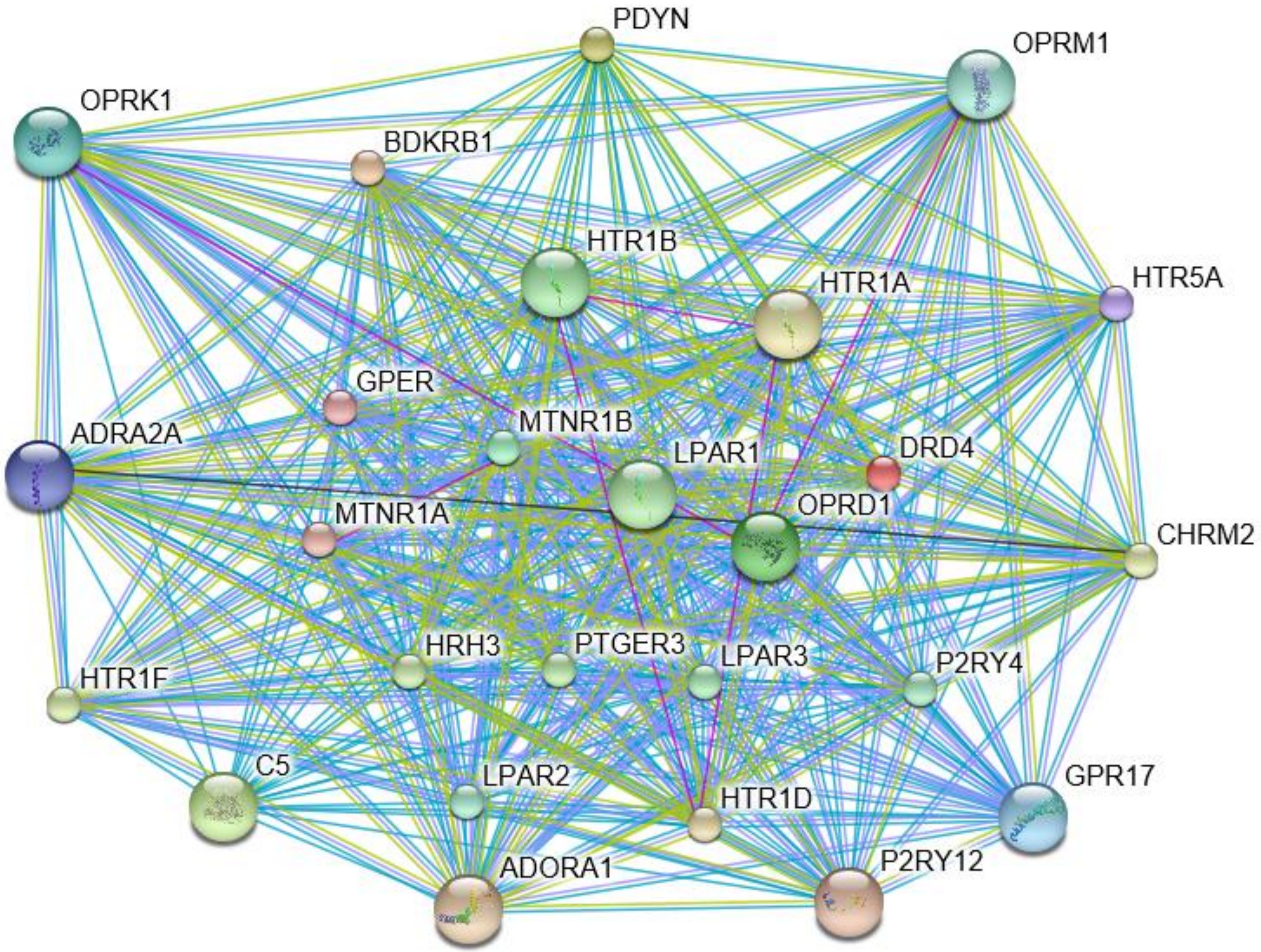

MODULE 2: Signal Transduction

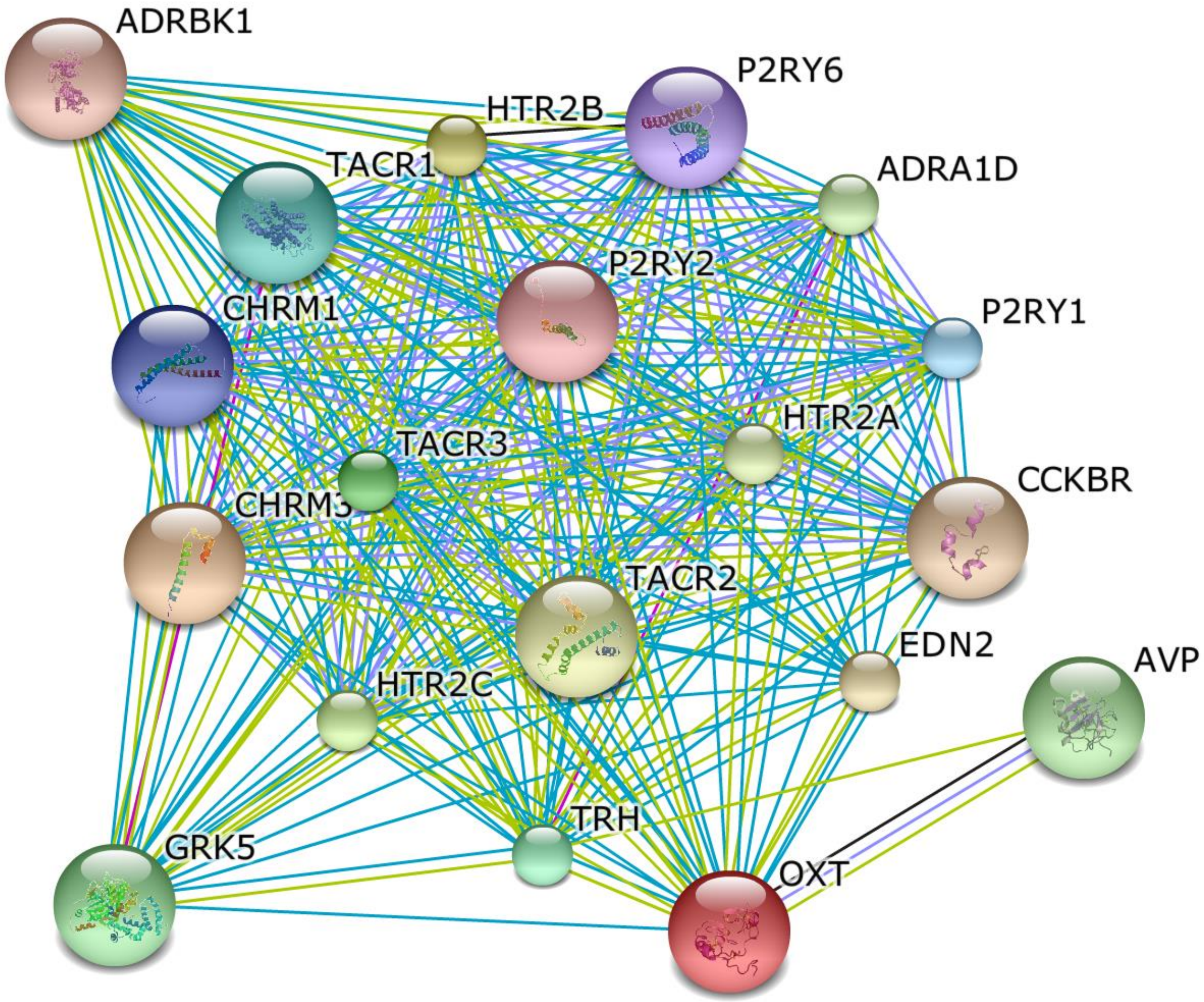

MODULE 3: Immune System Processes

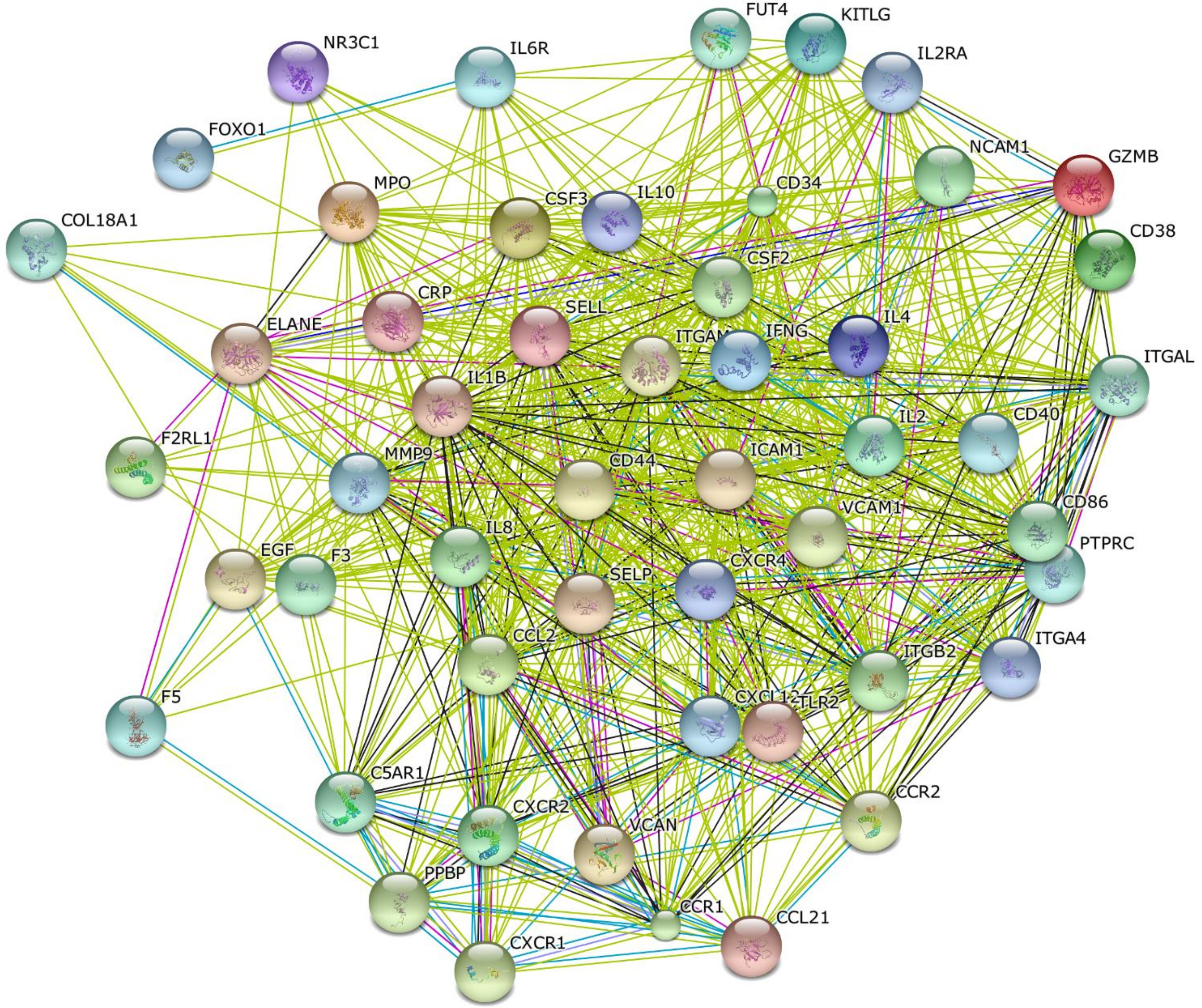

MODULE 4: Developmental Process

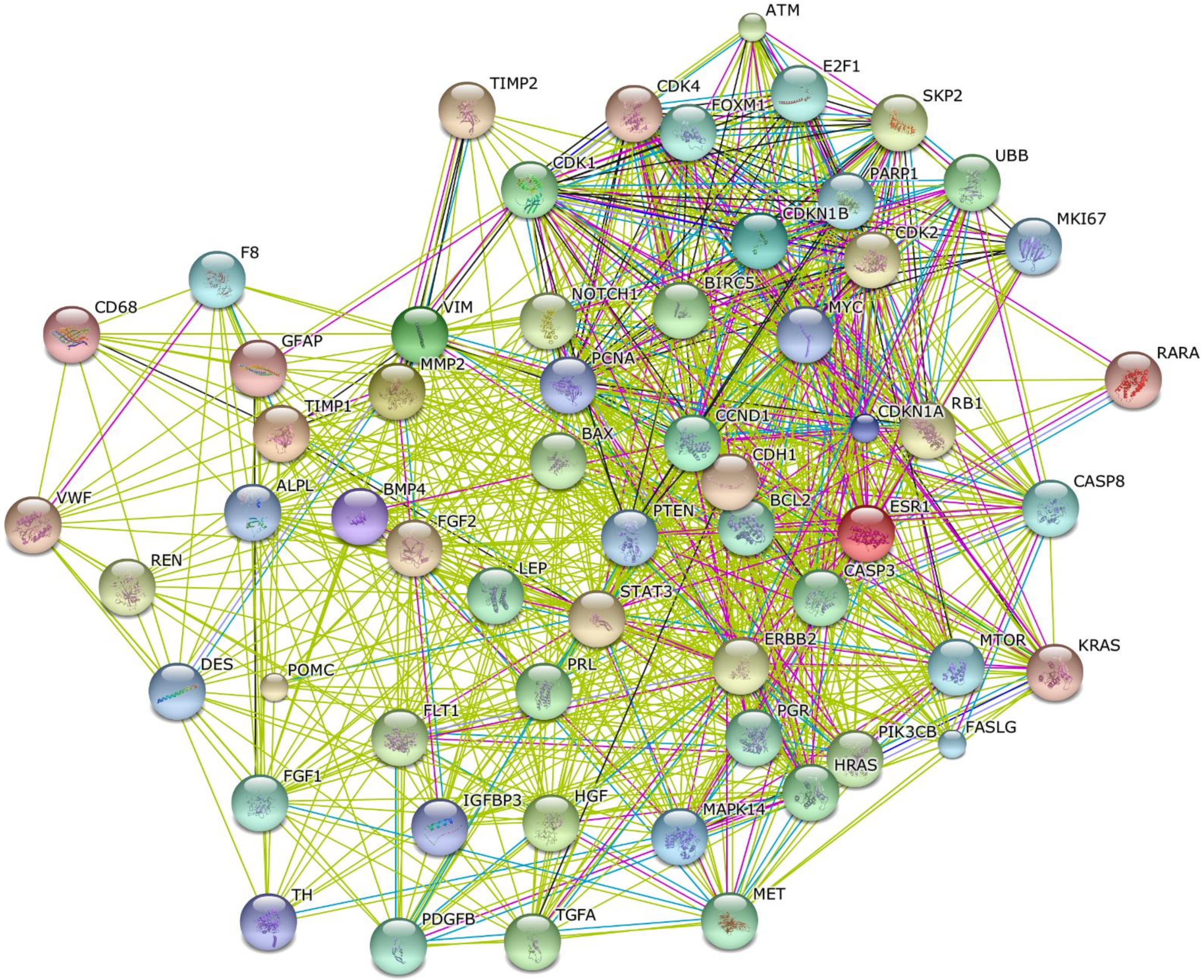

MODULE 5: Apoptotic Processes

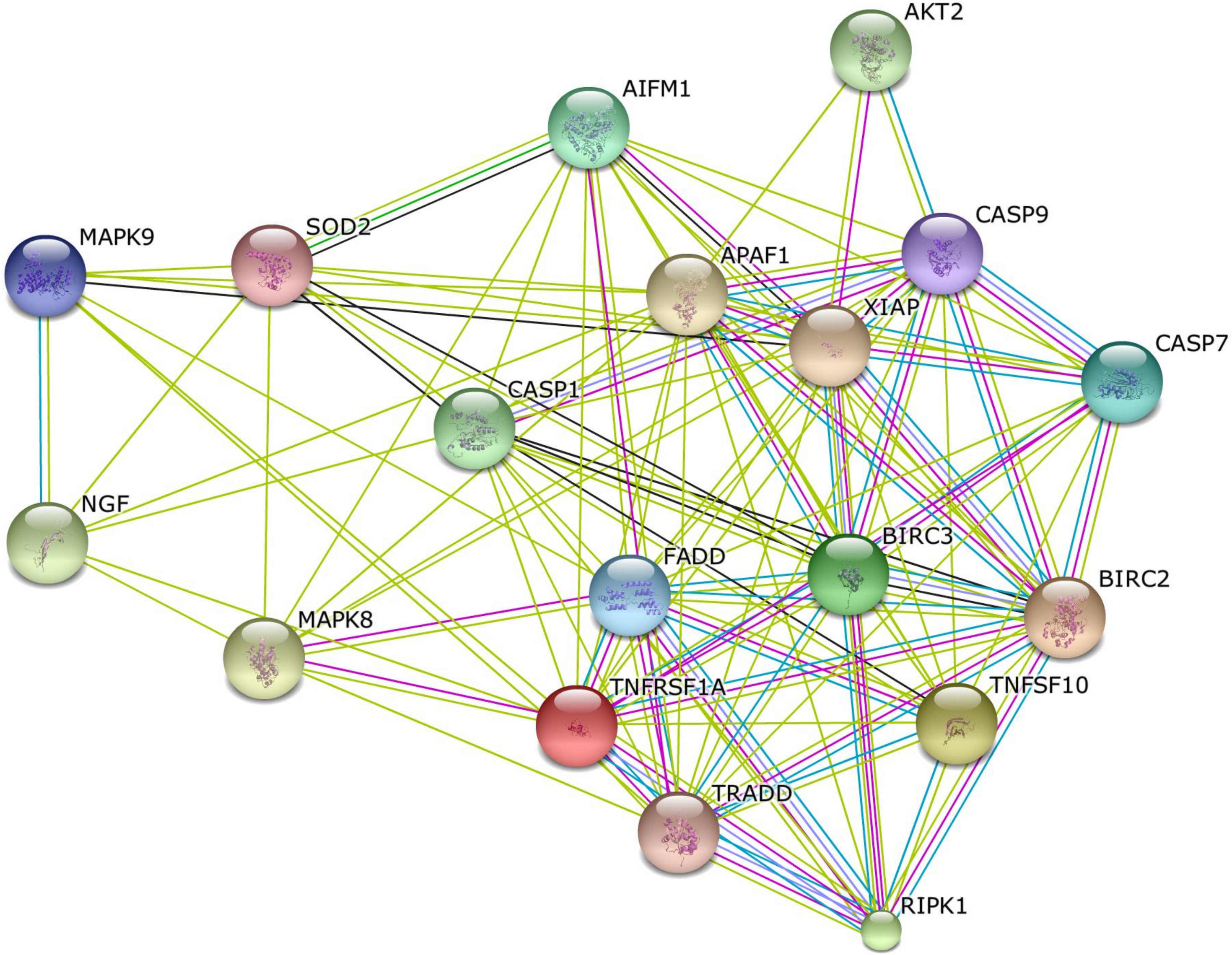

MODULE 6: Inflammatory Response

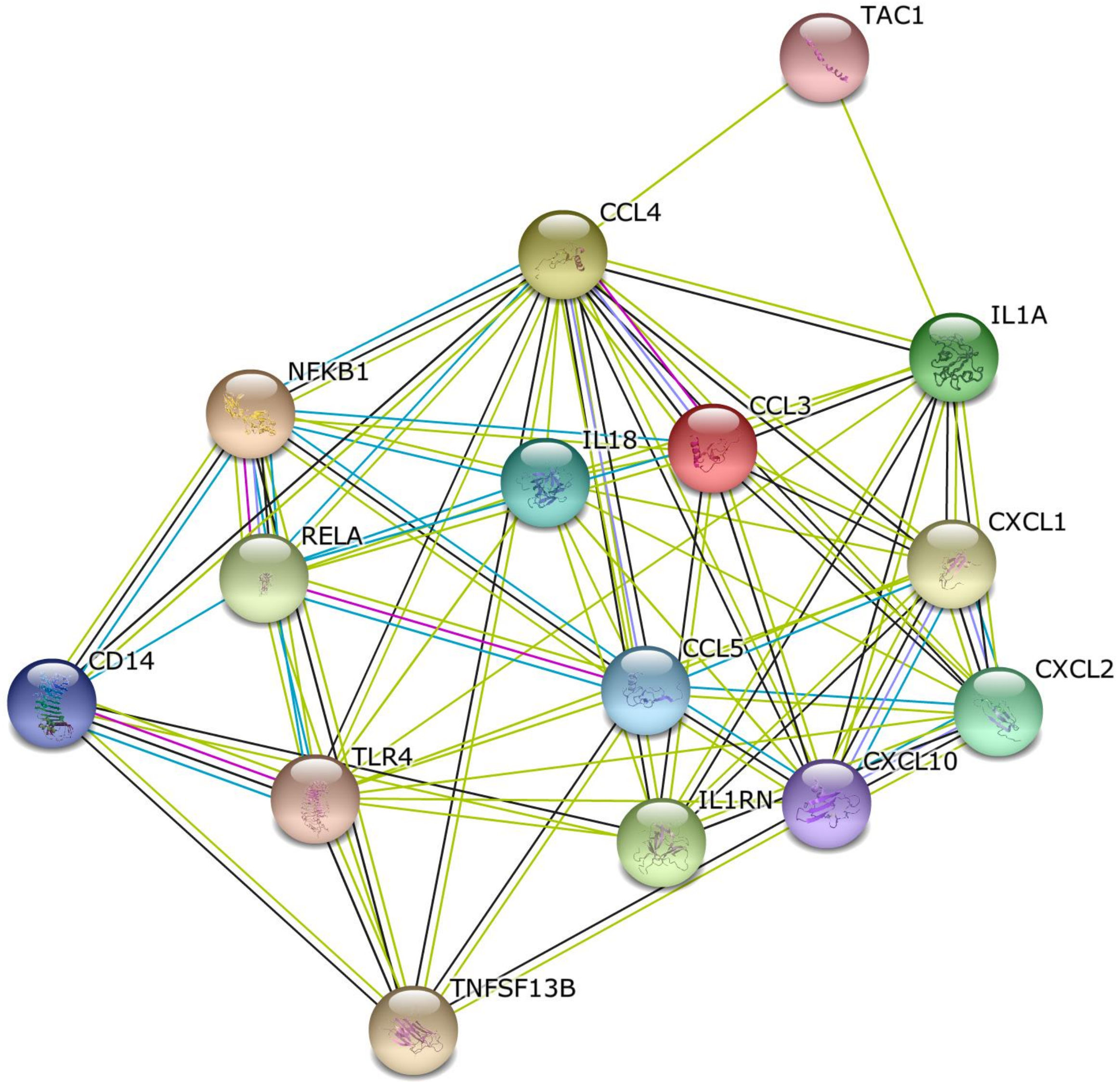

MODULE 7: Platelet Activation

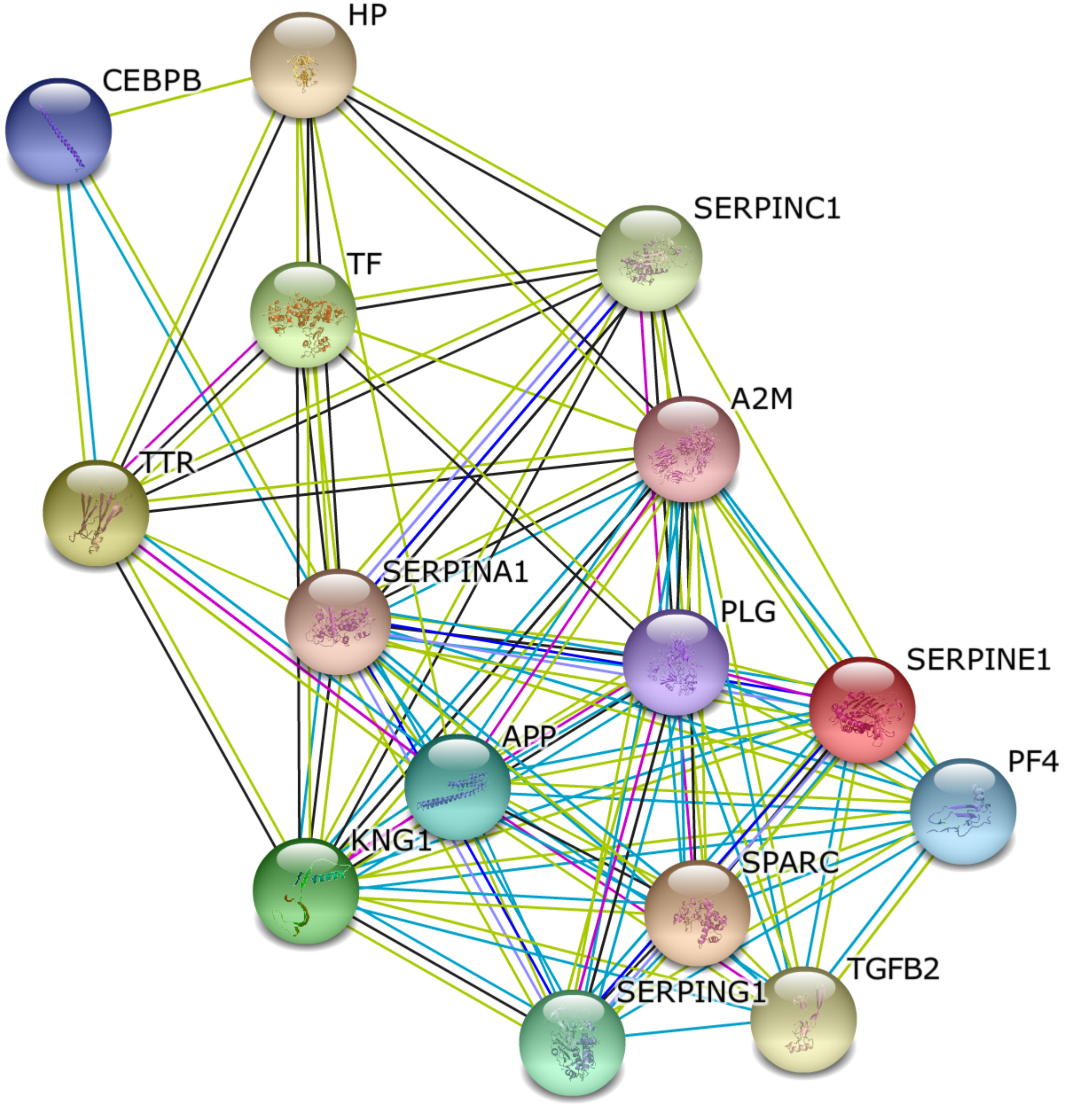

MODULE 8: Response to stress

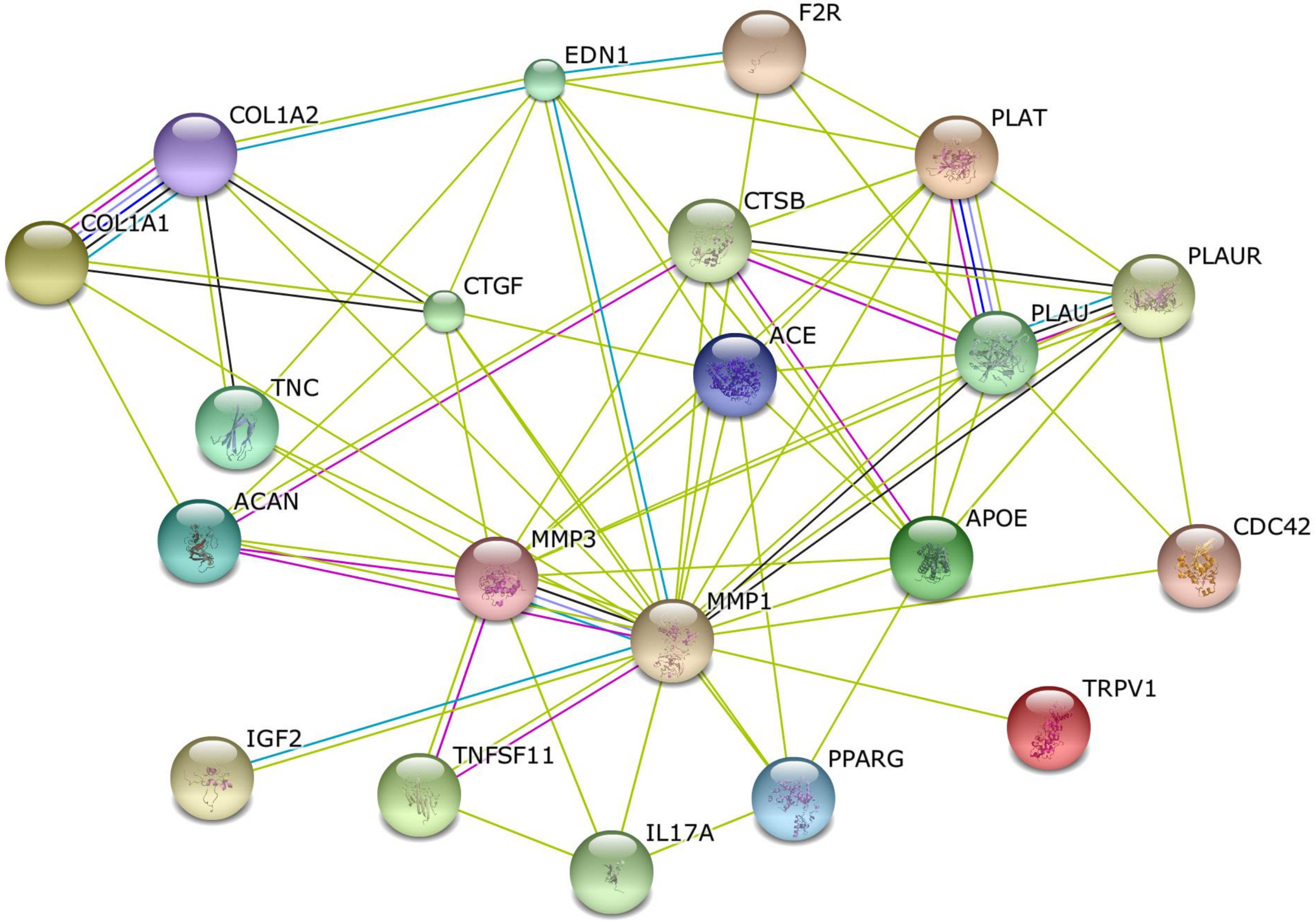

MODULE 9: Cell Communication

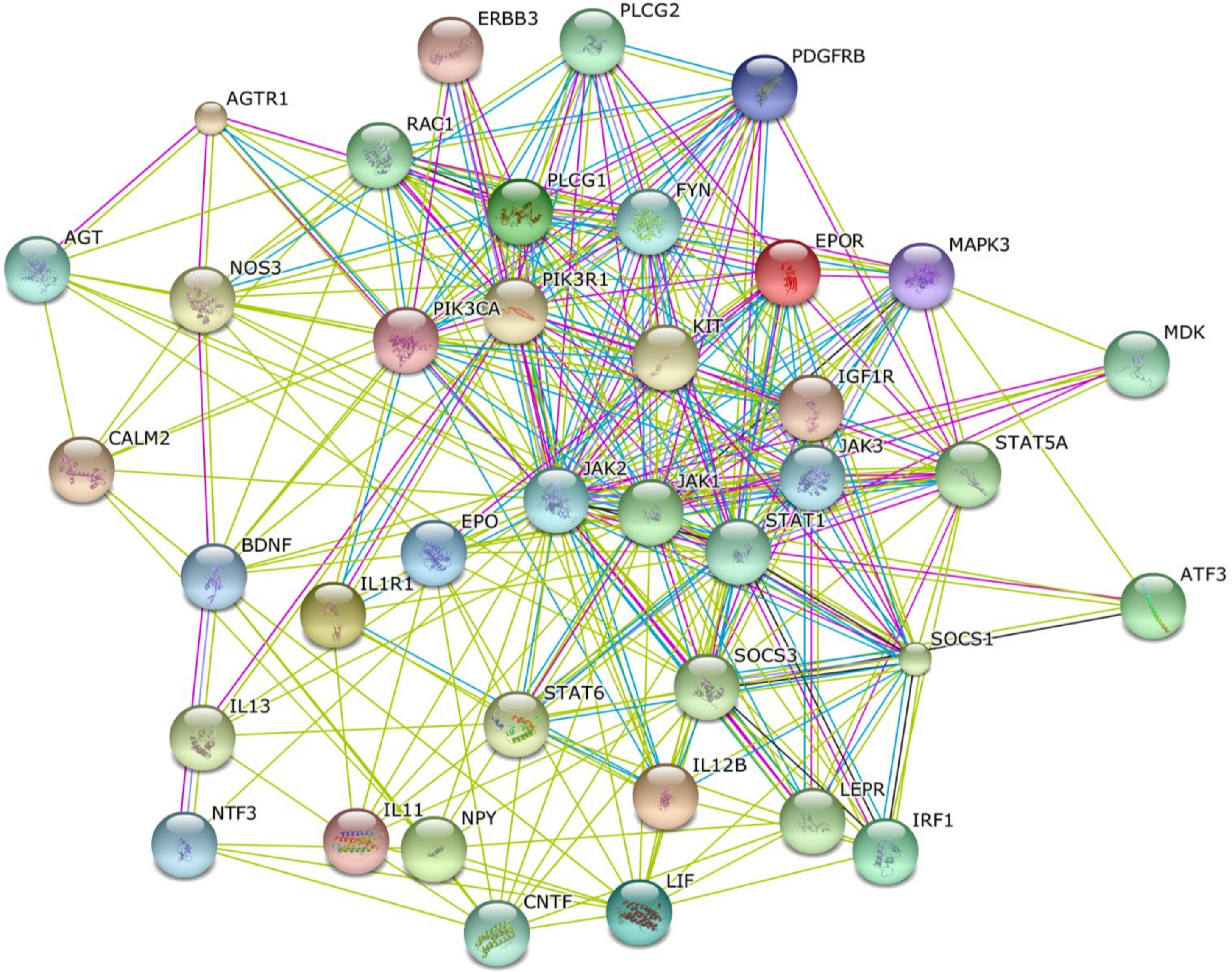

MODULE 10: Toll-like receptor signaling

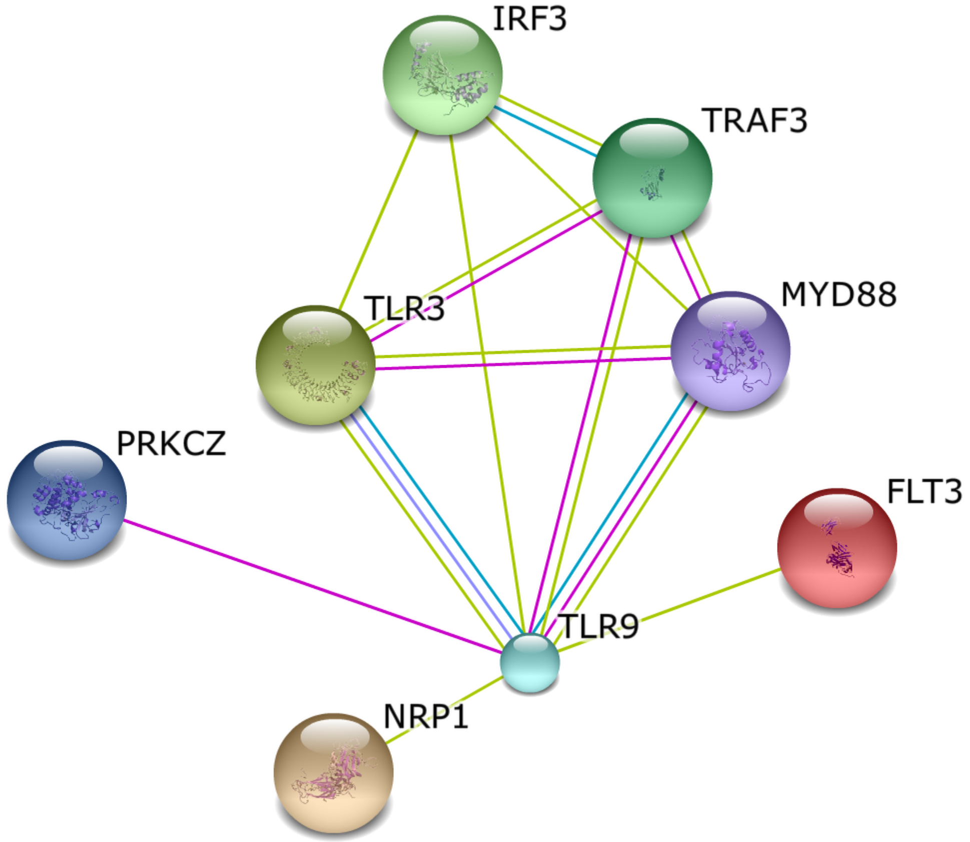

MODULE 11: Adenylate cyclase-modulating GPCR

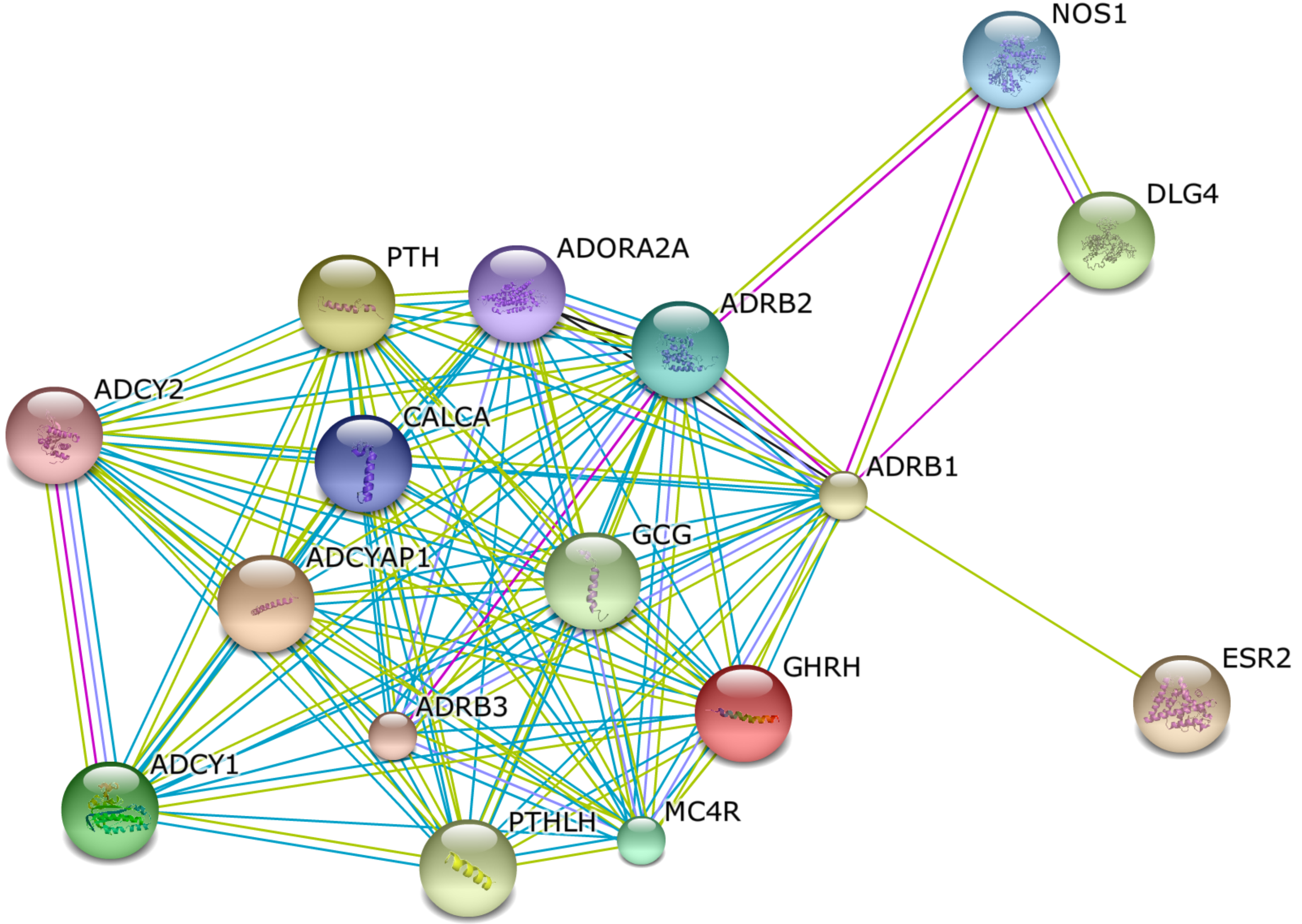

MODULE 12: Cell surface receptor signaling transduction

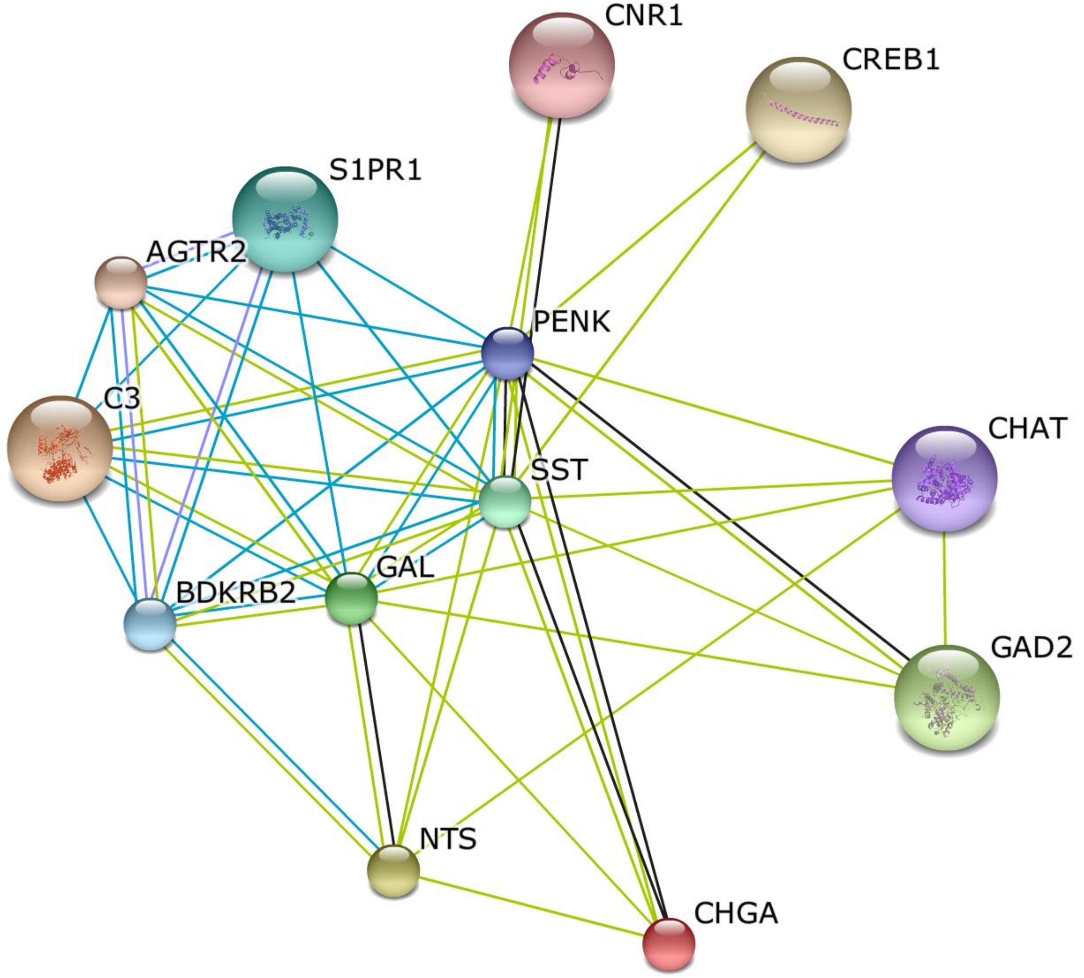

MODULE 13: Angiogenesis

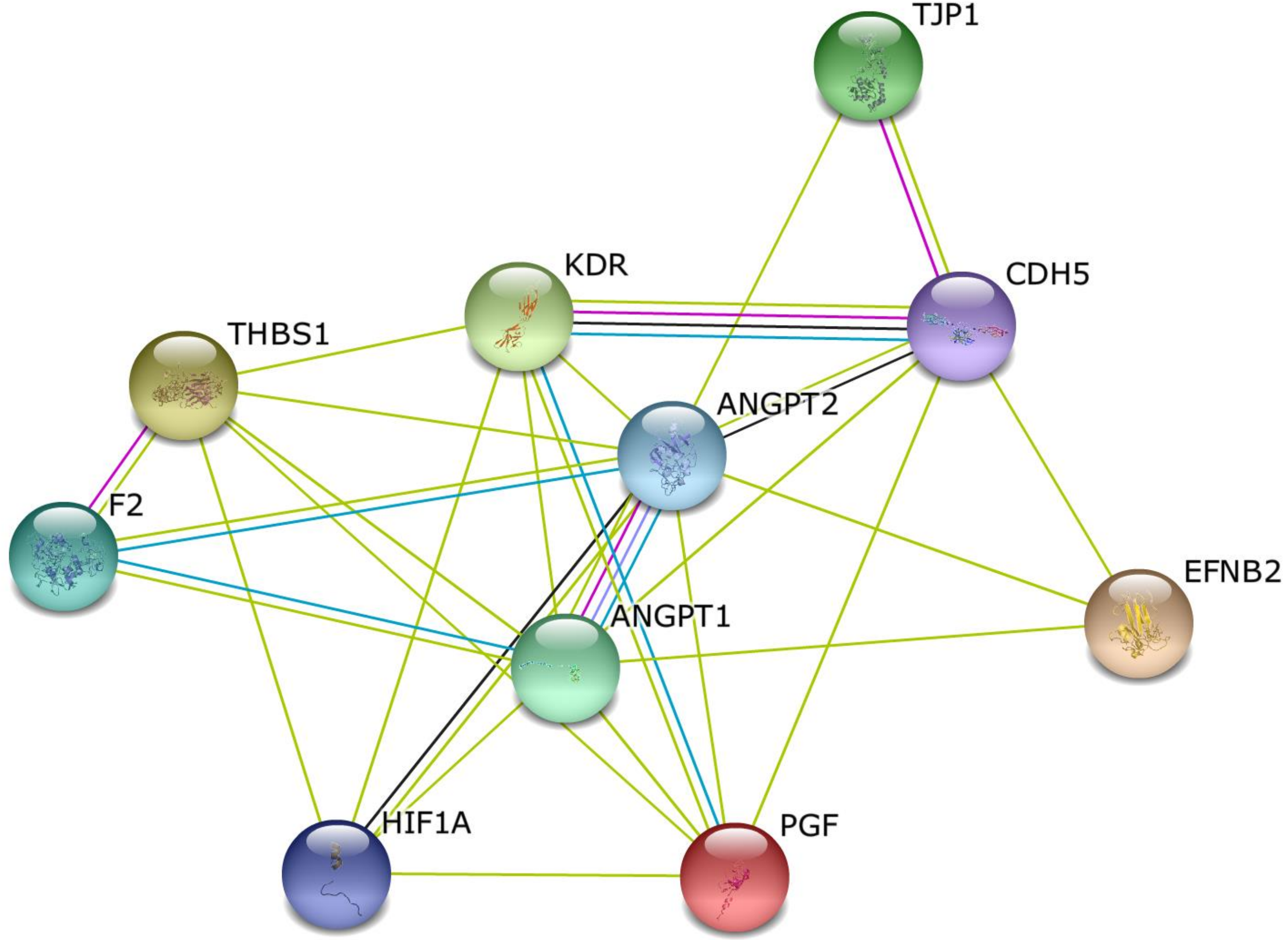

MODULE 14: Cell-cell signaling

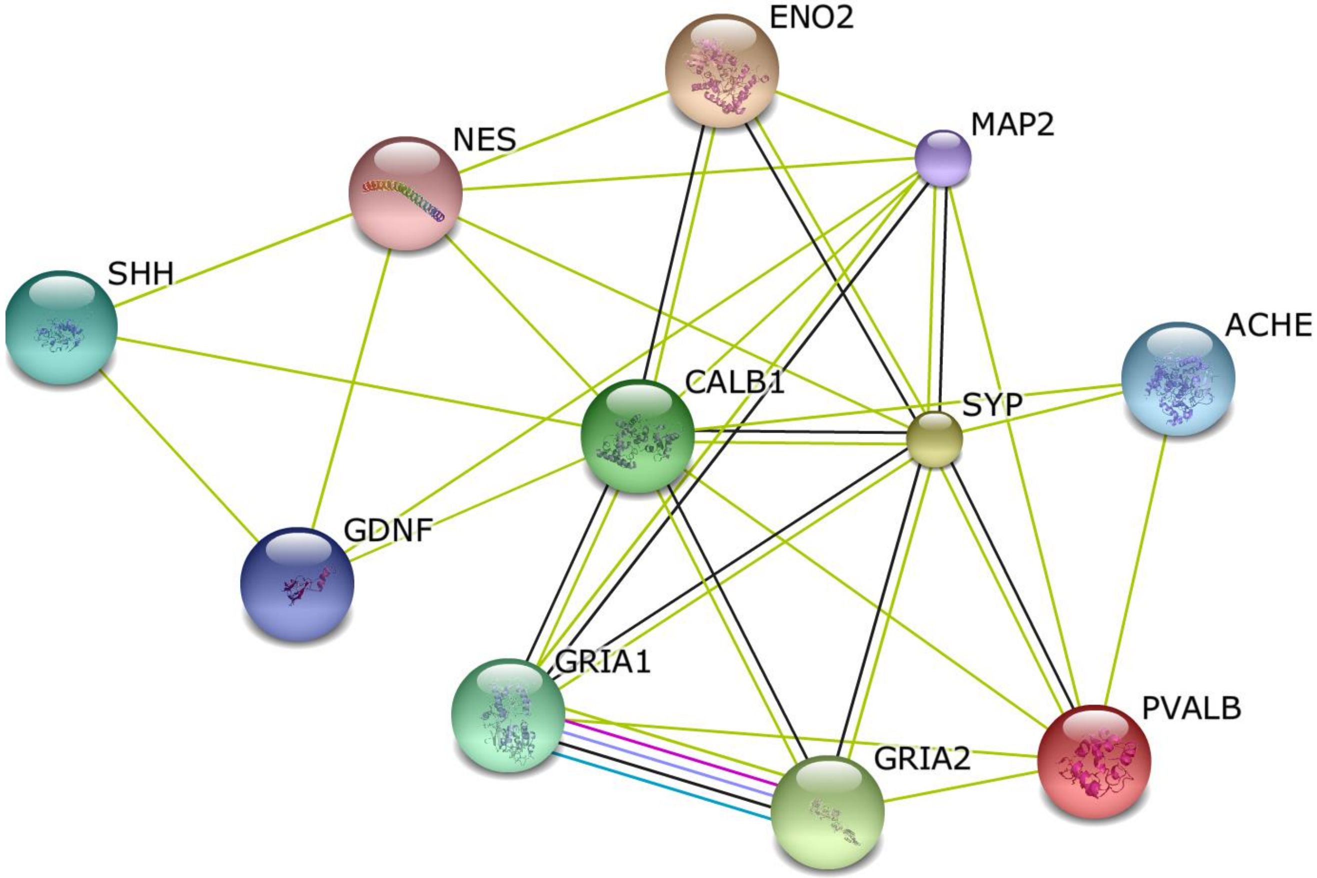

MODULE 15: Response to hypoxia

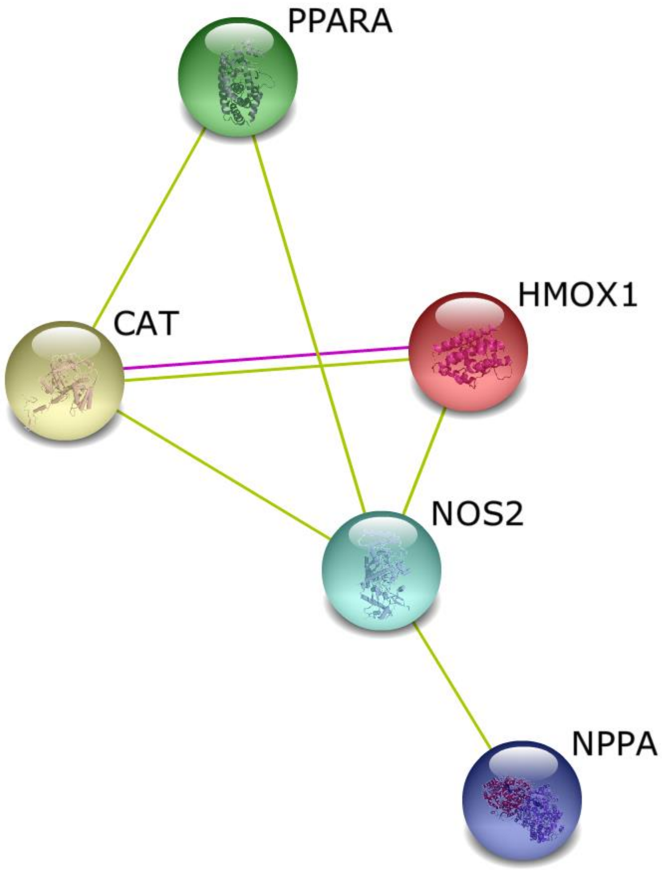

MODULE 16: Carbohydrate metabolism

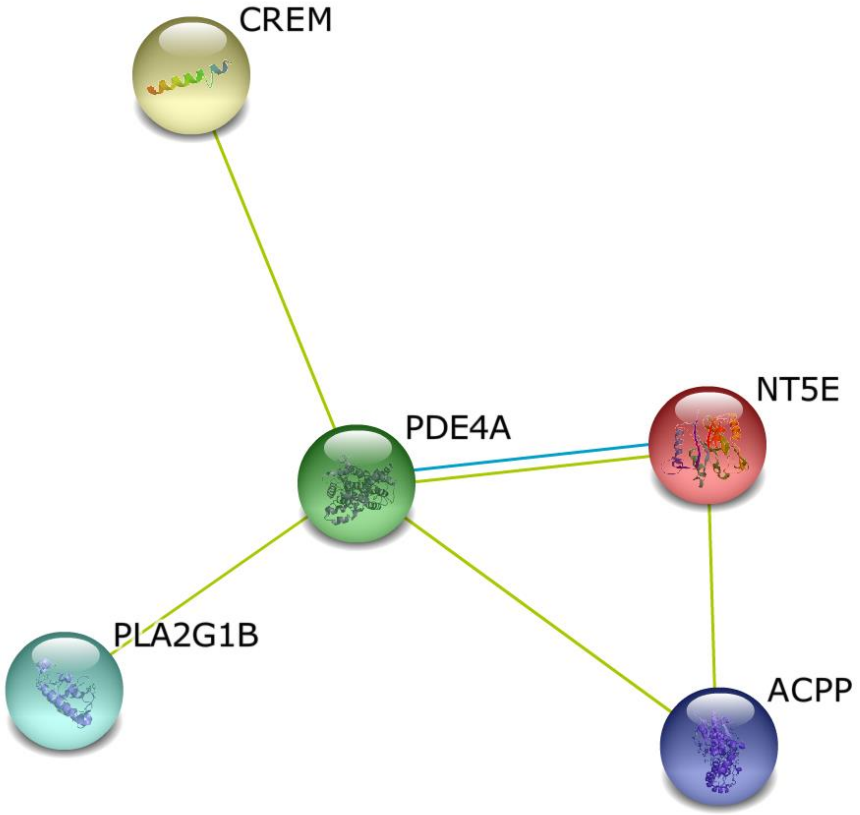

MODULE 17: Regulation of apoptosis

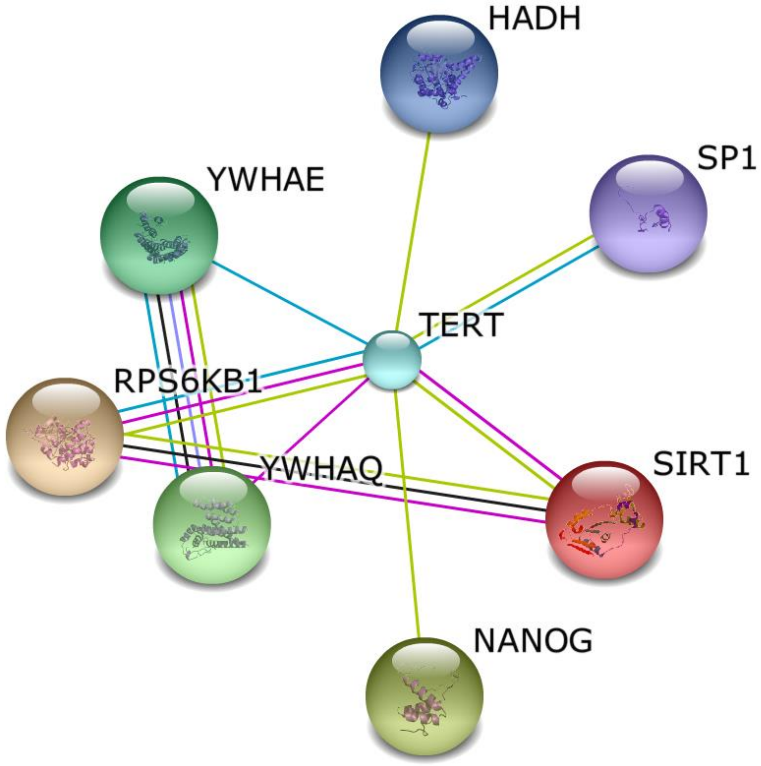

MODULE 18: Response to organic substance

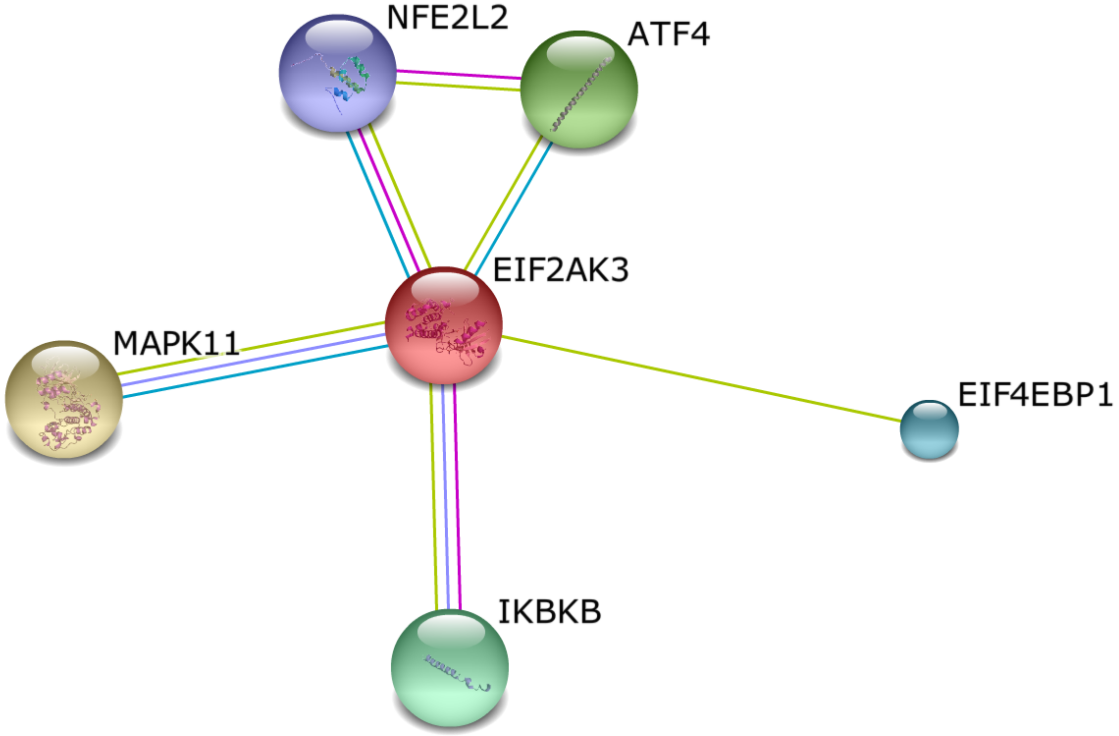

MODULE 19: Response to chemical

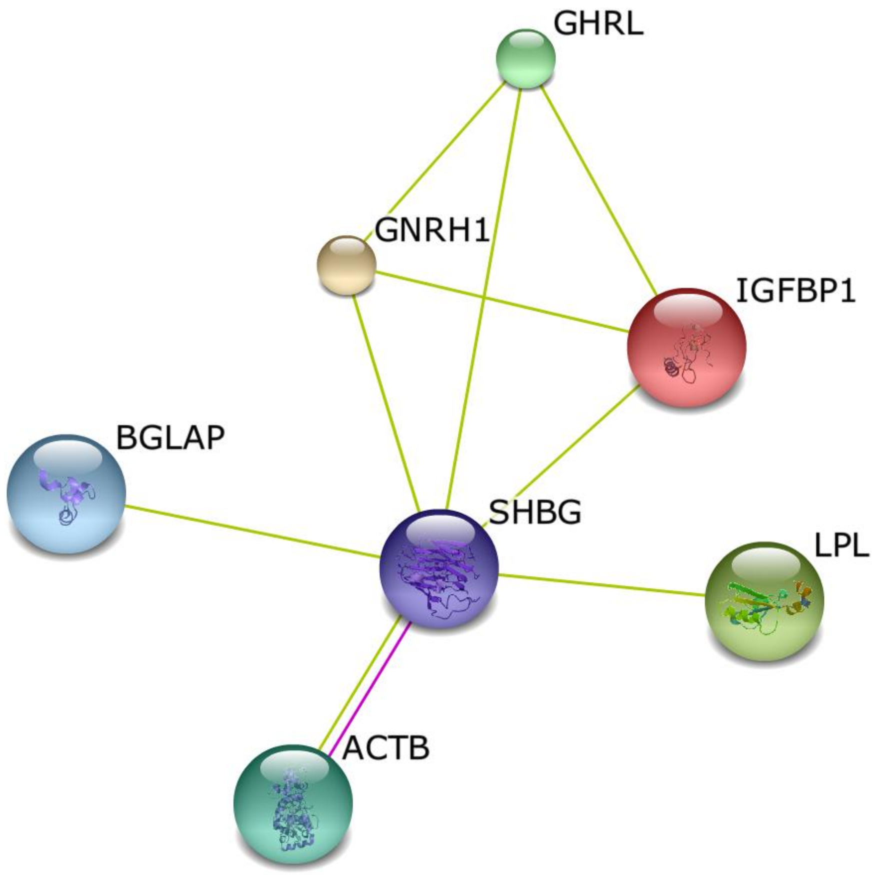

MODULE 20: Response to Growth Factors

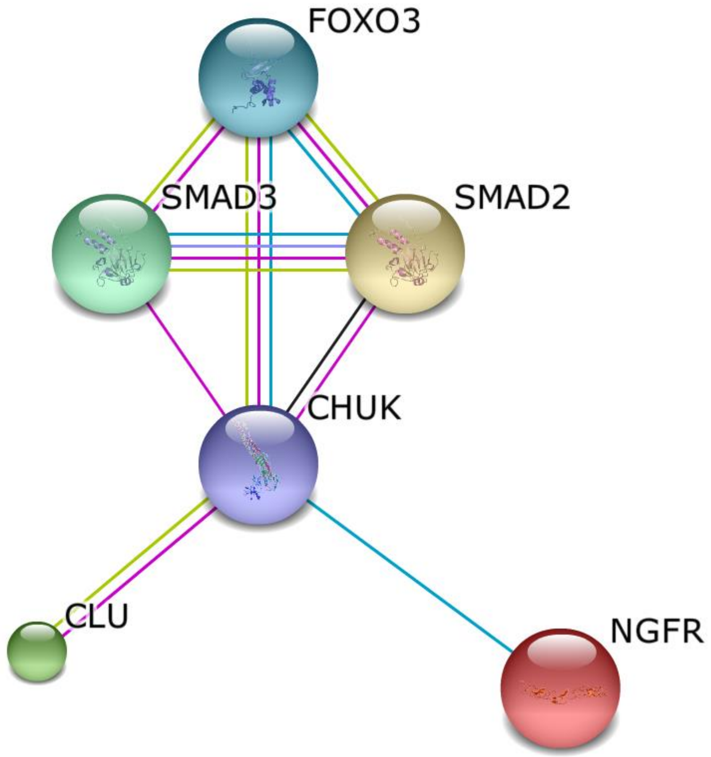

MODULE 21: Steroid hormone signaling pathway

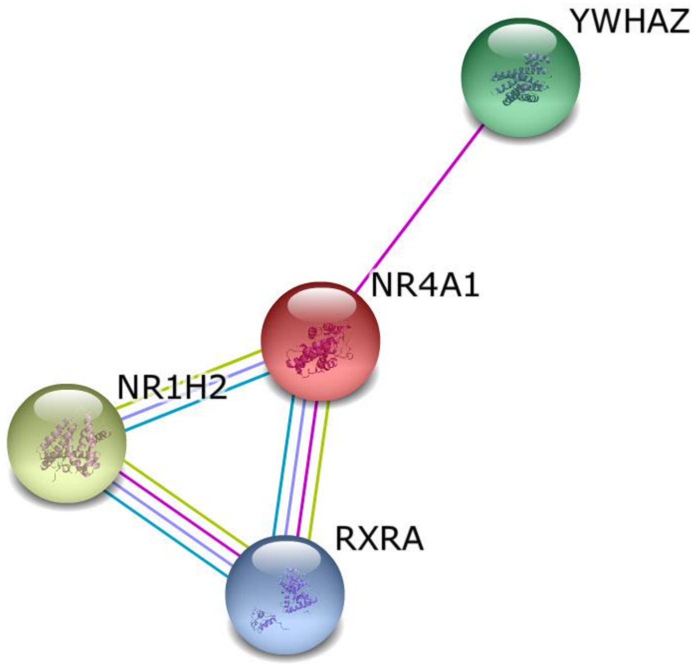

MODULE 22: Extracellular matrix organization

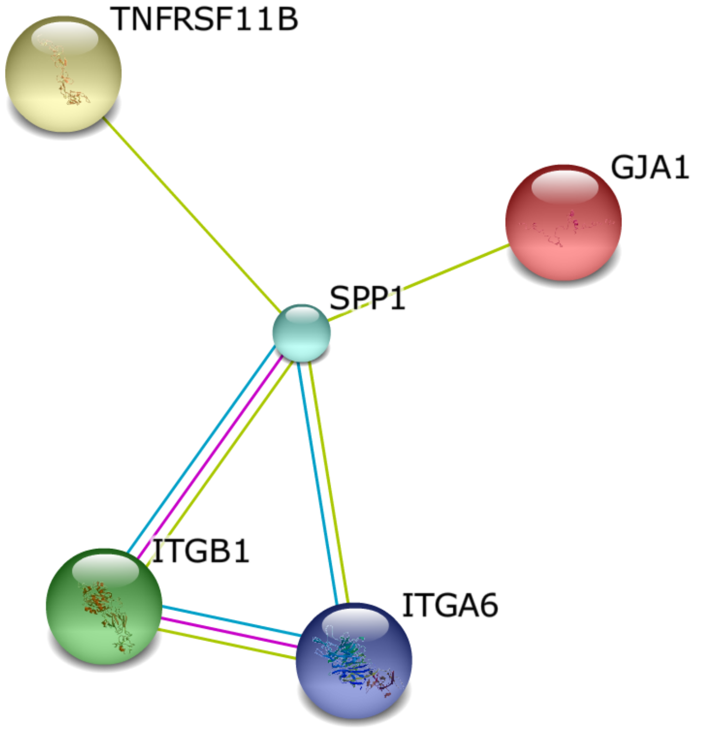

MODULE 23: Regulation of nervous system development

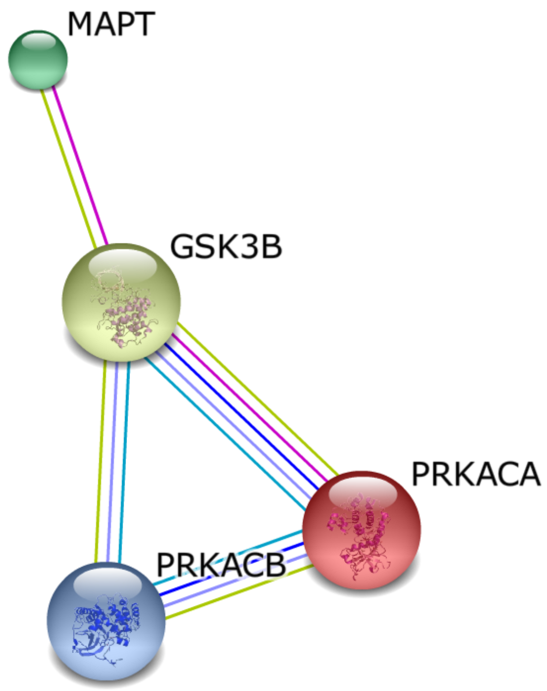

MODULE 24: Synaptic transmission

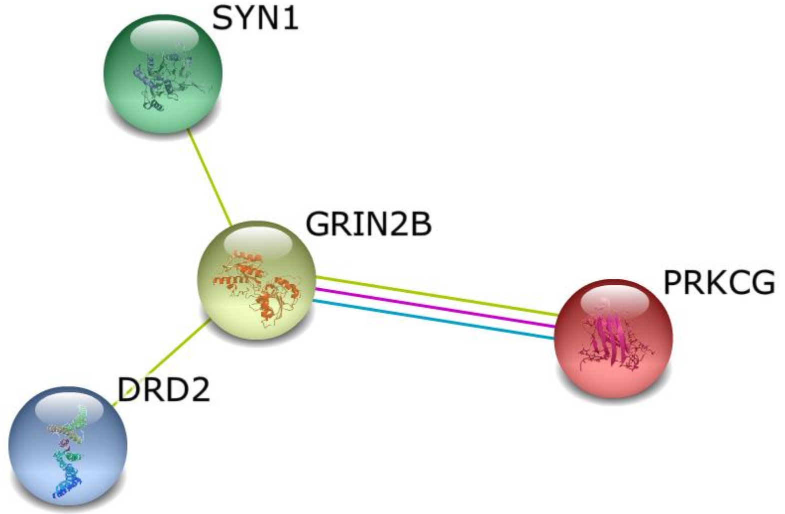

MODULE 25: Activation of protein kinase activity

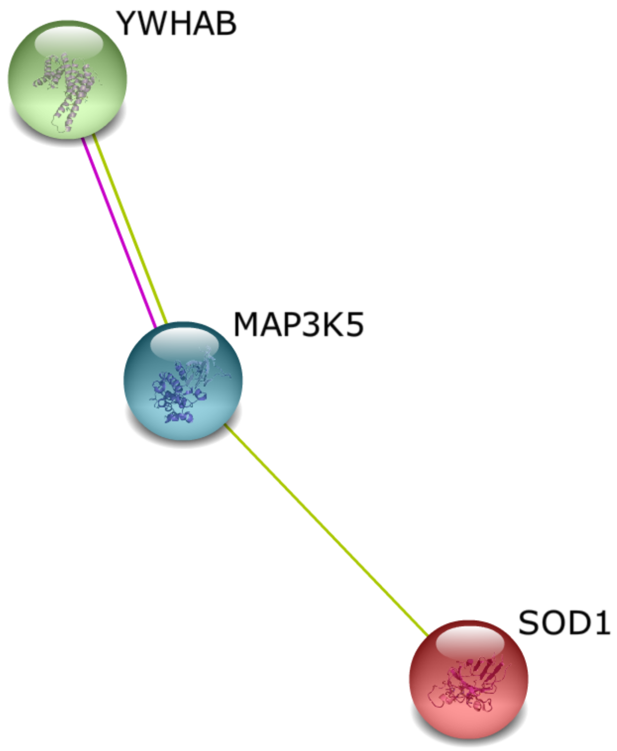

Supplement: S1 File — (PDF) [file pone.0135024.s002.pdf]
