# Supplementary material for: Molecular Architecture of Spinal Cord Injury Protein Interaction Network
Source: PLoS One. 2015 Aug 4;10(8):e0135024. doi: 10.1371/journal.pone.0135024 (PMC4524728; doi:10.1371/journal.pone.0135024)
Supplement: S1 Table — (PDF) [file pone.0135024.s003.pdf]

Supplementary Table I. Representative Set of Rules Used in Annotation.	
**Rule #**	**Description**
1	Allowing for plural and singular forms of a name; exception: NOS (for nitric oxide-synthase) does not allow for NO, MR is not included as mineralocorticoid receptor except if context is relevant (can be confused with magnetic resonance).
2	Allowing for number after the name as well as for a number series split by comma, slash or other delimiters
3	Allow for ion symbols and names alternatively
4	Allow for greek letters or single non-article letters after the name (indicate subunits)
5	Allow for use of abbreviations if they have no alternative meaning in English, and indicate those with potential meanings for manual processing
6	Allow for minor spelling changes, e.g. vowel switching
7	Allow for presence/absence of a slash, hyphen, brackets, etc.
8	Allow for detection of matches if non-spaced characters are added from either sides of the word, e.g. GFP-NGF
9	Detect terms within the context of changes in expression and highlight for manual confirmation; e.g. detect term XXX in sentence: "Spinal cord injury is associated with **upregulation** of XXX **levels** in the spinal cord".
10	Other minor rules are situation specific and indicated by the annotator.
