# Supplementary material for: Molecular Architecture of Spinal Cord Injury Protein Interaction Network
Source: PLoS One. 2015 Aug 4;10(8):e0135024. doi: 10.1371/journal.pone.0135024 (PMC4524728; doi:10.1371/journal.pone.0135024)
Supplement: S2 Table — (PDF) [file pone.0135024.s004.pdf]

Supplementary Table II. Description of tools and databases used in our analysis.			
**Database/ Tool**	**Full Name**	**Use Description**	**Link**
UniProt	The Universal Protein Resource	Used for obtaining protein names and accessions	http://www.uniprot.org/
HGNC	HUGO Gene Nomenclature Committee	Used for obtaining gene names and accessions	http://www.genenames.org/
DAVID	The Database for Annotation, Visualization and Integrated Discovery	Used for functional annotation of the curated accessions and enrichment analysis	http://david.abcc.ncifcrf.gov/
GO	Gene Ontology	Used for annotation of biological processes and cellular components for different accessions	http://geneontology.org/
KEGG Pathway	Kyoto Encyclopedia of Genes and Genomes	Used for annotation of KEGG pathways enriched in the network and rich-club	www.genome.jp/kegg/
STRING	Search Tool for the Retrieval of Interacting Genes/Proteins	Used for retreiving information on protein-protein interactions	http://string-db.org/
PANTHER	Panther Classification System	Used to obtain distribution of genes across different enriched GO categories.	http://pantherdb.org/
Cytoscape	Cytoscape	Used for network visualization	http://www.cytoscape.org/
SBEToolbox	MATLAB Systems Biology & Evolution Toolbox	MATLAB toolbox used for network analysis	http://www.bioinformatics.org/sbetoolbox/
