# Supplementary material for: Molecular Architecture of Spinal Cord Injury Protein Interaction Network
Source: PLoS One. 2015 Aug 4;10(8):e0135024. doi: 10.1371/journal.pone.0135024 (PMC4524728; doi:10.1371/journal.pone.0135024)
Supplement: S3 Table — (PDF) [file pone.0135024.s005.pdf]

**Supplementary Table III. Datasets of all captured terms listed by their UniProt protein accessions and including their frequencies and corresponding references.**		
**Uniprot Accession**	**Frequency**	**Reference PubMed IDs**
A6NDG6	5	22947335;12895449;10557125;9890271;9201240
A6NFN3	36	23526403;23386122;23180094;23054070;23001399;22999930;22683506;22484641;21807380;21336655;21215745;21039984;20678995;19818206;18855939;18649176;18627258;18484898;18008145;18001275;17671987;17284176;16978658;16897366;16850748;16004983;15995139;15790679;15672632;15129757;14727128;14697859;14697329;14570283;11893024;11161592
O00168	1	22275761
O00206	5	22520687;21970496;19932745;17403033;19688331
O00231	1	17218363
O00238	1	21764678
O00300	6	22947224;18398265;15812577;15812577;22947224;18398265
O00330	4	20438613;20392143;19119913;2352011
O00451	2	19235905;16187294
O00463	1	22484641
O00519	1	18930143
O00533	13	22510563;22473292;21337374;20381564;19170182;19150614;18820404;17626035;17611275;16240391;15341588;11212863;9671667
O00548	1	22452482
O00560	1	23607754
O00585	2	23775067;17699671
O00622	1	20860549
O00755	1	21196217
O14164	2	22289688;15478370
O14511	3	14618270;11797086;11241740
O14531	1	22181040
O14543	3	22815920;16783372;11723173
O14558	1	21655070
O14594	25	23702616;23027386;23001399;22525836;22079829;21952042;20925481;20534825;19604403;18765417;18618667;18001203;17540369;17141961;16629625;15016081;14561854;12895450;12573465;12526031;12440375;11923444;11425904;11273643;10483914
O14638	3	24206033;21676683;16301263
O14727	1	21748659
O14746	3	23793903;19908289;15798365
O14763	1	20107429
O14786	6	23116416;15737738;15094469;12927201;11029637;10192772
O14788	5	15812577;22947224;15812577;22947224;18398265
O14791	1	11170729
O14920	3	21469085;22289688;15478370
O15033	1	22161971
O15041	1	12009760
O15054	1	22578249
O15066	1	23093447

O15111	5	23499960;21134362;22289688;15478370;23880092
O15117	1	18680553
O15118	1	18751914
O15123	2	23562792;22020092
O15240	1	19846725
O15264	38	23688865;23404572;22821814;22733360;22634758;22525836;21922518;21394310;20954833;20943915;20444938;20382225;20161735;19846725;19765637;19699199;19418456;19409102;19090911;18708926;18590729;18562123;18511041;18209489;17634369;17443214;16978658;16930431;16511963;16478624;16422251;16187294;16038625;15843065;15320513;14512145;12810381;10331432
O15392	1	16230180
O15399	72	22159095;21995852;16289170;23618680;22650041;22650040;22449374;22402659;21756907;20675200;20495588;20211179;19939961;19923273;19772458;19668258;19407255;19026989;19019202;19005051;18635178;18484790;18177675;18053028;17983769;17287199;16703907;16678969;15950221;15901762;15748877;15640756;15313031;14758350;12903514;12885421;12162901;11992467;11107569;11101210;11043549;11007881;10984543;10720616;10674755;10486193;10419375;9918969;9773443;10921077;9048311;9002066;8985951;9594166;8880851;8657340;8584148;7472559;7889371;8207481;7909561;8137170;8103243;1588610;1311880;1837648;2163498;2155794;2377900;1982014;2841902;3057216
O15455	1	19688331
O15496	1	19306380
O15516	2	11224015;10811391
O15524	1	18205174
O15530	1	22749575
O15540	10	22469052;21931744;21574060;20155816;20151365;19418545;15869942;15548666;14644473;12098653
O43396	1	22466292
O43474	3	21949375;20030221;18712509
O43490	5	23498368;22246536;20806064;20374199;20029964
O43524	3	22930444;19533653;22930444
O43741	2	22865656;17885021
O43772	1	23011062
O43916	1	20427653
O43921	1	21603973
O60259	1	24128681
O60266	6	20141154;19119913;18677446;15698618;10804204;3548963
O60344	1	17259328
O60391	69	23618680;22650041;22650040;22449374;22402659;21756907;20675200;20495588;20211179;19939961;19923273;19772458;19668258;19407255;19026989;19019202;19005051;18635178;18484790;18177675;18053028;17983769;17287199;16703907;16678969;15950221;15901762;15748877;15640756;15313031;14758350;12903514;12885421;12162901;11992467;11107569;11101210;11043549;11007881;10984543;10720616;10674755;10486193;10419375;9918969;9773443;10921077;9048311;9002066;8985951;9594166;8880851;8657340;8584148;7472559;7889371;8207481;7909561;8137170;8103243;1588610;1311880;1837648;2163498;2155794;2377900;1982014;2841902;3057216
O60462	1	15094469

O60603	4	22520687;19932745;17403033;19688331
O60609	2	19235905;16187294
O60674	10	23274522;15716400;10762348;22815920;20155820;17131417;16957585;16890196;22715999;20161735
O60706	1	17108688
O60733	11	22581384;21142140;20127525;18755070;17917587;16566026;16498630;15248295;11499402;8522975;7473799
O60760	3	22520473;21294159;19159657
O60840	5	17448606;14753480;10924962;10222112;9007541
O60938	1	22114278
O75015	1	19770513
O75093	1	16262652
O75094	1	16262652
O75116	22	19955379;19623163;24297045;21815784;19623163;18722369;17217414;18980476;22733360;20141964;19955379;19651108;17692017;16624299;16154567;15880494;15799964;15293235;15219678;12480155;12151536;11471206
O75173	3	22420304;11425904;23562508
O75293	1	20702718
O75326	1	20645410
O75460	3	22873727;22815920;21638341
O75508	1	21638341
O75593	1	17538368
O75616	19	23758347;23548992;23229514;22850839;22658000;20838402;20593987;16629619;16383250;16122000;15561548;15291017;15264793;14988583;11893023;7526456;1675444;7069836;1160690
O75762	1	21367919
O75840	1	22529377
O75888	1	23088438
O75899	5	18495826;23904624;22721766;22449374;16866624
O76074	18	23347150;22925748;22837080;22275187;21970599;21036463;20157304;19875292;19686427;19210710;19138363;19057138;18595542;17083398;16198718;14671658;12767372;11402584
O94759	2	23828570;23512594
O94788	4	17671987;17239557;16688771;15845083
O94813	6	19843094;19783284;18377713;17978191;16262652;19783284
O95025	1	12009760
O95096	6	19318112;19257808;19053058;17600517;17108169;14648541
O95140	1	23727406
O95185	1	16998900
O95249	2	19286942;17933964
O95452	3	22951907;22399755;22951907
O95631	6	20132768;18377713;18234888;17978191;16998900;16262652
O95786	1	22161971
O95831	6	23828570;19473058;19120440;17298387;16689664;15857303
O95847	1	18534681
P00338	14	23793797;23647384;21529317;19998478;19485254;12852839;12509801;11417439;9362426;8145266;1984104;2718797;6526578;6822753

P00367	1	19119913
P00390	2	16503801;11149912
P00395	8	20438613;20144890;15236240;14614091;11711863;9165429;2358556;6246648
P00403	8	20438613;20144890;15236240;14614091;11711863;9165429;2358556;6246648
P00414	8	20438613;20144890;15236240;14614091;11711863;9165429;2358556;6246648
P00439	3	21087927;20714874;12598950
P00441	58	21946609;21078175;20005223;18947433;18722523;18352832;17457363;16689664;15896972;23523995;21111721;20817464;20364349;19875292;19563509;19473058;18407497;18289003;17486444;17184187;16689664;16634331;16332351;16187294;16095570;16018585;15864722;15792522;15684769;15610928;15207346;15036352;14767765;14666020;14644473;12657369;12213642;11977524;11853017;11453434;11200253;11032904;11011974;10815757;10437099;10322962;9726263;9680175;9313901;8973665;8915909;7478738;7701506;7841663;1414256;2058414;2244484;6181489
P00450	2	19036966;1707599
P00451	9	19853891;12472582;7969830;1732896;3149480;3925930;6813814;6769466;391991
P00488	2	7964707;1514887
P00533	17	23758598;23591390;22863681;22824323;22078761;22016551;21402118;20869112;20734425;20709162;19176818;18442870;17907381;17567803;22824323;23758598;23097328
P00734	26	23832758;23647384;22037947;19853891;19247855;18555151;16344894;15684771;15320513;15307903;14691582;12472582;11186232;11168298;10744154;10329979;9454848;9238054;9076865;8552614;7964707;8259328;1290164;6712454;4427781;9076865
P00738	1	1707599
P00740	1	11554943
P00742	2	14691582;9454848
P00747	9	22079561;22016526;18000864;16629625;12614587;7690062;1514887;21573723;19651246
P00748	2	7964707;3925930
P00749	6	21573723;19651246;18612314;2046844;21573723;19651246
P00750	22	21573723;19651246;22079561;22037947;22016526;21573723;19651246;19443933;19152029;18042398;18000864;16917839;16139333;12614587;12166004;9076865;7616284;7690062;1903560;2508383;21573723;19651246
P00751	2	20952585;15684772
P00797	8	21850517;9232358;8779866;7840317;8379845;2989637;7021043;7204569
P00813	3	20456005;15610928;11320597
P01008	8	9076865;15792522;15684771;15094537;14691582;9076865;3675178;3924003
P01009	1	7690062
P01019	8	21510818;20577118;18712059;7576312;2969340;3819838;2455198;7299410
P01023	4	23867460;7690062;2430066;6206205
P01024	4	23033813;20800648;16936276;16936276
P01031	5	23855891;23033813;20085927;20952585;18578885
P01033	6	21287269;19780197;16344894;12098653;11095516;10716770
P01034	4	22547045;16154555;12880536;12235535
P01042	10	18951883;18255230;15331040;12941374;12706237;11516566;7964707;1588616;1861161;3925930

P01100	47	23022460;22211901;21792724;21430166;21376061;20599980;20528165;20497419;19940184;19161992;18849175;18632941;18581599;18524486;18512692;17881483;17767492;17584182;17531342;17201484;17161529;16671479;16626634;16360182;16198697;15476702;14694501;12144050;11906794;11597598;11532430;11450029;11438395;11421586;11331375;11150324;10896894;10775883;10757326;10642858;10461813;10188945;9272824;9221952;10921077;7702707;8152541
P01106	3	21949375;20030221;12666113
P01112	1	21510818
P01116	10	23602967;20816819;19176818;18219571;16460709;15880494;15229242;21510818;15668227;22865681
P01127	18	22023610;20860549;19236168;7657809;7657809;22407783;22236767;21568693;20819515;19818206;19383401;17597120;14534257;10329979;9618703;8963994;7657809;8091423
P01130	1	23867460
P01133	22	22865681;22629425;22525836;22407783;22236767;22085254;22078761;21402118;20130176;19442692;19176818;18191945;17618992;17610915;17294724;17108169;15899248;12557207;11893024;10960593;10511239;8091423
P01135	2	18647603;17207742
P01137	55	23884949;23727390;23064509;22085254;21787762;21294159;21273111;20946170;20860549;20645410;19744530;19719963;19625042;19342245;18428026;18191945;18040277;16498024;16038877;15380482;15145083;14760011;14552879;12127673;10785461;9682014;9417823;8834102;8742129;8091423;23727390;23562792;22079829;22016551;21787762;21760535;21273450;20623539;19719963;19342245;18581269;18293403;18221366;17611274;16498024;15380482;15145083;14637102;14552879;12127673;12056841;8963994;8834102;7846578;8091423
P01138	171	24490534;23792206;23791854;23303920;22878912;22745769;22723636;22573254;22220508;22137336;22116043;22085254;21819712;21733076;21620945;21411654;21209491;21049601;20668234;20419536;20334467;20167291;20144886;20029964;19812967;19784741;19476208;22009833;19337830;19286942;19170182;18990142;18695504;18602914;18585435;18512692;18484898;18077567;17936749;17634369;17599430;17522325;17459471;17405147;17392476;17387687;17335812;17055159;16909619;16860320;16850748;16631308;16540569;16470209;16198706;16198705;15649491;15639807;15522871;15236239;15191808;15128857;14665959;14561854;12895449;12820682;12781986;12542855;12499054;12435430;12408842;12394773;12162901;12115695;12115676;12039665;12034080;12034073;12009779;11606629;11585248;11550223;11331375;11358454;11286165;11241740;11186234;11060810;10757326;10683293;10646525;10495438;10460247;10369555;10212315;10208566;9867998;9670992;9671662;9669765;9651218;9588600;9486767;9417824;9391013;9344570;9171168;9165951;9867940;9603065;8930993;8963994;8762435;8787152;8566208;8762551;8594213;7555254;8914793;7841375;8015371;8114912;7505409;8457890;8457883;1333605;1313859;1664273;1933308;2177175;1690226;3437471;3858886;6525501;6502217;23303920;23360280;23124646;23098734;21430166;21421025;20819171;20600315;20554000;20171177;19118106;18095482;17686909;17584113;16629625;16305315;14699980;12589927;12440384;12440372;12440358;11923434;10811391;10384258;9932426;9369296;9199395;8993706;15901762;15748877;15144859;15032708;107635
P01148	5	22480691;7531902;8487676;1700405;395062
P01160	1	1598176

P01178	5	19648936;15954070;15662938;9845225;7576312
P01185	16	21850517;17168344;17021585;11507005;10369169;10341867;9161360;8927412;7753575;7840317;7869058;8379845;2258733;3954565;6587004;7204569
P01189	26	21036396;20709555;19289322;17628936;16296909;15349736;15116065;12215892;10914354;10640453;10453760;10391370;10225218;9226335;8971834;8962601;8835031;8988397;8762550;8216840;1466870;1640843;1331272;6410971;6293405;4323244
P01210	7	17767492;15952166;11440816;11166985;10924963;8361583;2460195
P01213	31	22748825;20675200;18835424;17881483;17698296;16671477;16144654;15574363;12373547;11440816;11438395;11166985;10924963;9674552;8758278;8762550;8552782;7702707;7909561;1356430;1359484;1640843;1627280;1980130;1967925;2460195;3106200;2884046;2860844;2860614;6657511
P01222	4	17494102;1635789;6619835;7112391
P01225	19	19474795;17494102;17298549;16088035;12830973;10952914;10067862;9558010;9537294;8637446;7666783;7559145;8254785;8487676;1466870;3544454;6774694;395062;971771
P01229	16	19474795;17494102;17298549;9558010;9537294;8637446;7557841;7559145;8254785;8487676;1466870;3544454;3933451;6774694;395062;971771
P01233	8	12399442;11186912;10921230;8988397;2318574;395062;433028;971771
P01236	14	19474795;18440711;17494102;17298549;10418843;10341867;8637446;8963972;8988397;8487676;2510463;3933451;3842372;6819900
P01241	30	21062296;20444938;18092562;17376000;15058792;12519883;12373543;12124184;11026507;11010069;10052669;8988397;8532215;7666783;7557841;7658599;8175969;8005564;7965141;8427299;1466870;1635789;6293405;7347279;288855;838851;4719665;18092562;16554457;8427299
P01258	2	21243003;3931063
P01270	13	23558091;22710945;21632079;17457452;17376000;16217589;12145766;9482376;9427170;9228870;8786732;838851;3931063
P01275	4	16112402;12519883;3039255;1254731
P01282	10	22781650;18677446;18556138;11045565;10804204;7576312;3193505;3656550;2426476;3516303
P01286	1	7666783
P01303	12	22781650;18581269;16430653;15596391;14628554;11723173;11045565;10393880;9153661;7620299;7576312;3656550
P01308	1	4719665
P01344	3	22773197;11078681;9762866
P01350	4	3363465;3460562;6381185;4856812

P01375	187	23979726;23956505;23926264;23758598;23731227;23603407;23492769;23403365;23313320;23143993;23001399;22986158;22981449;22975435;22878925;22865690;22840733;22825006;22749984;22748825;22745823;22733360;22613732;22526707;22420033;22409448;22406975;22404382;22342994;22233298;22189457;22125095;22072681;21922518;21806470;21806469;21787762;21760535;21656712;21604270;21396163;21309692;21287269;21251802;21250919;21224756;21208533;21185611;21176673;21167907;21111721;20970752;20947810;20862369;20823594;20817464;20800648;20799028;20736048;20708606;20646530;20633123;20630763;20623539;20385073;20382225;20350367;20334467;20309734;20304963;20203181;20195207;20107429;20047904;19949368;19780197;19543752;19519665;19469692;19439611;19373550;19320007;19306380;19183262;19090911;18971481;18849175;18838947;18751914;18635178;18628687;18511041;18468795;18440605;18440241;18322000;18298463;18197140;17945432;17881483;17704735;17645692;17621255;17601981;17494103;17443214;17416966;17392476;17301687;17184185;17111361;17046032;16918386;16825529;16638021;16634039;16303916;16263098;16253423;16187294;16083358;16054120;16019083;15899263;15855648;15851082;15843065;15744602;15684771;15684769;15668227;15610171;15605375;15477364;15320513;15307904;15229242;15135227;15105666;15004554;14760011;14753477;14691582;14662726;14593216;14588118;14552870;14512160;13678663;12675974;12675973;12664616;12507778;12440358;12167759;12165135;12127673;12111861;11935370;11886441;11517251;11516566;11493024;11476601;11393259;11393256;11200245;11208917;11031095;10822448;10821736;10804204;10757326;10744154;10547095;10531440;10212323;9799824;9729336;9682014;9454848;9486767;9270046;9262171;8823387;7702707;17217414
P01574	4	23344866;20109445;19196180;17426631
P01579	12	23828573;23731227;23257628;22348141;21656712;20925481;20832407;15537875;15229242;14760011;14563689;11990353
P01583	5	22655536;22459192;22420033;9886082;10369555
P01584	108	17217414;10369555;23731227;23603407;23403365;23317037;23001399;22986158;22981449;22975435;22865690;22840733;22733360;22655536;22483094;22420033;22406975;22404382;22378878;22342994;22125095;22072681;21922518;21806470;21396163;21383505;21298060;21287269;21208533;21185611;21167907;21111721;20974246;20823594;20799028;20736048;20630763;20623539;20382225;20334467;20309734;20107429;20005255;19543752;19519665;19457130;19373550;19320007;19183262;18838947;18675261;18674548;18577426;18511041;18367607;18316064;18194440;17945432;17918744;17917587;17704735;17600517;17494103;17443214;17403033;17392476;17356380;17188663;17167171;17111361;16825529;16456668;16345062;16307587;16253423;16187294;16041804;16038625;16019083;15855648;15840993;15689362;15605375;15477364;15335103;15307904;15229242;15105666;14760011;14512160;13678663;12664616;12482119;12399442;12124194;12111861;11990353;11924887;11771937;11565605;11200253;11170729;10821736;9886082;9682014;9262171;9221936;8426182
P01588	53	23098731;22659566;22196867;22145921;22052241;22038355;22011644;21707531;21192293;21167907;21056627;20146558;20082560;20010384;19719963;19383246;19110203;19030901;18991489;18635178;18625498;18616414;18552697;18313052;18309506;18065151;17936749;17614942;17543134;17305256;17236773;17178192;17141961;17023852;16824697;16629619;16619678;16547753;16477096;16260722;15993112;15792521;15509883;15456912;15247477;12920606;12533271;12152030;12082184;11854521;8420523;2297306;6742994

P01589	3	9014956;8164533;1331272
P01857	11	21298060;665057;20401845;19200733;17905632;11393129;9576257;8259905;6719908;6505487;971771
P01859	11	21298060;665057;20401845;19200733;17905632;11393129;9576257;8259905;6719908;6505487;971771
P01876	3	10889567;6505487;971771
P01877	3	10889567;6505487;971771
P02144	3	22773196;21181573;10084442
P02452	6	23516080;22576416;12794745;12145766;11771937;6248997
P02458	1	23027386
P02462	1	23027386
P02511	1	23077034
P02649	7	21132529;18751914;18581664;18317192;16091415;15073526;12526030
P02671	24	1290164;23852558;22037947;17274489;12677772;12614584;10412796;7964707;8259328;1290164;1514887;1522997;1707599;2508383;3262334;3299130;3104622;3748594;2992116;4009754;3890800;853549;934690;9418975
P02675	23	23852558;22037947;17274489;12677772;12614584;10412796;7964707;8259328;1290164;1514887;1522997;1707599;2508383;3262334;3299130;3104622;3748594;2992116;4009754;3890800;853549;934690;9418975
P02679	1	9418975
P02686	73	23832758;23376368;22805224;22500090;22463720;22407783;22173726;22057439;22028197;21996274;21970342;21656231;21638341;21416627;20709122;20381564;20184788;19182380;19152029;19053058;18625299;18578635;18449589;17439350;17376002;17047682;16957585;16632872;16626970;16571744;16503802;16502219;16049170;16047549;16014718;15920160;15182305;15084655;14993064;14753480;14753473;14705147;14599661;14534158;14507981;12614587;12440382;12241646;11923434;11853017;11746773;11565606;11550218;11518733;11398181;11356861;11226756;11146478;10964948;10739580;10547103;10430053;9786985;9651218;9633812;9479054;9294414;7490015;7541476;8442406;1384082;3328836;2425054
P02741	21	23188848;22773196;22404382;19485254;19349048;19242618;18795484;18440241;18414426;18374004;18164328;15954057;15832900;15706560;15666138;15648796;12851776;9881738;8734896;2435822;414445
P02745	1	19770513
P02745, P0274	3	19770513;19091977;15684772
P02746	3	19770513;22884909;16345062
P02747	1	19770513
P02748	2	20952585;18578885
P02751	43	23320533;23289019;22022865;21396163;20645410;20606643;20347014;20206381;20167291;20053907;19144400;18373484;19845152;18083223;17597120;17439350;17438016;17314203;16500703;16287096;16237323;16102813;15665610;15592854;15530871;15341588;15145083;14962555;12895449;12794745;12526031;12099279;11890721;11755791;11358439;11352090;11179852;10412796;9417833;8834102;7964707;1514887;1471470
P02766	1	20141154

P02768	61	23233062;22046257;20401845;19349048;18774604;18627257;18617007;18187233;18077574;17711402;17402855;17387687;17236773;16806707;16671481;16550325;16179548;14753476;12920606;12507778;12149929;11935370;11476601;11050127;10914354;10640453;10412796;10213646;9292692;9270046;9261780;9403253;8786732;7741615;8413879;8371938;8366340;1471470;1984104;2297306;2818825;2930340;3346710;2439939;3104553;3668609;2430066;3525758;3698435;4056823;4068264;6526525;6206205;6411046;7139775;7136499;7130992;6165810;4901;1113150;5083939
P02775	1	1290164
P02776	1	1290164
P02778	7	21294159;20478301;17907930;16862543;15998793;15352216;14637115
P02787	8	12093138;10952914;10067862;10026110;7541476;7559145;2297306;2930340
P02790	1	23867460
P02794	5	19958190;12093138;8978469;8428551;6886688
P02795	5	22405890;19828842;19159657;17218363;15592854
P02818	14	23516080;22576416;22218778;21632079;18482879;17340221;16770357;14722626;12145766;9773450;9467550;8830990;7878394;1558739
P03372	16	23142039;22973889;21273111;17704735;12113672;10952914;10626954;10067862;10026110;9362426;9203052;22700771;20456002;20350367;17704735;16902996
P03951	2	7964707;3925930
P03956	1	11150324
P03973	2	20047904;17218363
P04035	1	22537532
P04040	16	19828842;19563509;19119913;18635178;16634331;16018585;15036352;14644473;12213642;11011974;9726263;9313901;8973665;7701506;2358556;2295968
P04054	1	19306380
P04070	10	16708170;15320513;15307903;11586111;11175376;10903607;10744154;9454848;9076865;3675178
P04085	17	23463486;22083254;18428026;7657809;22407783;22236767;21568693;20819515;19818206;19383401;17597120;14534257;10329979;9618703;8963994;7657809;8091423
P04141	11	23101949;22011644;20478301;19428687;19033079;17464087;17331604;16626091;15998231;15658127;15456912
P04150	12	19545280;19416634;19228956;18354017;16256102;14552870;11150324;10531440;7473799;8503522;1613805;1588607
P04156	1	20418657
P04179	7	21187959;19828842;16095570;15684769;14767765;12482119;10428065
P04234	7	23313320;22629434;21249281;18790013;18666440;18438914;17432962
P04271	40	23419261;22996688;22220508;21704617;21630007;21609310;21535942;20437933;20370565;20304889;20083105;20038240;19499171;19382871;19350385;19309047;19191513;19183262;18484102;18482974;18374350;18343116;18313337;17244329;16528168;16219025;16211443;16190875;15670387;15182944;15118880;15080887;14962555;14699277;11878171;9669763;10975736;8668354;8354567;1895117
P04275	6	22425718;7964707;1290164;1732896;1803505;2508383
P04278	2	20839151;17494102
P04406	5	21930674;20141154;17460353;15857397;9344569
P04553	4	12721213;10826266;7537955;7559145
P04626	3	23758598;20734425;15514921

P04628	12	24090150;23456240;23320533;23251385;22947224;22575440;22073235;22006831;19473059;18701700;21273111;19473059
P04629	26	23867460;23856436;23809594;23792206;23174180;21903010;21620945;21421025;20171177;20144886;19545610;18585435;18305238;15611995;12542855;12115676;11223160;11161589;11060810;10993692;9671662;9669765;9391013;8594213;8015371;1333605
P04637	11	23035104;22427977;21748659;20210855;17331604;12657369;11810634;11208917;10709875;5492504;23055316
P04731	2	17218363;15592854
P04733	1	17218363
P04792	9	22917619;21655070;21132399;20350367;19489718;15073526;14694501;22409448;22244304
P04839	5	22150233;21355819;19056384;18926823;17071951
P05019	46	18092562;16554457;8427299;23941795;22773197;22218778;22085254;21062296;20970752;20925481;20213470;19812967;19533653;19214561;18938163;18581269;18440711;18092562;18077567;17233289;16554457;16519668;16215319;16179548;16038877;15779090;15073526;14753473;12801087;12187277;11450028;11078681;11026506;10923678;10486180;9871451;9762866;9416300;7666783;7557841;7541476;8175969;8005564;22773197;11078681;9762866
P05023	1	22275761
P05067	19	23617630;22805224;22514792;21824499;21620594;20698757;19932144;18627258;18561916;17614942;16325784;12866814;11578629;11311801;10806498;9650755;9350423;8811125;7473801
P05089	9	22629434;22504113;22325098;22233298;21411654;19293775;18358516;17720160;17418108
P05107	12	11890721;10914354;22150233;22044160;21784069;21145887;19250688;15992367;15659600;15312174;15102919;15009633
P05112	8	23257628;22348141;22233298;21411654;20623539;14760011;12165135;10369555
P05121	3	12472582;9076865;7690062
P05129	13	21443453;20675200;20070863;19109493;19059398;18835424;18307037;17586495;17131417;15099403;10860578;10700012;10069504
P05155	1	18593519
P05164	85	23731227;23603407;22986158;22655536;22395142;22322369;22289688;22150233;22044160;21787762;21784069;21496122;21355819;21259324;21138398;21134362;21111721;20970752;20801040;20799028;20623539;20350367;20176685;20107429;19891733;19831872;19418318;19373550;19295488;19200733;19056384;19038604;19000748;18948848;18926823;18666440;18628687;18384773;18197140;18180375;17945432;17917587;17704735;17664140;17645692;17621255;17608147;17484972;17418876;17304106;17294055;17236773;17184185;17161287;17071951;16918386;16601144;16581192;16432531;16303916;15899263;15792522;15725342;15610928;15452194;15312174;15033786;15009633;14691582;12951232;12675974;11553680;11020993;10776915;9675307;9582256;9219976;9171180;8915909;8627308;8903674;8281452;1335518;1649310;2166776
P05186	50	23516080;21632079;21430166;20716462;18324828;18001205;16830131;16430372;16305315;16267833;15910779;15899260;15666138;15530870;14985870;14722626;14648541;14639555;12145766;11550223;10868785;9881730;9773450;9467550;9172248;8786732;8588079;8524605;8022633;7615177;1484734;1481247;1544955;1984104;2123534;2083875;2502349;2495875;2600593;3918147;6828228;6215609;6786254;6104224;215614;100499;831659;196527;811418;1090278

P05198	4	22815920;21933012;17885021;17578450
P05230	44	23562792;22975435;22124040;21663406;21557400;21486314;21411654;21096476;20488178;19738322;19141070;18482974;18312071;18291581;17717118;17418108;16823315;16487151;16099035;15804055;15247588;15055447;15036352;12427329;11939503;10833309;10380979;9749747;9486767;9417831;9418975;8662542;8594213;8015371;7682969;9418975;22649227;19955358;12557207;11320601;9418975;9192376;1588636;23979726
P05231	82	24042200;23731227;23727390;23639820;23399848;23143993;23001399;22878503;22715999;22578249;22504113;22420033;22406975;22404382;22369693;22333890;22233298;21922518;21788952;21760535;21617884;21604270;21294159;21167907;21134362;21092402;20823594;20736048;20623539;20478301;20382225;20304963;20038240;20029964;19831872;19543752;19519665;19257808;19138107;18511041;18446011;18440241;18390214;18316064;18088553;17720160;17704735;17600517;17597120;17494103;17392476;17167171;17111361;16417589;16345062;16184194;16129904;15954057;15851082;15840993;15477364;15128857;15105666;15048924;14760011;13678663;12442313;12111861;11924887;11723173;11453434;10821736;10757326;10712626;10369555;9799824;9682014;9262171;9014956;8571667;24042200;10712626
P05305	15	20945816;19537925;18308116;17259328;17122448;16187216;15636718;12668144;11402584;9555032;8864490;9094380;7473800;17893916;12668144
P05362	14	22289688;21599492;17167171;16187294;16155637;15767230;11563627;11200253;10776916;10098961;10094148;9014956;8627308;8928610
P05412	3	21671725;11150324;10343327
P05413	3	23011062;22773196;22110655
P05556	6	22150233;20213330;19929361;19038604;18926823;17161391
P05937	2	15312785;12803980
P05976	1	2293623
P06241	3	21167847;18680553;18680553
P06276	1	12149929
P06307	10	20950355;19619609;14559369;12688385;11723173;11457525;10097004;7576312;7912821;7795551
P06400	1	22510563
P06401	3	23542439;17618691;14622121
P06493	4	23360302;22848730;19672039;22848730
P06731	6	23971371;7478727;1469474;1895117;2230428;7212647
P06850	10	21492574;20470804;20458328;12399442;12114295;9558010;8988397;7576312;3925930;7133750
P06858	1	3548963
P06870	2	24128681;24128681

P06881	103	23413374;23068989;22829497;22140459;22041228;21939395;21806987;20970752;19817844;19660662;19372269;19247856;19235905;19146928;19059398;18973595;18712059;18338959;17885772;17577523;17339890;17314299;17046032;16860320;16839548;16540569;16099035;15998524;15929557;15890340;15804055;15755547;15715083;15680702;15652986;15233942;15082150;14552891;12895449;12402380;12125080;11738503;11686496;11476597;11398181;11168294;11161621;11045565;10714264;10460247;9670992;9486767;9417823;9344570;9344569;9116197;8974628;7576312;8201627;8405277;2682392;23867131;23068989;22655857;21939395;20970752;19660662;19372269;19247856;19235905;19146928;19059398;18824169;18712059;18338959;17719577;17577523;17540505;17503492;17331502;17314299;17046032;16839548;16540569;16305315;16099035;15665609;15082150;14637107;14637084;14552891;12811811;12402380;11168294;10960593;9486767;9344570;9116197;8875323;8201627;7795551;1667690;2682392
P07101	21	23520469;23106570;21830219;20668234;20573032;17885772;16487151;15715083;15067714;12619082;11829345;11398181;11260912;10915581;10906754;9520225;7859097;1316056;6741741;6123957;839258
P07195	14	23793797;23647384;21529317;19998478;19485254;12852839;12509801;11417439;9362426;8145266;1984104;2718797;6526578;6822753
P07196	141	16902996;23316955;23123586;23036616;23031841;23026410;22878503;22865656;22805224;22551686;22480691;22315999;22270191;22220714;22052241;21630429;21618230;21559863;21490212;21470565;21384547;21361731;21303267;21157914;21092402;20805831;20155820;19783284;19456214;19409869;19382871;19191513;19158290;18772508;18647603;18634003;18571867;18561916;19845152;18086148;17879538;17540505;17432961;16633897;16586436;16527274;16379576;16190875;16176808;16102815;15929557;15755556;15715083;15707984;15665609;15253805;15207354;15197746;15102919;15051157;14966352;14577863;14534158;12895449;12811811;12614588;12614587;12503060;12427329;12410380;11939503;11920709;11880833;11854516;11829345;11746773;11578629;11476597;11462799;11104506;10931587;10837800;10818764;10759184;10737623;10714264;10683270;10668431;10452368;10366029;10221783;9827690;9786407;9682827;9670992;9668671;9650755;9633812;9570810;9460769;9292692;9285520;9106471;10975736;8985951;8811121;8668354;7753403;8426193;1333605;16180033;1473395;1724469;1700924;3276818;3336206;3328836;3104553;3105816;6539814;7170062;713490;23916821;22815926;21618230;19103176;18990118;18508036;16447750;16305315;15869942;15854260;15673843;11226756;10683270;10383635;8873865;1700924;2258947;3819017;6539814

P07197	146	23316955;23123586;23036616;23031841;23026410;22878503;22865656;22805224;22551686;22480691;22315999;22270191;22220714;22052241;21630429;21618230;21559863;21490212;21470565;21384547;21361731;21303267;21157914;21092402;20805831;20155820;19783284;19456214;19409869;19382871;19191513;19158290;18772508;18647603;18634003;18571867;18561916;19845152;18086148;17879538;17540505;17432961;16633897;16586436;16527274;16379576;16190875;16176808;16102815;15929557;15755556;15715083;15707984;15665609;15253805;15207354;15197746;15102919;15051157;14966352;14577863;14534158;12895449;12811811;12614588;12614587;12503060;12427329;12410380;11939503;11920709;11880833;11854516;11829345;11746773;11578629;11476597;11462799;11104506;10931587;10837800;10818764;10759184;10737623;10714264;10683270;10668431;10452368;10366029;10221783;9827690;9786407;9682827;9670992;9668671;9650755;9633812;9570810;9460769;9292692;9285520;9106471;10975736;8985951;8811121;8668354;7753403;8426193;1333605;1618033;1473395;1724469;1700924;3276818;3336206;3328836;3104553;3105816;6539814;7170062;713490;23466931;21618230;21375803;19934591;18772508;16497507;23916821;22815926;21618230;19103176;18990118;18508036;16447750;16305315;15869942;15854260;15673843;11226756;10683270;10383635;8873865;1700924;225
P07204	5	23562508;15320513;15307903;11425904;10744154
P07225	3	15320513;9076865;7965142
P07355	2	21430360;15248295
P07357	2	20952585;18578885
P07384	2	23102374;19376093
P07550	10	19545280;11293827;9362426;22723636;17933964;14503640;6135730;21903880;15211988;7620299
P07858	4	21373949;15817261;14720218;2425054
P07949	6	21316153;16906542;16003691;14608602;10624807;906796
P07996	6	21168495;19780197;19719963;19342245;19278574;18612314
P08047	1	7615895
P08069	23	22644570;21273111;20970752;19533653;18581269;18440711;18092562;18077567;17233289;16554457;16519668;15779090;12850828;12801087;12187277;11450028;11026506;10486180;9871451;9416300;7541476;8175969;8005564
P08107	2	22409448;22244304
P08107	16	[illegible]26;17454396;17359722;16156717;15634402;11723173;11085891;11074053;7753505
P08123	6	23516080;22576416;12794745;12145766;11771937;6248997
P08138	29	23303920;23856436;23809594;23792206;23303920;21786560;21110803;20468053;20144886;20083190;19788572;19429149;18585435;18581269;17706365;17634369;17234430;16631308;15530871;15031718;12860969;12408842;11311801;11223160;11063050;10384258;9671662;8457883;15031718
P08151	2	21107202;12520472
P08172	1	22386753
P08183	1	22947335
P08185	1	20839151
P08195	3	19120441;11920709;9678508
P08235	1	19364523
P08237	2	9837860;132700
P08238	1	23487751

P08246	4	11553680;11350640;9770243;9675307
P08247	22	23678012;23249275;22815926;22052241;21476782;21424738;21249281;21145575;20132484;19961582;18802763;18352823;16497507;16237167;16100462;16022868;15602825;8848126;8615075;7735281;7859097;1316056
P08253	17	24436993;23499793;22805224;21455784;19675231;19631747;19074020;18298463;17005848;16707787;11414785;11388663;11095516;10716770;20943915;22798270;21756907
P08254	3	23926264;17184186;11095516
P08294	50	23523995;21111721;20817464;20364349;19875292;19563509;19473058;18407497;18289003;17486444;17184187;16689664;16634331;16332351;16187294;16095570;16018585;15864722;15792522;15684769;15610928;15207346;15036352;14767765;14666020;14644473;12657369;12213642;11977524;11853017;11453434;11200253;11032904;11011974;10815757;10437099;10322962;9726263;9680175;9313901;8973665;8915909;7478738;7701506;7841663;1414256;2058414;2244484;6181489;16095570
P08559	4	20438613;20392143;19119913;2352011
P08571	2	21599492;16206158
P08575	11	23466931;23313320;21740131;21599492;19077786;18438914;18088553;17432962;14570283;12794745;11045675
P08581	9	22140459;22079829;20860549;20709122;20468053;18480987;17884290;17549731;17425951
P08588	8	22723636;17933964;14503640;6135730;21903880;22275761;15211988;7620299
P08603	1	15785233
P08620	2	18581269;17207742
P08670	39	23632234;23272155;22978525;22865656;22791629;22161971;21956379;21883887;21250919;20645410;20155816;19257808;18930033;18374350;18316064;18065151;17671987;16902766;16344894;16294335;16240391;15335106;15207354;15115595;14610361;12900928;12861073;12655601;12619082;11746773;10683271;10225952;9872458;9417833;10975736;7541476;7540218;7955669;1895117
P08758	1	20438770
P08833	4	12611774;18938163;9762866;7541476
P08887	13	24042200;24042200;23639820;22369693;16129904;23639820;20478301;18446011;16184194;16129904;15128857;15048924;20478301
P08908	24	23360270;23221401;23099413;19840787;19260781;17881483;17214613;17204596;17047164;16914121;16836640;16739558;16488413;15872102;15649492;15380867;15321732;15275776;15152026;14622228;14575797;12764106;12610685;12560138
P08913	11	22081708;21222499;19000416;2059129;2058414;21047936;1675858;758380;22275761;15211988;7620299
P09038	77	22649227;19955358;12557207;11320601;9418975;9192376;1588636;23809528;23486600;23414684;23236888;23082997;22954116;22796886;22629425;22555431;22236767;22085254;22042562;21756547;21630429;22506349;20799884;20513364;20130176;20114065;19780197;19442692;18849003;18756526;18627027;18615534;18255230;18095482;17611275;17610915;17553847;17370779;17244329;17108169;16718733;16406666;16029658;16005441;15899248;15769312;15672632;12925000;12429182;12080821;11893024;11771937;11606629;11588623;10960593;10915567;10833309;10579585;10521141;10511239;10436058;10380979;10369555;9881728;9878195;9749747;9651218;9519278;9486767;8797678;8594213;7540200;8015371;7682969;23486600;23236888;23979726
P09104	17	23143000;22220308;21335942;20734425;20678995;19499171;18345116;17184187;16528168;16506604;15670387;15207346;12535938;12435422;11299626;1333942;7204643

P09172	11	16487151;10336136;10215931;9588600;9153661;7859097;1937684;7418270;938986;4430080;4784945
P09341	5	22798270;22655536;22404382;21604270;19932745
P09382	5	21188398;20091712;19941913;15893752;15464279
P09429	4	22158106;21343866;20195207;19090911
P09455	2	21196217;16688771
P09486	1	17611274
P09543	13	22952690;22500090;22174557;21336655;20374080;18008145;17061258;17044031;16447750;15845083;15378660;15006675;12431704
P09601	22	24112949;21657851;19589199;19119918;19035757;17912625;17259328;17047682;16156717;16095570;15319001;14753474;12678694;11990353;11723173;11450046;11208917;11085891;11074053;11026506;9622586;20945816
P09603	1	9682014
P09619	2	23236888;7657809
P09874	16	[illegible]90;18289003;17361386;16184194;15857303;15452194;13678663;12509801;11379817
P09917	8	23303944;22986158;22947335;18059327;16019083;2166776;3143469;3480080
P09919	21	23433334;23419550;23209732;23139012;22862301;22391867;22253813;22024901;21935680;21721873;21539498;20887150;20801040;20202082;19499175;18406145;17882016;17641973;17618991;17391650;16958589
P09960	1	19469692
P0C862	1	20107429
P0CG47	17	[illegible]04;11578629;10668768;10557125;9890271;9201240;8811125;8740714;8787148;1700934
P0CG48	17	[illegible]04;11578629;10668768;10557125;9890271;9201240;8811125;8740714;8787148;1700934
P0CG63	1	23626776
P10092	42	23867131;23068989;22655857;21939395;20970752;19660662;19372269;19247856;19235905;19146928;19059398;18824169;18712059;18338959;17719577;17577523;17540505;17503492;17331502;17314299;17046032;16839548;16540569;16305315;16099035;15665609;15082150;14637107;14637084;14552891;12811811;12402380;11168294;10960593;9486767;9344570;9116197;8875323;8201627;7795551;1667690;2682392
P10144	1	19170890
P10145	8	[illegible]62
P10147	8	[illegible]16
P10276	2	17239557;16420438
P10415	81	23731227;23632234;23492769;23344852;23274522;23180094;23174180;22982298;22981449;22733360;22453521;22342994;22295509;22289688;21787762;21748659;21496122;21439269;21394310;21111721;20933583;20830289;20816819;20799028;20708606;20385073;20370565;20176685;20107429;19891733;19817205;19815005;19716366;19545280;19525878;19373550;19183262;19120440;18948848;18838947;18785877;18665052;18628687;18322000;18197140;18180375;17945432;17892411;17704735;17664140;17645692;17621255;17418876;17392476;17331604;17304106;16601144;16303916;16191756;15896972;15715081;15668909;15307905;15115604;12853305;12823889;12680327;12657369;12482121;12125080;11402882;11398181;11105694;11007881;10709875;10658186;9800272;9530781;8786386;23274522;19525878
P10451	7	19813107;17392476;16345062;15592854;15528873;14513263;12820683

P10586	2	22836147;9076876
P10636	12	23499240;22514792;22052241;20717887;20038240;18680553;18307037;18219571;17233289;15715081;10759184;18680553
P10643	2	20952585;18578885
P10644	10	23603516;23177959;22342994;21671725;19293775;19222562;19141070;19138107;18656458;10804204
P10645	1	21249281
P10646	2	16139333;8619181
P10721	4	23608618;22295509;19859984;19182706
P10826	4	19800972;17239557;16984961;16688771
P10909	2	15785233;11702232
P10914	1	11723173
P10916	1	2293623
P11055	4	22644570;20213470;15725214;15453091
P11086	2	10906754;10363831
P11137	40	23419261;22815926;21704982;21658682;21375803;21355419;20707645;20698757;20155459;19925560;19476208;19462232;19318112;19106491;18992011;18672742;18584320;17706365;17701486;17397933;17115913;15790679;15522907;15077307;15051157;14570283;12614588;12429182;11563627;11200245;11045677;10912915;10776916;10759184;10737454;10712659;10412797;9326288;9350423;2809367
P11161	2	20083105;11746771
P11166	2	20590523;14515352
P11177	4	20438613;20392143;19119913;2352011
P11215	55	23526403;23313320;22815926;22629434;22425718;22329943;22041654;21704617;21336655;21142687;20427653;20374199;20155820;20083190;19946692;19780197;19332108;18849175;18617007;18455876;18438914;18353556;17882014;17671987;17506499;17432960;17329433;17244329;17184184;16978658;15995139;15739189;15737738;15635609;15610171;12794745;11912671;11890721;11588623;11146113;11114257;11064366;10998127;10914354;10195473;10098961;9795113;9670992;9582256;9418967;9285520;8989657;8981236;7629867;21970623
P11229	2	12605884;23184186
P11836	1	21249281
P11926	3	21411654;10320037;7577724
P12004	23	23602967;23526403;23180094;23135205;23093447;22510563;22484641;22246994;21710280;21229311;19376093;19205589;18008145;15528873;12666113;12578228;12499056;11585624;11077093;9507617;8963970;8524605;23526403

P12036	171	23316955;23123586;23036616;23031841;23026410;22878503;22865656;22805224;22551686;22480691;22315999;22270191;22220714;22052241;21630429;21618230;21559863;21490212;21470565;21384547;21361731;21303267;21157914;21092402;20805831;20155820;19783284;19456214;19409869;19382871;19191513;19158290;18772508;18647603;18634003;18571867;18561916;19845152;18086148;17879538;17540505;17432961;16633897;16586436;16527274;16379576;16190875;16176808;16102815;15929557;15755556;15715083;15707984;15665609;15253805;15207354;15197746;15102919;15051157;14966352;14577863;14534158;12895449;12811811;12614588;12614587;12503060;12427329;12410380;11939503;11920709;11880833;11854516;11829345;11746773;11578629;11476597;11462799;11104506;10931587;10837800;10818764;10759184;10737623;10714264;10683270;10668431;10452368;10366029;10221783;9827690;9786407;9682827;9670992;9668671;9650755;9633812;9570810;9460769;9292692;9285520;9106471;10975736;8985951;8811121;8668354;7753403;8426193;1333605;1618033;1473395;1724469;1700924;3276818;3336206;3328836;3104553;3105816;6539814;7170062;713490;23916821;22815926;21618230;19103176;18990118;18508036;16447750;16305315;15869942;15854260;15673843;11226756;10683270;10383635;8873865;1700924;2258947;3819017;6539814;23793903;23614684;23419261;22848005;22270191;21658682;21618230;21337374;21258860;21251802;20805831;20698757;20678995;20447345;20381564;19533335;19462232;19458542;19413502;18772508;18578635;18484898;17703359;16190875;16176808;16099035;15576480;
P12235	1	15573402
P12259	2	19853891;15320513
P12272	4	15577056;12201902;11035476;10578246
P12277	17	22773196;21303658;19363309;14997963;14969411;14639535;12214901;11990354;10622457;9848487;1955370;1819975;2718797;3165203;7271386;6071347;14167093
P12314	1	19770513
P12643	12	22204020;22204004;21952743;21925422;21518886;21242869;19914878;18037352;21508230;23349763;20005873;20850923
P12644	7	21508230;23349763;20005873;23635322;21918888;21407803;20005873
P12821	1	15889697
P12830	2	21679705;21316153
P12882	5	24022865;16931550;11138583;10567091;8596693
P12931	7	23867460;21573723;18307037;16906542;15307904;14753479;11563494
P13236	1	22798270
P13497	1	20850923
P13498	2	22884909;16345062
P13500	25	23477737;23028463;22655536;22420033;21287269;21265596;21092402;20623539;20478301;20038240;19932745;19257808;18338959;17704735;17167171;16042867;15998793;15689362;15635609;15135227;12111830;11398181;8928610;20155816;18338959
P13501	6	22420033;21529317;20478301;18612314;16014718;9698165
P13591	16	23419261;23186720;21630007;21316153;20381564;19443935;19170182;18820404;17626035;17014846;16860320;16443221;16087243;11212863;9671667;8164533
P13611	8	19604403;17141961;14561854;12893430;12520031;12440375;11923444;10483914
P13612	6	22150233;20213330;19929361;19038604;18926823;23477737
P13671	2	20952585;18578885
P13726	1	8619181
P13945	2	21903880;22275187

P13987	1	20952585
P14136	302	23916821;23831998;23793903;23702616;23526403;23523995;23518227;23441695;23419261;23399872;23386122;23322541;23289019;23272155;23180094;23151374;23027386;23001399;22999930;22951907;22900481;22865681;22824304;22815926;22791629;22733360;22730180;22722907;22683506;22510563;22500090;22484641;22407596;22329943;22244304;22220714;22213649;22193443;22161971;22041654;22038545;22020092;21970623;21970342;21956379;21952042;21931744;21854445;21807380;21704617;21671795;21647706;21620945;21609310;21574060;21488085;21402118;21375803;21355419;21337374;21336655;21334381;21316362;21284970;21264949;21258860;21251802;21250919;21222572;21046809;20887664;20734425;20711818;20707645;20702718;20619335;20590523;20399758;20381564;20375134;20370565;20307513;20226782;20202079;20184788;20170651;20155459;20151365;20130193;20109536;20083105;20038240;20005873;19934591;19815010;19782669;19533524;19528093;19494564;19445935;19418545;19383087;19350385;19332108;19330845;19196180;19191513;19182380;19165795;19069631;18992013;18971163;18722369;18721107;18672742;18649176;18618667;18617007;18614693;18484898;18482974;18406145;18373484;18358622;18353556;18316064;18291533;18213442;18194440;18065151;18008145;18001275;17917587;17907381;17671987;17611275;17601981;17506499;17503492;17482149;17438016;17432961;17425951;17376003;17329414;17244329;17074445;17044031;16902766;16850748;16808875;16806707;16774471;16689668;16671481;16633897;16626970;16586436;16447750;16406666;16294335;16262629;16240391;16219025;16190875;16172374;16100462;16099038;16087243;16083359;16051494;16041804;16038450;16010451;16004983;15998793;15892602;15892130;15866044;15857397;15769312;15739189;15736057;15680701;15672632;15668227;15605375;15530871;15495269;15335106;15319002;15307906;15207354;15197746;15129757;15115596;15115595;15051157;15006675;14999065;14993064;14991839;14981254;14962555;14769386;14705147;14651809;14644473;14610361;14577863;14570283;14534257;14534158;12900928;12895450;12861073;12821387;12797382;12673833;12655601;12619082;12578228;12499056;12379246;121515
P14151	1	21142687
P14174	8	23618747;23140983;22833439;22613732;19123236;16460709;13067333;9378439
P14209	3	23632234;21249281;18054697
P14210	10	22140459;22079829;21903726;20860549;20468053;18480987;17884290;17600518;17549731;17425951
P14317	26	23809594;23792206;23303920;21630007;21337374;20083105;19429149;18373484;17706365;17503492;16691121;16305316;16102813;15530871;14593216;14552870;12440358;12408842;11331375;11311801;11223160;10993692;10384258;9671662;9669765;9391013
P14416	1	12946566
P14555	13	22029359;18751914;22581384;21142140;20127525;18755070;17917587;16566026;16498630;15248295;11499402;8522975;7473799
P14625	1	21807380
P14672	3	22865656;15107410;9837860
P14778	4	19932745;19932745;17382514;11565605

P14780	41	20943915;22798270;24436993;23926264;23867460;23731227;22853439;22805224;22798270;22655536;22409448;22049432;21784069;21756907;21657851;21455784;21287269;21248119;21176673;20965533;20947810;20862369;20382225;20029964;19780197;19675231;19074020;18384773;18352832;18298463;17217414;17071951;16454634;12956462;12196576;11414785;11388663;11150324;11095516;10716770;21756907
P14784	3	9014956;8164533;1331272
P14867	1	17115913
P14902	2	23415704;8149236
P15018	14	22715999;22085254;18673204;17111361;16957585;16563523;15779090;15537875;15246839;14651808;12589929;10712626;10212315;10712626
P15104	3	23525248;17893916;10962139
P15173	7	22644570;20213470;19214561;18236467;16184607;12611774;9755066
P15309	1	11193545
P15336	12	23583688;21970623;21671725;20552220;19293775;18785877;18672032;17719180;16478624;15896906;14662791;12721213
P15559	8	22853439;19589199;17618976;17584182;11532430;10686069;8710182;8848126
P15692	58	23562792;23486600;23236888;23116416;23064509;22792773;22683506;22453521;22450230;22186804;22145921;22085254;22023610;21818951;21735784;21630429;21568693;21396163;21299336;22506349;21192293;21092735;20860549;20799882;20701430;20698758;20468053;20334467;20010384;19879362;19442162;19319198;19257807;19226205;18990142;18773817;18599078;18367307;17666954;17633488;17600519;17420770;17409380;17229351;16284571;15319002;15307904;15191799;15161688;14753479;14584823;12927201;12134907;11029637;10199251;9517460;23486600;23236888
P15863	1	9171182
P16035	1	10716770
P16070	2	21599492;15308314
P16104	1	21748659
P16109	2	11563627;10094148
P16112	8	23027386;23001399;21952042;20334823;19675231;18314199;16987227;11425904
P16220	16	23583688;22981449;22911773;22384207;21970623;19293775;18785877;18672032;17720160;17719180;16478624;15922485;15896906;15869943;15611995;15135227
P16234	4	22629425;20130176;16691121;7657809
P16278	18	20170651;18343575;17885772;16289170;15716305;15115607;12904465;12482121;12203389;12115695;11606629;11550223;11424189;10933968;10023440;9651218;9417824;9369296
P16284	3	22336872;21630007;20153400
P16298	8	23981724;18577426;14767765;12429226;11060810;11007881;9878202;8791233
P16389	4	22407783;21110920;14984408;10712629
P16422	1	20875224
P16885	1	20070863
P17302	12	9183689;23403365;22975435;22951907;22469052;22399755;18649176;18617007;15977163;10751674;9183689;22951907
P17405	2	18838947;18751914
P17538	1	19442692
P17600	12	23859181;22579680;22384207;21316361;20384775;20132484;19138107;18672032;17719180;17083989;15869943;21145575

P17612	10	23603516;23177959;22342994;21671725;19293775;19222562;19141070;19138107;18656458;10804204
P17655	1	10878587
P17661	1	16931550
P17676	6	24042200;24042200;23639820;22369693;16129904;22578249
P17677	90	23174180;22827732;22805224;22542946;22504113;22426865;22193443;21884698;21833549;21630006;21611127;21402118;21316153;21308793;21046809;20513364;20381564;19761692;19757023;19679168;19559075;18647603;18602393;18482724;17928316;17885772;17879538;17503492;17466943;17447249;17382306;17331502;17131417;16965762;16897366;16862564;16839548;16460709;16367770;16367769;16240391;16038451;16022864;15862959;15716400;15665609;15522871;15350649;15296835;15128857;15004554;14966352;14637107;14637084;14598294;14552895;14520670;12578228;12440381;12151536;12125080;11939502;11920709;11880833;11867737;11867727;11813238;11776060;11773606;11398181;11154843;10785453;10762348;10632033;10383635;10343327;10336136;9878202;9827690;9671667;9453362;9391013;9300440;8811121;7478195;8405276;7694445;8426193;1421106;1333987
P17813	2	20153400;19859984
P17861	3	22337234;17578450;16156717
P17927	3	19736560;17882014;8989657
P17931	6	22884909;22510563;21394541;21187959;17460353;16345062
P17936	3	18938163;9762866;7541476
P17948	5	19319198;18773817;17409380;12927201;11029637
P18065	4	12611774;18938163;9762866;7541476
P18075	6	23349763;20005873;19765637;15648662;11720729;10699444
P18405	1	23542439
P18507	14	19358834;23542439;23332495;22449374;20203195;19358834;19225548;18845615;18206170;17376001;17046753;15157699;12618352;18495826
P18509	10	22674051;21187959;18677446;18410926;16888234;16198696;15913892;15698618;10804204;9582259
P18510	8	[illegible]
P18847	3	19853381;17284176;16420436
P18848	3	22873727;22337234;21933012
P18850	3	22873727;21933012;21638341
P19174	3	12098653;9840766;20070863
P19235	10	21167907;21056627;19110203;17614942;16619678;16260722;15792521;15456912;15247477;11854521
P19320	4	17073815;11031083;8928610;23477737
P19438	19	22840455;21309692;20309734;19949368;19469692;16083358;14552870;11517251;22840455;22484641;21185611;20107429;18322000;17621255;17443214;16629620;16083358;12111861;11517251
P19440	1	8732872
P19793	4	17239557;16420438;17201484;16420438

P19838	65	23880092;23574812;23553703;23499960;23492769;23361876;23359120;22981449;22853439;22798270;22733360;22578249;22484641;22450230;22324804;22289688;22125095;22038545;21889575;21784069;21510818;21383505;20954833;20868716;20862369;20830289;20799028;20708606;20646530;20370565;20176685;20091771;20047904;19891733;19716366;19522780;19418318;19416634;19358984;19295488;19056384;18948848;18665052;18628687;18197140;18180375;17918744;17664140;16776360;16253423;16179517;15998793;15922485;15684769;15478370;15452194;15229242;15135227;11517251;11393255;11150324;10428065;9729336;9547234;17918744
P19875	3	19932745;19257808;12111830
P20020	1	21303658
P20023	3	17882014;8989657;16936276
P20042	1	17578450
P20309	4	22386753;23184186;10069506;7742851
P20333	16	22840435;22484641;21183611;20107429;18322000;17621255;17443214;16629620;16083358;12111861;11517251;19949368;19469692;16083358;14552870;11517251
P20366	36	23564154;22825006;22781650;21671725;20096457;17924052;17314299;17251415;16918386;16680762;15998524;15807261;15253807;15135936;15082150;12895449;12402380;11427303;11342967;10651897;10393880;10336136;10085363;9670992;9153661;9416325;8988397;7543799;7795551;7691648;1284659;1701525;3401725;2426476;2410810;6181489
P20396	54	18980474;15993112;15665420;11586111;10499720;8710051;8685080;7643599;7473798;7768282;7659833;7543799;8487676;8432363;8278676;1636062;1570411;1813666;1797554;1850699;2123006;2120495;2112263;2508415;2499400;2497681;2497678;3132308;2830771;3127616;3116691;3124256;3128405;3127060;3106200;3090448;3010180;2870700;3945478;3842372;3927795;2410810;3917942;3004747;6435011;6433052;6097276;6657511;6410971;6306678;6811967;6799827;6792542;6116193
P20472	3	22000862;21930674;21445247
P20701	2	9031328;8928610

P20783	183	23792206;23746279;23257628;23131414;23066785;22993437;22875000;22573254;22244304;22211901;22159095;22137336;22130565;22042649;21996274;21995852;21783247;21470565;21441969;21420392;21384547;21375803;21243429;21135740;21105148;21083432;20846445;20816761;20660255;20619785;20597688;20399758;20350361;20005688;19812967;19884507;19874785;19818206;19784741;19648914;19603426;19513915;19442692;19383401;19240691;19203225;19054721;18672742;18585435;18369383;18338331;18253945;18191837;18159998;18077567;17881483;17719577;17708337;17671987;17540369;17522325;17459471;17439351;17376002;17316612;17221884;17112518;17072444;17055159;17014846;16988042;16928183;16919351;16797770;16786574;16773039;16764857;16718733;16689666;16632552;16430373;16387298;16289170;16102815;16099035;16049170;16022868;15869943;15858411;15716305;15652986;15649491;15262419;15254096;15128857;15114617;14637085;14637084;14515351;12904465;12895449;12781986;12774239;12710933;12676157;12598631;12589929;12435430;12353221;12173755;12099279;12009779;11810020;11755791;11550223;11377851;11358454;11241740;11200253;11186234;11168530;11114257;11104504;11077420;10993692;10757326;10712659;10683293;10583476;10581084;10448414;10212315;9840350;9822165;9743566;9699155;9651218;9486767;9417827;9391013;9204937;9000447;8594213;7561352;7841375;8114912;8298102;1333605;23360280;23124646;23098734;21430166;21421025;20819171;20600315;20554000;20171177;19118106;18095482;17686909;17584113;16629625;16305315;14699980;12589927;12440384;12440372;12440358;11923434;10811391;10384258;9932426;9369296;9199395;8993706;15901762;15748877;15144859;15032708;10763505;1072
P20800	3	17893916;12668144;12668144
P20809	1	22715999
P20827	2	21603973;21603973
P20916	48	23592243;22350947;22133879;21784510;21699896;21596039;21110803;21087927;20702718;20547125;20534525;20484625;19800972;19757023;19619659;19337830;19158290;18930141;18820405;18701700;17692017;17623024;17362886;17072444;17011208;15866044;15634734;15548666;15525355;15504325;15128857;15073526;15031718;12812757;12764110;12718855;12658441;12440377;12165135;11752461;11358445;10743699;10483914;9486767;9055294;8950700;8085934;20702718
P21145	1	23196718
P21217	1	19476208
P21246	1	15350649
P21291	1	22288476
P21453	2	22417787;16990586
P21554	21	23152849;23108547;22791629;21813113;21265596;20920894;20156559;20035773;20214038;18930143;17045264;16360182;12823482;11249966;10860578;10357256;21813113;20035773;19533526;17045264;11249966
P21579	1	24041988
P21583	2	20887150;19182706
P21589	2	23477737;6317425
P21728	11	23775067;22510563;19204052;15664589;12946566;12666113;12506309;11893891;1726151;19204052;18480366
P21730	2	23855891;23033813
P21741	1	21196217
P21817	2	15032708;11939501

P21860	1	23758598
P21917	1	11979329
P21918	2	19204052;18480366
P21980	3	22042649;12065630;11402370
P22004	1	21196217
P22083	2	22791629;20875224
P22301	28	23828573;23731227;23140983;23028463;22875000;22825006;22593113;22205935;21760535;21411654;21111721;20863526;20623539;20334467;19716366;19183262;16253423;15892959;15453992;14760011;14622710;14588118;14563689;12165135;12042099;11170729;10547095;10506519
P22303	20	23386718;23184186;17439352;16332351;12149929;11873957;11045565;10895495;10797957;9685649;9587481;9165429;9062807;8861623;7939462;8450950;1452079;1506033;339874;1239682
P22352	16	[illegible]44;16634331;16018585;15864722;15610928;15036352;14644473;11011974;7701[illegible]
P22459	1	23896350
P22466	5	16198696;15476702;15236239;11045565;7576312
P22694	10	23603516;23177959;22342994;21671725;19293775;19222562;19141070;19138107;18656458;10804204
P22736	1	17201484
P22894	1	22687332
P22897	3	23775900;22233298;21656712
P23219	21	17184185;15885321;11063829;9886082;23901068;21933591;21781963;17184185;15319002;15293235;15141096;11107569;11063829;11032961;10393880;9872457;9000453;8657340;1588635;3143469;3418749
P23229	1	17161391
P23297	1	9669763
P23415	7	23248270;21775715;20733588;20190766;17046753;12957506;12618352
P23443	6	18236467;17885021;20678995;20678995;20678995;17885021
P23458	7	21833847;16417589;17131417;16957585;16890196;22715999;20161735
P23515	23	23642707;22350947;21699896;21596039;21087927;20547125;20484625;19757023;19672953;19619659;19580419;18930141;18820405;18692574;17692017;17630211;17011208;15866044;15548666;15504325;15128857;12764110;12658441
P23528	2	22110655;19138107

P23560	294	24490534;23914898;23884949;23731762;23583688;23581453;23349859;23344852;23257628;23182350;23174180;23131414;23036616;23022460;22982152;22981449;22959240;22952613;22917619;22911773;22801282;22699902;22683506;22650045;22650042;22650041;22650040;22650038;22650037;22648027;22579680;22573254;22531936;22515203;22453521;22450230;22423083;22384207;22348141;22326790;22266141;22244304;22211901;22178331;22145921;22130565;22027236;21888482;21752551;21559863;21476782;21430166;21411654;21358740;21310212;21308793;21283639;21209491;21195072;21083432;20933583;20869112;20851742;20846445;20839450;20838921;20719091;20716462;20660255;20444938;20354783;20226152;20209974;20190766;20190624;20144886;20083190;20079352;20029964;19961582;19812967;19940189;19923273;19908250;19845866;19788572;19784741;19768787;19756447;19703592;19626995;19545610;19530162;19489718;19476208;19462232;19428687;22009833;19344636;19318112;19290803;19222991;19203225;19182380;19170182;19143246;19138107;19069631;19054721;18973595;18760313;18672032;18672025;18585708;18585435;18484898;18440711;18438913;18320028;18305238;18077567;18001204;17928316;17913768;17898225;17885772;17881483;17719180;17671987;17522325;17459471;17439352;17439351;17376002;17376001;17221884;17083989;17055159;16938843;16924202;16909619;16786574;16718733;16689666;16671480;16632872;16632552;16631308;16462594;16460709;16367769;16288464;16179548;16102815;16099038;16099032;16049170;16022868;16010451;15901762;15899240;15869943;15862959;15858411;15707984;15665609;15665420;15649491;15639807;15485774;15464279;15296835;15236239;15193526;15129166;15115607;15099674;15069022;15006710;14966352;14753473;14697859;14694501;14637085;14637084;14598294;14515351;14512160;12904465;12895449;12821376;12781049;12774239;12676157;12658441;12429228;12353221;12189689;12173755;12165137;12099279;12009779;11867727;11853017;11852178;11769614;11585248;11573989;11532245;11450046;11450028;11430880;11421586;11331375;11358454;11284549;11241740;11186234;11168530;11161589;11114257;11104504;11077420;11026506;109936
P23582	1	17061258
P23759	3	22644570;20213470;16447751
P23760	1	21710609
P23975	1	16143650
P24071	1	23299262
P24385	3	23775067;22510563;12666113
P24394	1	20623539
P24821	9	22473292;21443931;20606643;15892123;15592854;15341588;12526031;11085900;9285520
P24941	1	21710280
P25024	3	20877331;17601981;17601981
P25025	3	17601981;20877331;17601981
P25063	3	20806064;19962782;19458237
P25098	1	23359120
P25100	2	23583569;23583569
P25103	9	23564154;18513129;17559837;17314299;15922485;15135936;15082150;8207481;20096457
P25105	2	8758444;8762548
P25116	5	23832758;23647384;15307903;11186232;23832758

P25391	52	22993437;22236767;21783247;21092402;20818775;20799884;20643907;20206381;20053907;19768787;19196078;19163037;19144400;18786615;18647603;18385339;18373484;19845152;18083223;17611274;17597120;17570475;17503492;17466943;17314203;16987227;16774471;16500703;16287096;16102813;15174067;14561854;12826801;12592689;12589929;12440376;11920709;11771939;11771937;11356383;10630189;10586102;9878200;9486767;9417833;9301195;8981236;7753403;7859097;8457890;1690226;3053227
P25445	26	23487751;23196718;22260324;22038545;21181266;19469692;19120440;19006686;17703359;17518537;17357496;16689665;16629620;16202410;15567326;15223929;15004554;11311801;11302628;11208917;10912923;22038545;21181266;17518537;10912923;23935974
P25713	2	21815784;21294159
P25874	1	22865656
P25942	2	19469692;15447673
P26367	3	22473852;22469052;19955358
P26441	32	23635322;23267738;22085254;21918888;21407803;21157914;20181596;19626993;18803859;18615534;18581269;17610915;17044031;16005441;14715147;12589929;11331375;11358454;11241740;10923678;10683293;10579585;10486182;10369555;10212315;9878195;9835219;9651218;9417827;9058319;9000447;8457883
P26447	1	15350649
P27169	2	12897486;12382130
P27348	1	23177959
P27361	43	15668227;22865681;19109493;24042200;23832758;23647384;22515203;22027236;21355419;20933583;20830289;20707645;20067580;19672039;19469685;19090911;18948848;18180375;17329433;16978658;16888810;16513110;16478624;16460709;15922485;15668227;15229242;23361876;22733360;22634758;21310020;20536940;20444938;20381564;19383246;19196180;19090911;18630599;17329433;15707984;11031088;19855390;23647384
P27695	1	12563220
P27815	5	23028463;22865690;21355819;18455876;23028463
P27930	3	19932745;17382514;11565605
P27986	15	22930444;21720761;21496122;20132484;20070863;19800972;18785877;17611275;17457363;16460709;15668227;20585375;18785877;17703359;17457363
P28221	3	23360270;15152026;23221401
P28222	5	23360270;21653728;15152026;23221401;22227062
P28223	35	22227062;23360270;23337537;23248270;23221402;22227062;22056918;21272578;21211552;20698759;20085755;17059818;16760346;15772352;15716627;11717232;11358440;23337537;23159275;21036396;12426040;23481546;21653706;20861436;16707714;15892603;15380867;15275776;14622228;12193190;10407057;7957735;7964712;7683713;1830095
P28329	29	23859181;23500094;23184186;22579680;22445934;20719091;20698759;20384775;19318112;18253945;17084537;16774476;16632872;16487151;15862959;15476702;11939502;11776060;11398181;11391643;10384258;10025688;9749747;9392042;8931012;9165429;7885718;1933308;3944881

P28335	33	22227062;23337537;23159275;21036396;12426040;23481546;21653706;20861436;16707714;15892603;15380867;15275776;14622228;12193190;10407057;7957735;7964712;7683713;1830095;23360270;23337537;23159333;22993431;20980537;20512126;19840787;18045717;16914121;16760346;15649492;11717232;11358463;11358440
P28482	48	15668227;22865681;24042200;23832758;23647384;22515203;22027236;21355419;20933583;20830289;20707645;20067580;19672039;19469685;19090911;18948848;18180375;17329433;16978658;16888810;16513110;16478624;16460709;15922485;15668227;15229242;23361876;22733360;22634758;21310020;20536940;20444938;20381564;19383246;19196180;19090911;18630599;17329433;15707984;11031088;22027236;20943915;20849836;20067580;18180375;17329433;19855390;23647384
P28562	2	19383246;18630599
P28702	4	17201484;16420438;17239557;16420438
P28799	2	20012633;19946692
P28906	19	23632234;23627822;22145921;20374199;20029964;19309047;18583926;19364066;18088553;17666954;17618991;17610376;16929981;15790679;15129757;15052772;12794745;11424189;16929981
P28907	1	16929981
P29017	1	21599492
P29274	21	21486435;20385073;19407255;19295488;18305238;17853654;17177231;16777350;16506468;16488692;15589516;17559837;17177231;14992338;12596519;12207572;11320597;11170727;10784116;9842828;9535120
P29279	1	15796357
P29320	4	16083359;12075988;20949525;12075988
P29371	3	20096457;17907839;8207481
P29373	1	16688771
P29459	4	22420033;18438913;18322000;14760011
P29460	4	22420033;18438913;18322000;14760011
P29466	6	22733360;22378878;18367607;13678663;12837623;10938439
P29474	134	23135205;21496122;20141964;18400399;18096601;17287083;16786429;16776360;16179549;15895828;15036352;14644473;12232549;11835747;11834310;10455303;8903674;7530816;17618976;15895828;11834310;10921077;23731227;23700789;23688865;23659940;23135205;22748825;22125095;22002544;22000862;21626169;21596101;20633123;20468054;20382225;19741488;19675280;19651280;19418456;19291395;19056384;19026989;18855939;18096601;17712576;17645692;17618976;17484972;17385265;17353913;17287083;17258865;17217414;17017921;16978658;16791086;16786429;16703907;16671477;16246329;16179549;16100462;15895828;15640758;15486490;15353953;15335103;15229242;15036611;15036352;14993064;14767765;14753478;14753477;14666020;14644473;12903514;12865221;12810381;12713641;12675973;12490011;12402380;12232549;12133546;12042098;11844273;11835747;11835739;11834310;11453434;11427303;11425438;11424948;11253169;11200245;11191842;11146113;11068337;11043549;11031095;10984543;10928969;10925216;10923399;10790883;10697245;10686069;10486182;10471208;10455303;10393880;9871452;9871451;9848481;9795113;9760142;9507944;10921077;9416300;9208207;8880855;8863192;8791233;8710182;8861107;8903674;7478189;7753574;7525335;7530816;7687046;7684000

P29475	160	17618976;15895828;11834310;10921077;23135205;22000862;21626169;21596101;21145575;20633123;19931630;19291395;18855939;18556138;18219571;18074109;18053028;17500094;17454396;17287083;17258865;16786429;16776360;16774476;16671477;16246329;16198696;16179549;16095570;15895828;15036352;14753477;14644473;12713641;12490011;11835747;11200245;11045565;10686069;9871452;9871451;9416300;9208207;8880855;8861107;7525335;7530816;21145575;23731227;23700789;23688865;23659940;23135205;22748825;22125095;22002544;22000862;21626169;21596101;20633123;20468054;20382225;19741488;19675280;19651280;19418456;19291395;19056384;19026989;18855939;18096601;17712576;17645692;17618976;17484972;17385265;17353913;17287083;17258865;17217414;17017921;16978658;16791086;16786429;16703907;16671477;16246329;16179549;16100462;15895828;15640758;15486490;15353953;15335103;15229242;15036611;15036352;14993064;14767765;14753478;14753477;14666020;14644473;12903514;12865221;12810381;12713641;12675973;12490011;12402380;12232549;12133546;12042098;11844273;11835747;11835739;11834310;11453434;11427303;11425438;11424948;11253169;11200245;11191842;11146113;11068337;11043549;11031095;10984543;10928969;10925216;10923399;10790883;10697245;10686069;10486182;10471208;10455303;10393880;9871452;9871451;9848481;9795113;9760142;9507944;10921077;9416300;9208207;8880855;8863192;8791233;8710182;8861107;8903674;7478189;7753574;7525335;7530816
P29803	4	20438613;20392143;19119913;2352011
P29972	1	21092735
P30041	2	21187959;21187959
P30043	15	23579633;21376061;17584182;15672686;15488309;14993064;11532430;10925216;10790883;10707844;10686069;10471208;9836263;9760142;8710182
P30405	1	15573402
P30411	2	18255230;22862305
P30519	3	20945816;14753474;11450046
P30531	1	20059302
P30542	22	20065990;19651244;18289533;16572566;15893424;15589516;14992338;12821393;12426040;12207572;11320597;9535120;17559837;17177231;14992338;12596519;12207572;11320597;11170727;10784116;9842828;9535120
P30556	1	23174180
P30926	8	18855939;16888810;16723220;15716630;15694269;15135227;11334389;956661[illegible]
P30939	1	21653728
P30990	1	2426476
P31152	15	22865681;23361876;22733360;22634758;21310020;20536940;20444938;20381564;19383246;19196180;19090911;18630599;17329433;15707984;11031088
P31371	1	10833309
P31645	7	22993431;21211552;19840787;18825396;16806182;12061865;11532245
P31749	32	20585375;18785877;17703359;17457363;24042200;20678995;19943951;22253859;20678995;22982298;22865656;22409448;22253859;21970623;21496122;20678995;20585375;19533653;19176818;19109493;18785877;18768685;18305238;18236467;17885021;17553847;17457363;17233289;16957585;16511963;15896972;20678995
P31751	24	22253859;20678995;22982298;22865656;22409448;22253859;21970623;21496122;20678995;20585375;19533653;19176818;19109493;18785877;18768685;18305238;18236467;17885021;17553847;17457363;17233289;16957585;16511963;15896972
P31946	1	23177959

P31949	1	21655070
P31994	1	22233298
P32004	23	23049984;22815920;22497349;22431726;21671795;21445247;21337374;19909742;19861493;19458237;19170182;19059398;17611275;17438016;16860320;16630048;16083356;14577865;14561854;12115695;11606629;10998106;10495438
P32119	1	16586436
P32239	3	19619609;11457525;10097004
P32245	9	17627526;16550325;16179548;15662938;14753476;10719082;10391370;10225218;8971834
P32246	4	20155816;21332278;21265596;12111830
P32297	8	18855939;16888810;16723220;15716630;15694269;15135227;11554389;956661[illegible]
P32754	6	20675200;18835424;11438395;11166985;9674552;3702563
P33151	1	19859984
P33681	1	21599492
P33764	1	21539830
P33993	2	23526403;23526403
P34059	138	24478364;23879089;23839053;23804083;23790207;23731762;23596678;23531013;23520469;23186720;23098734;23061434;23022460;22934782;22917776;22836147;22827732;22728374;22650045;22650041;22650038;22650037;22629425;22579680;22497349;22465128;22420304;22193443;22159095;22114278;22042649;22016526;21946272;21885201;21855577;21848846;21753849;21697383;21630006;21596037;21574751;21490212;21310212;21215745;21046809;20855243;20846445;20839450;20673791;20620201;20620180;20599980;20558254;20552220;20536940;20534825;20419536;20354783;20179269;20134188;20130176;20087118;20079803;19940184;19935638;19884507;19788572;19768787;19761692;19675231;19668200;19659409;19545610;19255587;19192870;19143246;19109493;19005065;18930033;18795474;18486695;18373483;18353313;18320028;18314199;18221191;18158149;18001203;18000864;17970629;17936753;17913768;17719577;17540369;17495772;17439352;17329414;17099112;17083989;17050723;16965762;16924202;16837588;16705682;16632552;16624960;16367770;15716629;15689553;15514921;15307905;14960611;12821386;12821376;12781986;12617951;12440375;12435430;12115676;12009528;11948352;11769614;11450046;11450028;11186234;11026506;10790883;10630190;10486182;10212315;9878200;9871451;9840350;9699155;9772453;8594213;1333605;19940184
P34130	48	23360280;23124646;23098734;21430166;21421025;20819171;20600315;20554000;20171177;19118106;18095482;17686909;17584113;16629625;16305315;14699980;12589927;12440384;12440372;12440358;11923434;10811391;10384258;9932426;9369296;9199395;8993706;22244304;21083432;17522325;15193526;12435430;12189689;11810020;11430880;10683293;9417827;8624719;7869082;1333605;15901762;15748877;15144859;15032708;10763505;10720616;9217092;7965063
P34741	3	15033786;12115695;10933968
P34810	55	23614684;22801282;22220714;22213649;21740131;21355819;21334381;21298060;21161579;20656347;20623539;20155820;19946692;19141070;18926823;18840968;18808067;18316064;18294815;17993744;17882014;17671987;17503492;17071951;16890196;16774471;16689668;16581192;16305315;16211443;16187294;16155637;16100462;15993386;15880494;15610171;15605375;15351516;15182944;15009633;14966352;14588117;12499056;11585624;11146113;11045675;11018703;10383635;9582256;9562256;9417823;8989657;7957733;8450950;21970623

P34925	5	19473059;23320533;19473059;18701700;19473059
P34947	1	23359120
P34969	2	23481546;16836640
P34972	16	21813113;20035773;19533326;17045264;11249966;23152849;21970496;21354467;20920894;20156559;20035773;20214038;18930143;19248685;17045264;12823482
P34998	1	20470804
P35052	1	19843094
P35225	3	22420033;22233298;21411654
P35226	1	19908289
P35228	218	23826414;23688865;22981449;22825006;22708918;22342994;22233298;22150233;22125095;22072681;22002544;21940434;21922518;21854445;21787762;21656712;21626169;21529317;21383505;21355819;21190044;20799028;20423707;20370565;20210855;20176685;20109445;19891733;19741488;19519665;19416634;19295488;19183262;19141070;19026989;18948848;18855939;18665052;18628687;18367664;18322000;18255138;18197140;18187233;17945432;17918744;17892411;17712576;17704735;17645692;17500094;17418876;17382514;17287083;17181549;17017921;16978658;16786429;16776360;16703907;16648743;16601144;16506482;16303916;16184194;16100462;16041804;15895828;15671854;15640758;15618882;15605375;15567326;15520837;15486490;15335103;15229242;15036611;15009633;14753478;14662726;14656068;14644473;12865221;12810381;12713641;12680327;12675973;12490011;12232549;12042098;11835739;11834310;11453434;11393255;11342896;11170729;11146113;10697245;9729336;9547234;10921077;9000453;8863192;8903674;23731227;23700789;23688865;23659940;23135205;22748825;22125095;22002544;22000862;21626169;21596101;20633123;20468054;20382225;19741488;19675280;19651280;19418456;19291395;19056384;19026989;18855939;18096601;17712576;17645692;17618976;17484972;17385265;17353913;17287083;17258865;17217414;17017921;16978658;16791086;16786429;16703907;16671477;16246329;16179549;16100462;15895828;15640758;15486490;15353953;15335103;15229242;15036611;15036352;14993064;14767765;14753478;14753477;14666020;14644473;12903514;12865221;12810381;12713641;12675973;12490011;12402380;12232549;12133546;12042098;11844273;11835747;11835739;11834310;11453434;11427303;11425438;11424948;11253169;11200245;11191842;11146113;11068337;11043549;11031095;10984543;10928969;10925216;10923399;10790883;10697245;10686069;10486182;10471208;10455303;10393880;9871452;9871451;9848481;9795113;9760142;9507944;10921077;9416300;9208207;8880855;8863192;8791233;8710182;8861107;8903674;7478189;7753574;7525335;7530816;7687046;7684000;23688865
P35321	3	22504113;12764110;11850458
P35348	18	23178151;22081708;21222499;21047936;20822463;16198708;16143650;15680702;15486013;1353386;1030656;21047936;1675858;758380;22275761;15211988;7620299;23583569
P35354	65	23901068;21933591;21781963;17184185;15319002;15293235;15141096;11107569;11063829;11032961;10393880;9872457;9000453;8657340;1588635;3143469;3418749;22878503;22150233;21933591;21922518;21784069;21383505;21375803;21355819;21138398;20382225;20091771;19831872;19519665;19056384;18926823;18751914;18384773;18036801;17918744;17901087;17892411;17329433;17184185;16648743;16601144;16303916;16182319;16041804;15885321;15596243;15447673;15319002;15312174;15182944;15179440;14970362;14588302;14522004;12664616;12664579;11336442;11170729;11063829;11032961;11029637;10393880;9886082;9872457

P35367	1	16671478
P35372	6	19585156;16476416;16360182;15982817;9583756;8584148
P35408	1	20970752
P35580	2	22236775;19623632
P35590	1	19859984
P35613	1	17184186
P35638	9	22873727;22196867;21933012;21638341;21241777;19215662;17578450;1633360;6396563
P35716	1	20951776
P35916	5	19319198;18773817;17409380;12927201;11029637
P35968	5	19319198;18773817;17409380;12927201;11029637
P36507	4	21355419;20707645;18715715;17329433
P36544	2	23184186;16888810
P36888	1	21539498
P36894	3	21764678;21273111;21764678
P37198	1	22082874
P37231	13	23569141;23374874;22947224;21889575;21819712;21206444;20350367;18628687;17981670;17433295;17167171;17073815;21740108
P38159	1	23180094
P38936	2	17331604;11850458
P39060	1	17688061
P39900	2	22798270;14602826
P39905	123	23360280;23124646;23098734;21430166;21421025;20819171;20600315;20554000;20171177;19118106;18095482;17686909;17584113;16629625;16305315;14699980;12589927;12440384;12440372;12440358;11923434;10811391;10384258;9932426;9369296;9199395;8993706;23916821;23659940;23536080;23500094;23344852;23186720;23076378;22981449;22840455;22801282;22342994;22244304;22178331;22137336;22085254;21451028;21316362;21316153;21308793;21083432;20925481;20851742;20719091;20419536;20334467;20193555;20132484;20005873;19812967;19940184;19845866;19784741;19476208;19293775;19240691;19170182;19164849;19069631;18774604;18703596;18436292;18367307;18295206;18077567;17702601;17666954;17578287;16906542;16839548;16774476;16671480;16187294;16038451;16022680;15671854;15640758;15640751;15635609;15618882;15514921;15380482;15363220;15094497;14694501;14691064;14651811;14608602;14598294;14552891;14552879;14515351;12817305;12781986;12589929;12482120;12125080;11331375;11358454;11117507;10923678;10683293;10624807;10583476;10486182;10212315;9699155;8786386;19940184;15901762;15748877;15144859;15032708;10763505;10720616;9217092;7965063
P40189	8	23659820;20478301;18446011;18184194;16129904;15128837;15048924;20478301
P40763	21	22815920;20155820;22715999;20161735;23904622;23274522;21833847;21375803;20187447;20005873;19831872;18615534;18614693;17131417;16890196;16783372;16417589;16230180;15716400;10762348;11028697
P41143	3	17983769;16360182;9583756
P41145	12	20675200;17983769;16476416;16360182;14751594;14713862;9583756;9547096;7708296;1356430;2827851;2884046
P41159	14	24060491;22815920;22777489;22536415;22118258;21112441;19777863;18209743;16213239;15281006;12556356;12519883;11128893;9013753
P41221	4	21273111;19473059;19473059;19473059

P41231	2	20619335;21647706
P41586	1	22674051
P41594	15	22227062;21394541;19670441;16382209;16317045;16004983;15115597;11852976;11723176;11476590;9840766;19110202;16004983;11440816;11043525
P41595	16	23481546;21653706;20861436;16707714;15892603;15380867;15275776;14622228;12193190;10407057;7957735;7964712;7683713;1830095;23360270;20980537
P41597	5	20155816;18338959;21265596;18338959;12111830
P41743	2	22329943;22329943
P42081	1	21599492
P42224	2	21833847;19141070
P42226	1	17301687
P42229	4	11028697;19228956;17600519;10762348
P42261	30	23859181;22579680;21167847;20662840;19446017;18534577;16433440;15684654;15283995;12821375;12220704;10407012;22745823;22721766;21111028;20456005;20211179;19634126;18194440;17549753;15684654;15253803;14622710;12691384;11886824;11586111;10683288;10486193;10407058;8880851
P42262	36	22745823;22721766;21111028;20456005;20211179;19634126;18194440;17549753;15684654;15253803;14622710;12691384;11886824;11586111;10683288;10486193;10407058;8880851;23859181;23386718;22579680;18971481;18534577;17559837;16433440;15857397;15684654;15283995;12821375;12220704;11374812;11259116;11160435;10686069;10407012;16198697
P42263	18	22745823;22721766;21111028;20456005;20211179;19634126;18194440;17549753;15684654;15253803;14622710;12691384;11886824;11586111;10683288;10486193;10407058;8880851
P42336	15	22930444;21720761;21496122;20132484;20070863;19800972;18785877;17611275;17457363;16460709;15668227;20585375;18785877;17703359;17457363
P42338	15	22930444;21720761;21496122;20132484;20070863;19800972;18785877;17611275;17457363;16460709;15668227;20585375;18785877;17703359;17457363
P42345	22	22253859;20678995;20678995;23981724;23726960;22980985;22895182;22865656;22504113;22253859;22123082;21806471;21720761;20694004;20187447;19533653;19176818;17885021;20678995;17885021;22123082;20694004
P42574	118	23855891;23731227;23526403;23499960;23494980;23274522;23215850;23196718;23180094;23054070;22986158;22848730;22749575;22573254;22453521;22450230;22427977;22426389;22409448;22342994;22295509;22289688;22220508;21922518;21807380;21645590;21394310;21336655;21144461;21111721;21039984;20970752;20708606;20466038;20444938;20385073;20382225;20117935;20091771;20067580;20059302;19859984;19782669;19716366;19545280;19429019;19376093;19120440;18827691;18772508;18722523;18378144;18354017;18307037;18219571;18156166;18008145;17917587;17892411;17880386;17706365;17703359;17698296;17564836;17518537;17512912;17465991;17357496;17236773;17023852;16902996;16638021;16581192;16263098;16256834;16122023;16099247;16083358;16042868;16038625;16029658;16005241;15995139;15992367;15795935;15576480;15453993;15307903;15115604;14999069;14588118;14570283;14507967;13678663;12837623;12815713;12794736;12738057;12568318;12535938;12429226;11968060;11831549;11810634;11597598;11565605;11517251;11379817;11311801;11085887;11007881;10938439;10426320;22244304;22700771;21748659;17
P42771	1	19813107
P42858	1	12969257

P42898	1	19853891
P43003	9	22791629;21439271;20375134;20070863;18358622;19388351;17893916;11814437;11374812
P43004	12	23639820;21882244;21620945;21488085;21439271;20070863;19323997;18358622;18194440;17893916;11814437;11374812
P43005	3	21620945;21439271;11814437
P43115	3	22634758;20970752;17329433
P43146	5	22022865;18001205;16998900;16262652;15737738
P43250	2	23979726;23359120
P43354	2	17201484;9651546
P43490	1	22490786
P45452	1	22205935
P45983	18	21824499;18293403;16005241;22821814;22426389;22409452;21671725;21596101;21336655;20107429;19295488;19090911;18209489;17880387;16930431;16417589;16005241;10331432
P45984	16	22821814;22426389;22409452;21671725;21596101;21336655;20107429;19295488;19090911;18209489;17880387;16930431;16417589;16005241;10331432;15716400
P46013	5	23526403;23001399;22791629;21488085;19925583
P46098	6	18798253;16049141;15275776;12610685;12560138;8749032
P46527	5	22930444;23386122;22930444;22510563;21229311
P46531	14	23622458;22865656;22579680;22452482;22378895;21156172;20384775;16262629;11706559;10810480;9248556;7700587;2419108;22452482
P46663	3	17542507;22862305;17108828
P46821	1	17880387
P46939	1	23727406
P47712	15	22581384;21142140;20127525;18755070;17917587;16566026;16498630;15248295;11499402;8522975;7473799;19409102;19371144;19306380;10527456
P47869	13	23542439;23332495;22449374;20203195;19358834;19225548;18845615;18206170;17376001;17046753;15157699;12618352;18495826
P47870	13	23542439;23332495;22449374;20203195;19358834;19225548;18845615;18206170;17376001;17046753;15157699;12618352;18495826
P47898	1	23360270
P47900	1	21647706
P47989	5	17535569;15610928;12423257;9000453;1649310
P48023	34	22038545;21181266;17518537;10912923;23935974;23647386;23184030;22733360;22342994;21787762;21496122;21181266;20830289;20385073;20370565;20304963;20176685;20107429;19891733;19183262;19120440;18948848;18628687;18180375;17945432;17645692;17621255;17518537;16689665;15567326;15004554;11170729;10912923;11170729
P48039	1	20626592
P48058	27	22745823;22721766;21111028;20456005;20211179;19634126;18194440;17549753;15684654;15253803;14622710;12691384;11886824;11586111;10683288;10486193;10407058;8880851;23859181;22579680;15684654;15283995;12821375;12220704;11259116;10407012;8994060
P48061	12	23197665;22842524;22172378;22049432;21806987;21332278;21324162;20374199;19756447;17884290;17061258;23197665
P48067	1	21620945

P48167	7	23248270;21775715;20733588;20190766;17046753;12957506;12618352
P48357	1	22815920
P48431	14	23419261;23169458;22865656;22791629;22537391;22042562;21949375;21513774;20161735;20030221;19925583;19476208;18712509;16087243
P48436	3	23027386;22791629;17597120
P48454	8	23981724;18577426;14767765;12429226;11060810;11007881;9878202;8791233
P48506	2	21806470;9726263
P48507	2	21806470;9726263
P48547	1	21110920
P48551	1	22161971
P48681	67	23419550;23419261;23281512;23143000;23106570;22791629;22500090;22038821;21956379;21842261;21630007;21513774;21375803;21330787;21251802;20951776;20678995;20673789;20155816;20151365;20100476;20083105;20059998;19925583;19350385;19191513;19163037;19069631;18971163;18611436;18583926;18197384;17612595;17506499;17409380;17184184;17044031;16406666;16339643;16087243;16038450;15869942;15857397;15680701;15464285;15390101;15335106;15288436;15129154;15051157;14610361;12817305;12655601;12578228;12429182;12379246;12205672;11940359;11893024;11810020;11773606;11746449;11260912;11161592;10331437;10225952;9248556
P49137	1	20943915
P49238	4	23664962;23361876;21734283;23361876
P49286	1	20626592
P49763	2	22023610;20860549
P49768	1	19932144
P49841	10	22514792;21898827;21720761;20103844;19533653;18768685;17233289;16601144;16511963;15307905
P49862	3	24128681;24128681;22529377
P50052	1	23174180
P50553	3	20951776;16497507;17108169
P50591	2	20107429;11170729
P50613	1	21710280
P51582	1	21647706
P51587	1	21748659
P51649	4	15470819;12955874;9887150;1534322
P51805	2	20702718;20702718
P51946	1	21710280
P51948	1	21710280
P52333	5	17131417;16957585;16890196;22715999;20161735
P52565	3	19651108;16624299;15799964
P52797	1	21603973
P52798	1	22350947
P52799	2	23518227;16623828
P52803	3	21603973;21931787;20202079
P53420	7	21898827;17314203;16629627;11920709;11564534;11356383;11104506

P53778	54	22865681;23361876;22733360;22634758;21310020;20536940;20444938;20381564;19383246;19196180;19090911;18630599;17329433;15707984;11031088;23688865;23404572;22821814;22733360;22634758;22525836;21922518;21394310;20954833;20943915;20444938;20382225;20161735;19846725;19765637;19699199;19418456;19409102;19090911;18708926;18590729;18562123;18511041;18209489;17634369;17443214;16978658;16930431;16511963;16478624;16422251;16187294;16038625;15843065;15320513;14512145;12810381;10331432;23688865
P53779	20	22821814;22426389;22409452;21671725;21596101;21336655;20107429;19295488;19090911;18209489;17880387;16930431;16417589;16005241;10331432;23303920;22700771;21824499;17670986;22700771
P54289	1	21239111
P54619	2	24022865;24022865
P54646	4	17885021;24022865;22865656;17885021
P54652	3	16550329;11994804;9416301
P54707	1	22275761
P54764	7	23557244;22900481;22824304;21931787;17418490;16959251;15537875
P54803	4	21375803;20374080;10924962;1281366
P54821	1	21187959
P55008	13	23419864;23399872;22878925;22722907;22510563;22038545;21620945;20219534;19780197;16155637;11585624;11563627;11064366
P55011	3	21333799;20536931;18799000
P55055	2	19891733;19891733
P55085	1	23647384
P55087	19	23831998;23441695;23234244;23098724;21280976;21092735;20590523;20517941;20423707;20109536;19151597;18951883;18267965;18248364;18065151;17074445;16817282;16219025;16089144
P55107	1	21196217
P55210	3	22244304;22244304;20816819
P55211	10	22244304;23494980;20816819;19120440;18722523;17706365;15796358;22700771;21748659;17298387
P55212	1	17706365
P55851	1	22865656
P55895	1	22868200
P56159	7	21316153;16906542;15635609;14608602;11331375;19235905;16187294
P56199	2	17161391;17161391
P56373	1	22540742
P56693	5	21538562;21034515;20083105;18756526;16691121
P56704	4	21273111;19473059;23456240;21249402
P60033	3	16806185;11240026;11064366
P60201	9	23832758;19053058;17376002;15857397;14599661;14534257;10833310;10196465;9786985
P60484	11	24068802;23726960;23647386;22253859;22123082;20816819;20694004;17941496;16003541;22123082;20694004
P60568	8	23731227;22189457;14760011;14563689;12165135;10052669;9014956;8426182
P60709	8	21994364;20200564;17418490;17184189;16083359;16083356;9344569;8926629
P60953	3	21375803;19943951;15736231

P61073	9	23197665;22842524;22049432;21332278;21324162;21196217;20374199;20155816;17061258
P61278	10	23333673;19001189;12187277;11898691;10853753;8988397;7478725;2426476;2410810;836537
P61586	70	24297045;24246280;23830951;23360272;22902990;22773197;22733360;22700771;22350947;22239108;21848846;21781963;21459564;21447094;21443453;21381984;21375803;21308793;21283639;21193903;20406886;20203533;20202079;19955379;19943951;19925560;19623163;19473059;19324014;19125588;19061375;18980474;18692574;17692017;17634369;17618980;17011208;16990586;16629619;16629618;16624299;16585268;16154567;15885321;15880494;15845084;15799964;15736231;15293235;15219678;15031718;12860969;12691734;12598630;12480155;12440379;12165352;12151536;8158556;2263408;24297045;21815784;19623163;18722369;17217414;22700771;19519335;17692017;12691734;18980476
P61812	26	23727390;23562792;22079829;22016551;21787762;21760535;21273450;20623539;19719963;19342245;18581269;18293403;18221366;17611274;16498024;15380482;15145083;14637102;14552879;12127673;12056841;8963994;8834102;7846578;8091423;15998793
P61978	1	19323997
P61981	2	23177959;22110655
P62158	5	23255597;22027236;20849836;11825508;10700012
P62166	1	20585375
P62258	4	23177959;22110655;20399821;20141154
P62328	1	19861493
P62805	2	24336730;21439269
P62937	1	12850583
P63000	4	21925174;21375803;19052208;15736231
P63098: Q96L	8	23981724;18577426;14767765;12429226;11060810;11007881;9878202;8791233
P63104	1	21559420
P63165	1	23054070
P68431	3	22801282;22409448;21439269
P70658	1	23880092
P78348	2	23224900;21725232
P78352	3	22052241;21658682;18053028
P78357	4	22407783;20610764;18400887;14984408
P78423	3	23361876;18511041;23361876
P78426	3	22473852;19955358;18635866
P78504	3	22452482;21156172;22452482
P78509	1	20610764
P80294	1	17218363
P84022	2	23727390;22208735
P98170	5	18367607;11379817;11063050;10658186;8786386
Q00535	3	22514792;15715081;15715081

Q00653	64	23880092;23574812;23553703;23499960;23492769;23361876;23359120;22981449;22853439;22798270;22733360;22578249;22484641;22450230;22324804;22289688;22125095;22038545;21889575;21784069;21510818;21383505;20954833;20868716;20862369;20830289;20799028;20708606;20646530;20370565;20176685;20091771;20047904;19891733;19716366;19522780;19418318;19416634;19358984;19295488;19056384;18948848;18665052;18628687;18197140;18180375;17918744;17664140;16776360;16253423;16179517;15998793;15922485;15684769;15478370;15452194;15229242;15135227;11517251;11393255;11150324;10428065;9729336;9547234
Q01064	1	21297958
Q01094	3	22848730;22510563;22848730
Q01581	1	20399821
Q01668	2	17291691;22081708
Q01668	5	17448606;14753480;10924962;10222112;9007541
Q01860	13	23419261;22865656;21949375;21538562;20875224;20374080;20161735;20030221;19476208;19318112;19053058;18712509;14534257
Q01959	3	18992013;16503802;11259109
Q01995	2	21655070;16344894
Q02153	1	21145575
Q02223	1	23088438
Q02246	5	23285014;19458237;20610764;19458237;14984408
Q02297	13	23935974;23758598;23097328;22042562;21539896;20734425;19780197;18756526;16005441;14618270;11797086;23758598;23097328
Q02363	1	21679705
Q02750	7	22824323;19469685;11031088;21355419;20707645;18715715;17329433
Q02763	3	22996658;20375135;19859984
Q02962	1	22473852
Q03060	3	16088035;14662791;12721213
Q03135	2	17993744;17275798
Q03181	5	23374874;21685825;20176685;20058304;21740108
Q03405	2	21573723;19651246
Q03431	8	22710945;22218778;17376000;11853032;9427170;8830990;8786732;8024422
Q04206	8	22189437;21134362;19716366;19469692;17918744;16179517;9547234;17918744
Q04917	1	23177959
Q05329	9	23103212;22569103;20132484;20059302;18353556;17559837;10740227;23152062;21620945
Q05513	2	22329943;22329943
Q05586	92	22579680;22046257;21756907;20226782;19765637;18835424;17559837;16460709;16099549;15922485;15857397;15684654;15283995;12946581;12220704;11259116;11158251;11033004;10886684;10854260;10686069;9872459;23618680;22650041;22650040;22449374;22402659;21756907;20675200;20495588;20211179;19939961;19923273;19772458;19668258;19407255;19026989;19019202;19005051;18635178;18484790;18177675;18053028;17983769;17287199;16703907;16678969;15950221;15901762;15748877;15640756;15313031;14758350;12903514;12885421;12162901;11992467;11107569;11101210;11043549;11007881;10984543;10720616;10674755;10486193;10419375;9918969;9773443;10921077;9048311;9002066;8985951;9594166;8880851;8657340;8584148;7472559;7889371;8207481;7909561;8137170;8103243;1588610;1311880;1837648;2163498;2155794;2377900;1982014;2841902;3057216;16198697

Q06830	2	19119913;21187959
Q06945	1	21196217
Q07157	5	22798270;22409448;20590523;19891526;18092342
Q07343	4	23028463;23028463;22865690;18455876
Q07812	57	23274522;19525878;23731227;23492769;23344852;23274522;23180094;22733360;22453521;22427977;22342994;22295509;22289688;21787762;21748659;21496122;21111721;20830289;20799028;20708606;20385073;20370565;20176685;20153400;20107429;19891733;19815005;19525878;19473058;19373550;19183262;18948848;18838947;18665052;18628687;18322000;18209489;18197140;18180375;17945432;17704735;17664140;17645692;17621255;17465991;17418876;17331604;17304106;16601144;16303916;15668909;15115604;14507967;12794736;11810634;10709875;20001688
Q07869	10	23374874;22537532;21963672;19926854;19416634;18628687;18367664;17073815;15899263;21740108
Q07954	2	23867460;19651246
Q08043	1	23145141
Q08050	1	23386122
Q08209	8	23981724;18577426;14767765;12429226;11060810;11007881;9878202;8791233
Q08462	6	20141154;19119913;18677446;15698618;10804204;3548963
Q08499	1	23028463
Q08722	2	21970599;21168495
Q08828	7	23349859;20141154;19119913;18677446;15698618;10804204;3548963
Q09428	11	23255597;22714048;22197047;22177998;22078759;21492614;21145891;20410530;18032110;17657312;23255597
Q09666	1	16930430
Q12778	1	19533653
Q12791	1	22534235
Q12794	4	21111028;15892130;7355381;426486
Q12860	4	17425960;20610764;19458237;14984408
Q12879	82	23618680;22650041;22650040;22449374;22402659;21756907;20675200;20495588;20211179;19939961;19923273;19772458;19668258;19407255;19026989;19019202;19005051;18635178;18484790;18177675;18053028;17983769;17287199;16703907;16678969;15950221;15901762;15748877;15640756;15313031;14758350;12903514;12885421;12162901;11992467;11107569;11101210;11043549;11007881;10984543;10720616;10674755;10486193;10419375;9918969;9773443;10921077;9048311;9002066;8985951;9594166;8880851;8657340;8584148;7472559;7889371;8207481;7909561;8137170;8103243;1588610;1311880;1837648;2163498;2155794;2377900;1982014;2841902;3057216;16198697;19446017;18835424;18534577;17853656;16099549;15283995;12220704;11259116;11160435;11033004;10886684;10854260
Q13002	2	19224535;8994060
Q13003	1	8994060
Q13018	1	19306380
Q13093	1	16339448
Q13114	1	22484641
Q13118	1	17167171
Q13127	1	21671725
Q13131	2	22865656;17885021
Q13133	3	23433337;19891733;19891733

Q13158	5	23935974;23935974;23574812;17518537;11208917
Q13164	15	22865681;23361876;22733360;22634758;21310020;20536940;20444938;20381564;19383246;19196180;19090911;18630599;17329433;15707984;11031088
Q13177	1	10426320
Q13214	1	12009760
Q13224	79	23618680;22650041;22650040;22449374;22402659;21756907;20675200;20495588;20211179;19939961;19923273;19772458;19668258;19407255;19026989;19019202;19005051;18635178;18484790;18177675;18053028;17983769;17287199;16703907;16678969;15950221;15901762;15748877;15640756;15313031;14758350;12903514;12885421;12162901;11992467;11107569;11101210;11043549;11007881;10984543;10720616;10674755;10486193;10419375;9918969;9773443;10921077;9048311;9002066;8985951;9594166;8880851;8657340;8584148;7472559;7889371;8207481;7909561;8137170;8103243;1588610;1311880;1837648;2163498;2155794;2377900;1982014;2841902;3057216;16198697;22424878;21167847;20079352;18835424;17559837;15283995;14654094;12220704;10886684
Q13253	10	23859181;21508230;20850923;20005873;18221366;17151948;16938843;16087174;15296834;11078560
Q13255	12	23570727;22227062;16004983;15115597;11852976;11825670;11723176;11476590;19110202;16004983;11440816;11043525
Q13275	2	17278142;12009760
Q13304	1	19528093
Q13309	1	23386122
Q13315	7	21748659;18256991;12236201;8686572;8475307;8145266;7117323
Q13332	3	22836147;20179269;19780196
Q13349	29	22150233;22078757;22044160;21784069;21145887;21142687;20146558;20082560;19250688;17048772;16379576;16198705;15992367;15890340;15659600;15465595;15312174;15102919;15009633;22150233;22044160;21784069;21145887;19250688;15992367;15659600;15312174;15102919;15009633
Q13393	2	23031841;12499056
Q13464	23	24297045;21815784;19623163;18722369;17217414;19519335;17692017;12691734;18980476;22733360;20141964;19955379;19651108;17692017;16624299;16154567;15880494;15799964;15293235;15219678;12480155;12151536;11471206
Q13467	1	23251385
Q13489	9	11517251;11379817;11379817;11063050;11063050;10658186;10658186;8786386;8786386
Q13490	2	22450230;11517251
Q13509	8	23419261;21573803;20100476;19934391;19462232;18721107;15051137;116023[illegible]
Q13516	14	14648541;23460872;22496912;22473852;22173726;21538562;19772605;19053058;18992013;18756526;18716209;17108169;14534257;21538562
Q13541	1	19533653
Q13546	1	17044031
Q13554	12	23707296;22911773;22384207;22296735;22027236;21167847;20070863;19409102;19332038;17719180;17592948;17258865
Q13635	2	22391313;19955358
Q13639	1	15817810
Q13698	6	17448606;14753480;10924962;10222112;9007541;22081708

Q13813	10	19376093;19111721;18289533;17880386;17181549;15893424;12821393;12614588;12207572;9535120
Q13873	1	21764678
Q13936	7	17291691;17448606;14753480;10924962;10222112;9007541;22081708
Q14005	1	16572623
Q14116	3	23140983;22378878;18367607
Q14145	1	22853439
Q14194	1	22181040
Q14195	1	22181040
Q14249	1	16689664
Q14254	1	22878913
Q14416	6	19110202;16004983;11440816;11043525;21451028;11771948
Q14432	1	20830289
Q14457	3	23852559;21854445;20210855
Q14512	1	17553847
Q14563	12	23116416;21640756;17997325;17278142;17099709;16860320;16540569;16230022;16198706;15094469;14727128;12009760
Q14624	1	21559420
Q14653	1	22161971
Q14721	1	21110920
Q14764	1	23106570
Q14765	1	17301687
Q14790	4	17518537;12568318;11379817;19006686
Q14832	6	19110202;16004983;11440816;11043525;21451028;11771948
Q14833	4	19110202;16004983;11440816;11043525
Q14957	69	23618680;22650041;22650040;22449374;22402659;21756907;20675200;20495588;20211179;19939961;19923273;19772458;19668258;19407255;19026989;19019202;19005051;18635178;18484790;18177675;18053028;17983769;17287199;16703907;16678969;15950221;15901762;15748877;15640756;15313031;14758350;12903514;12885421;12162901;11992467;11107569;11101210;11043549;11007881;10984543;10720616;10674755;10486193;10419375;9918969;9773443;10921077;9048311;9002066;8985951;9594166;8880851;8657340;8584148;7472559;7889371;8207481;7909561;8137170;8103243;1588610;1311880;1837648;2163498;2155794;2377900;1982014;2841902;3057216
Q15077	1	21647706
Q15078	4	15715081;5348874;15715081;15715081
Q15109	2	20195207;11188567
Q15303	4	23758598;23097328;20734425;11797086
Q15327	1	21367919
Q15375	5	20949525;12075988;16983667;12075988;21603973
Q15386	1	17218363
Q15389	6	23562792;22023610;22020092;20860549;20799882;20375135
Q15465	18	23859181;23177959;22579680;22391313;22243800;21196217;21107202;20384775;19955358;19383401;19278333;18635866;18029281;17978191;16262629;14749190;12859063;12520472
Q15628	1	23574812

Q15759	39	23688865;23404572;22821814;22733360;22634758;22525836;21922518;21394310;20954833;20943915;20444938;20382225;20161735;19846725;19765637;19699199;19418456;19409102;19090911;18708926;18590729;18562123;18511041;18209489;17634369;17443214;16978658;16930431;16511963;16478624;16422251;16187294;16038625;15843065;15320513;14512145;12810381;10331432;23688865
Q15768	4	22900481;22411787;17978191;22350947
Q15796	1	22208735
Q15797	2	24336730;21518886
Q15842	1	17108688
Q15848	3	24060491;22815920;16213239
Q16099	9	19224535;15901762;15748877;15144859;15032708;10763505;10720616;9217092;7965063
Q16181	1	20399821
Q16236	11	22853439;23523995;23215850;21926641;21806470;21764072;21176673;20862369;19589199;17912625;21806470
Q16288	21	23867460;23856436;23792206;23746279;23174180;21783247;21441969;20171177;18585435;18253945;17522325;17316612;12774239;11550223;11161589;11077420;10993692;9391013;8298102;1333605;23792206
Q16394	1	23217490
Q16478	10	19224535;15901762;15748877;15144859;15032708;10763505;10720616;9217092;7965063;8994060
Q16539	39	23688865;23404572;22821814;22733360;22634758;22525836;21922518;21394310;20954833;20943915;20444938;20382225;20161735;19846725;19765637;19699199;19418456;19409102;19090911;18708926;18590729;18562123;18511041;18209489;17634369;17443214;16978658;16930431;16511963;16478624;16422251;16187294;16038625;15843065;15320513;14512145;12810381;10331432;23688865
Q16552	3	22985669;22875000;21034793
Q16555	3	22181040;20438770;20141154
Q16558	1	22534235
Q16568	1	23151374
Q16572	3	17229408;16433440;16300756
Q16612	2	21430360;21157914
Q16619	1	14715438
Q16620	56	23583688;22027236;20079352;19703592;19222991;23884949;23856436;23792206;23583688;23174180;22423083;22244304;22027236;21476782;21421025;21358740;21110803;20536940;20209974;20171177;20079352;19923273;19788572;19703592;19545610;19407255;19222991;19182380;18672032;18585708;18585435;18320028;18305238;17719180;17522325;17376001;17234430;16632872;15901762;15707984;15611995;15296835;12774239;12189689;12115676;11430880;11331375;11161589;11077420;10993692;10757326;10712659;9391013;8298102;1333605;23792206
Q16625	5	22798270;22409448;21298060;20590523;18092342
Q16627	9	22730180;22510563;22407783;21704617;20607865;17506499;17390308;15995139;15739189
Q16653	13	23642707;21534729;21110803;19672953;19580419;19053058;18930141;17906634;17630211;15548666;15319003;15031718;14563689
Q16659	15	22865681;23361876;22733360;22634758;21310020;20536940;20444938;20381564;19383246;19196180;19090911;18630599;17329433;15707984;11031088

Q16665	6	23234244;21396163;21092735;18248364;16044166;15161688
Q16739	2	15668227;15229242
Q16836	1	23011062
Q3LIF1	56	23790207;23642707;23592243;23036616;22350947;22178331;21995852;21815784;21699896;21530508;21110803;20590535;20582944;20515570;20484625;20478301;20374087;19757023;19372269;19291225;18930141;18820405;18703145;18508036;18215231;18194520;17975707;17724451;17429219;17394135;17141961;16697217;16629624;16173073;16092935;15634734;15525355;15525289;15504325;15128857;14697671;14618270;14563689;12812757;12718856;12718855;12718854;12658441;12431229;12037567;11978832;11752461;11690617;11331396;11358445;11050127
Q53EL6	2	23647386;20816819
Q58F15	15	20949525;12075988;23557244;22824304;22629434;22193443;21931787;20202079;20170651;17970742;17418490;16959251;16623828;15537875;21603973
Q5T230	1	19476208
Q5T4W7	3	22137336;19293775;16022680
Q5TA89	1	22452482
Q5XPI4	1	21229311
Q63HR2	4	24068802;23726960;23647386;16003541
Q6NW40	1	19324014
Q6RSH7	1	19838135
Q6UVK1	139	23562508;23499793;23447612;23320533;23289019;23169458;23066785;23061434;23049984;23027386;22917776;22865681;22836147;22728374;22629425;22510563;22500090;22465128;22459192;22265655;22243800;22205935;22042562;22020092;22016526;21970623;21952042;21885201;21871887;21848846;21756907;21753849;21753237;21704617;21674488;21630007;21630006;21596037;21402118;21337374;21283639;21215745;21105148;20925481;20887664;20869112;20620201;20607865;20558254;20552220;20534825;20179269;20155816;20130176;20102265;20079803;20058304;20053907;19960516;19884507;19833921;19793972;19780197;19780196;19659409;19604403;19458241;19319198;19257808;18930033;18926822;18756526;18722369;18715114;18486695;18373483;18353313;18001203;18000864;17936753;17597120;17567803;17540369;17506499;17438016;17432960;17390308;17330874;17329414;17184186;17184184;17141961;17108169;17050723;17014846;16965762;16902766;16862564;16705682;16691121;16672645;16632872;16629625;16624960;16503802;16367770;16240391;16087243;16051494;16005441;15869942;15845083;15755547;15739189;15736057;15390101;15378660;15145083;15080887;15016081;14993064;14960611;14561854;14534257;14534158;12895450;12821386;12691734;12526031;12440375;11948352;11923444;11331369;11273643;11011818;10630190;10483914;9878200;9417833
Q6UXS9	4	21807380;19215662;17672973;17578450
Q76LX8	1	22425718
Q7Z444	3	19476208;17961063;15264780
Q86UN2	1	22023338
Q86UN3	5	22836147;21699896;20815818;17234430;22023338
Q86V65	#N/A	21297958
Q86V67	11	23499793;23295392;23028463;22865690;21355819;20338167;19635528;18455876;18289533;15173585;11773606
Q86VB7	2	23775900;17882014
Q86W47	1	22534235

Q86W56	1	16825529
Q86Y38	5	23027386;22721770;21885201;18765417;14960611
Q86YM7	1	23386718
Q8BIF2	4	18354017;17392476;15892602;15129757
Q8IUQ4	1	21336655
Q8IWA4	1	23727406
Q8N5V2	2	20949525;20202079
Q8NAP3	1	22427977
Q8NER1	12	17108828;23108547;22037502;21265596;20970752;18824169;18321652;18293403;18249134;17542507;17346705;15998524
Q8NI17	1	21611127
Q8TAK6	1	21538562
Q8TCU5	69	23618680;22650041;22650040;22449374;22402659;21756907;20675200;20495588;20211179;19939961;19923273;19772458;19668258;19407255;19026989;19019202;19005051;18635178;18484790;18177675;18053028;17983769;17287199;16703907;16678969;15950221;15901762;15748877;15640756;15313031;14758350;12903514;12885421;12162901;11992467;11107569;11101210;11043549;11007881;10984543;10720616;10674755;10486193;10419375;9918969;9773443;10921077;9048311;9002066;8985951;9594166;8880851;8657340;8584148;7472559;7889371;8207481;7909561;8137170;8103243;1588610;1311880;1837648;2163498;2155794;2377900;1982014;2841902;3057216
Q8TD08	15	22865681;23361876;22733360;22634758;21310020;20536940;20444938;20381564;19383246;19196180;19090911;18630599;17329433;15707984;11031088
Q8TD43	4	23255597;23255597;22177998;19169264
Q92186	3	22460918;21264949;17363265
Q92562	1	22157617
Q92570	2	17881483;17201484
Q92633	1	20495828
Q92686	1	10737454
Q92731	2	22700771;16902996
Q92752	9	22902990;19757023;19337830;19150614;16870730;15525355;11733702;11085900;10483914
Q92854	1	14534257
Q92876	9	24128681;24128681;23832758;23647384;23376368;16987227;11186232;23832758;23647384
Q92886	1	22182208
Q92913	1	18581269
Q92934	36	22982298;22865656;22409448;22253859;21970623;21496122;20678995;20585375;19533653;19176818;19109493;18785877;18768685;18305238;18236467;17885021;17553847;17457363;17233289;16957585;16511963;15896972;23731227;23433337;23344852;22453521;19716366;19530162;19228956;19120440;18354017;18302959;17600519;17551978;15668909;11721745
Q93063	1	23217490
Q969P5	4	21221630;19533653;18653749;17218363
Q969Q1	7	21854445;21221630;19533653;19214561;18653749;18236467;17218363
Q96B36	1	17457363
Q96B86	8	22330947;19324014;19268666;17996222;17692017;17169331;16585268;15843084
Q96BE9	#N/A	22510563;12666113

Q96C10	1	22161971
Q96DB9	1	22275761
Q96EB6	1	23055316
Q96FE5	8	[illegible]
Q96FF9	8	[illegible]
Q96GW7	167	24478364;24044287;23861090;23839053;23790207;23702616;23642707;23562508;23520469;23447612;23289019;23066785;23061434;23049984;23027386;23022460;22978525;22917776;22836147;22728374;22723886;22684804;22629425;22525836;22465128;22420304;22350947;22343313;22265655;22205935;22137336;22133879;22079829;22042562;22020092;22016526;21970342;21952042;21946272;21898827;21885201;21871887;21848846;21783247;21756907;21753849;21753237;21674488;21630006;21596037;21486314;21402118;21360238;21316362;21283639;21273450;21264949;21215745;21105148;21092402;20925481;20887664;20869112;20620201;20558254;20552220;20534825;20179269;20130176;20102265;20079803;20053907;19955379;19940184;19884507;19833921;19793972;19780196;19672039;19659409;19631747;19604403;19603426;19522780;19382871;19165795;19125588;19074020;19005065;18930033;18926822;18786615;18768685;18765417;18722369;18715114;18486695;18484102;18373483;18353313;18065151;18001203;18000864;17936753;17597120;17585905;17567803;17540369;17438016;17329414;17223033;17184186;17141961;17050723;17005848;16965762;16902766;16862564;16837588;16705682;16691121;16672645;16629625;16629623;16624960;16367770;16240391;16099038;15998793;15869942;15755547;15736057;15716629;15672632;15530871;15341588;15307905;15174067;15145083;15128857;15080887;15016081;14993064;14960611;14561854;14534158;12895450;12821386;12811811;12691734;12573465;12526031;12440375;12151546;12151536;12040068;11948352;11923444;11425904;11273643;11011818;10630190;104839
Q96HS1	2	20001688;23731227
Q96KE0	1	16145477
Q96NK8	1	22466292
Q96P20	1	22733360
Q96QT4	1	23828570
Q99062	20	23433334;23419550;23209732;23139012;23101949;22862301;22391867;22024901;21935680;21721873;20887150;20202082;19499175;18406145;17882016;17679775;17618991;17391650;16958589;12201902
Q99259	13	20663524;20132484;20059302;19225548;18480373;18353556;18180106;16626970;15233942;10740227;10619473;23152062;21620945
Q99453	1	22673527
Q99527	6	20456002;20350367;17704735;16902996;23164781;22975889
Q99571	3	23404572;22378878;15885321
Q99572	8	[illegible]
Q99574	1	23163103
Q99576	1	22125095
Q99683	2	18708926;10331432
Q99714	1	23011062
Q99748	1	21196217
Q99836	2	19932745;17403033
Q99941	3	22873727;21933012;21638341

Q99942	21	23447612;22993437;21970342;21470565;19458241;17601981;17506499;17239557;17184184;17141961;16902766;16874803;16691121;16503802;15869942;15845083;15378660;15080887;14561854;11331369;11273643
Q99985	2	12009760;18308469
Q9BPU6	1	22181040
Q9BSL1	1	21229311
Q9BU40	1	20459000
Q9BX97	1	20590523
Q9BXH1	1	23492769
Q9BXW4	1	22082874
Q9BYX4	2	22161971;22161971
Q9BZR6	51	15031718;23592243;22836147;22827732;22728374;22236767;22162062;22023338;21891937;21815784;21784510;21699896;21176674;21110803;21087927;20819171;20815818;20809785;20725955;20702718;20484625;20203533;20179954;20092707;19439611;18930141;18820405;18692574;18394723;18234196;18056009;17913768;17630211;17567803;17362886;17234430;17011208;16958113;16497507;16101740;16092935;15866044;15548666;15504325;14668808;14618270;12764110;12658441;12037567;20702718;16092935
Q9C000	1	22378878
Q9GZQ8	6	23852559;22082874;21854445;21806471;21304420;20210855
Q9GZZ7	2	19235905;16187294
Q9H244	1	21647706
Q9H2A3	2	18635866;17108169
Q9H2U1	2	22853439;21806470
Q9H2X9	6	23248270;21333799;20536931;20190766;18845615;18799000
Q9H3M7	1	22981449
Q9H4L4	1	23054070
Q9H598	3	21620945;16300756;11723173
Q9H633	6	17376001;12611774;12060811;10886684;10709875;10586102
Q9H9S0	6	23419261;20875224;20161735;20030221;19476208;18712509
Q9HBA0	1	18206306
Q9HBW0	1	20495828
Q9HCM2	1	20702718
Q9HD89	1	22815920
Q9NP80	1	21868473
Q9NPA1	1	22534235
Q9NPG1	1	21196217
Q9NPG2	2	23658829;22105458
Q9NPH5	1	23152062
Q9NQB0	2	20849836;18236467

Q9NQC3	81	23790207;23211464;23158140;23036616;22980985;22306041;22236767;22162062;22137336;21995852;21815784;21596039;21530508;21176674;21087927;20849836;20725955;20702718;20673791;20590535;20547125;20515570;20484625;19955379;19619659;19587271;19442692;19436077;19372269;19337830;19291225;19158290;18980476;18980474;18930141;18820405;18703145;18508036;18394723;18341395;18221191;18194520;18056009;17975707;17913768;17692017;17538769;17429219;17394135;17011208;16998900;16697217;16629624;16497507;16324097;16173073;16101740;16092935;15866044;15799964;15548666;15525289;15504325;15031718;14618270;14563689;12812757;12764110;12718856;12718855;12718854;12658441;12598630;12183616;12037567;11978832;11752461;11358445;11343640;20702718;16092935
Q9NR23	1	20875224
Q9NR96	1	23313320
Q9NR97	1	19688331
Q9NRA1	14	22407783;22236767;21568693;20819515;19818206;19383401;17597120;14534257;10329979;9618703;8963994;7657809;8091423;18925432
Q9NRD8	5	21394541;19056384;17071951;15992367;15036352
Q9NTK1	1	22815920
Q9NWT6	8	23234244;21331619;20010384;19076458;15663420;15161688;12399425;119040[illegible]
Q9NXG6	2	19076458;11564534
Q9NZ45	1	23627890
Q9NZJ5	7	22873727;22815920;21933012;21638341;18785877;18630599;16478624
Q9NZK7	1	19306380
Q9NZQ7	1	21481304
Q9P0K8	1	22246994
Q9P0X4	1	19005061
Q9P2N4	2	23562508;23562508
Q9P2U7	5	23061434;22171058;21697383;17577523;17134699
Q9P2U8	2	17577523;16300756
Q9UBC3	1	20875224
Q9UBK2	2	22208735;21171097
Q9UBL9	2	21647706;22540742
Q9UBS5	5	18495826;23904624;22721766;22449374;16866624
Q9UBU3	5	22194122;21625243;20497419;20444938;16213239
Q9UBY5	1	20495828
Q9UGI9	1	24022865
Q9UHD4	1	16933827
Q9UHI8	2	23562508;23562508
Q9UJY1	3	23143993;18156166;15221884
Q9UKJ0	3	21699896;20714874;20484625
Q9UKL4	1	21333809
Q9UKQ9	3	24128681;24128681;24128681
Q9UKX2	30	24022865;23195635;20813653;19705475;19623632;18566944;18372308;17717118;16931550;16778003;16239281;15470819;15468195;15221880;12383078;11994962;11686494;11268024;11216861;11138583;10668768;10567091;10024136;9887150;9755066;9044514;9025213;8756163;8596693;8722515
Q9UKX3	1	20813653
Q9UL19	1	22161971

Q9UM11	1	21679705
Q9UM81	1	19833921
Q9UN79	1	23169458
Q9UNA0	3	23562508;23562508;23562508
Q9UNG2	2	20107429;18322000
Q9UQD0	1	21177993
Q9UQM7	14	19332038;17258865;23707296;22911773;22384207;22296735;22027236;21167847;20070863;19409102;19332038;17719180;17592948;17258865
Q9UQQ2	1	19859984
Q9Y243	24	22253859;20678995;22982298;22865656;22409448;22253859;21970623;21496122;20678995;20585375;19533653;19176818;19109493;18785877;18768685;18305238;18236467;17885021;17553847;17457363;17233289;16957585;16511963;15896972
Q9Y275	1	23088438
Q9Y2C9	1	21970496
Q9Y2I1	3	22985669;21303658;12079683
Q9Y314	1	23135205
Q9Y337	2	24128681;24128681
Q9Y345	3	21620945;22171058;17134699
Q9Y3D6	1	23727406
Q9Y466	1	21258860
Q9Y478	3	24022865;22865656;17885021
Q9Y5N1	1	16671478
Q9Y5U5	1	18322000
Q9Y5Y9	2	20566409;12560118
Q9Y691	1	22534235
Q9Y6E0	1	19855390
Q9Y6M5	3	22565470;21752551;20838921
Q9Y6N7	2	19783284;19783284
