# Supplementary material for: Molecular Architecture of Spinal Cord Injury Protein Interaction Network
Source: PLoS One. 2015 Aug 4;10(8):e0135024. doi: 10.1371/journal.pone.0135024 (PMC4524728; doi:10.1371/journal.pone.0135024)
Supplement: S4 Table — (PDF) [file pone.0135024.s006.pdf]

Supplementary Table IV. Gene Ontology (GO) biological processes over-representation in the entire 1083 accessions performed through DAVID.								
Category	Term	Count	%	PValue	Fold Enrichment	Bonferroni	Benjamini	FDR
GOTERM_BP_FAT	GO:0009611~response to wounding	198	18.5	3.70E-88	4.901897773	1.71E-84	1.71E-84	6.98E-85
GOTERM_BP_FAT	GO:0010033~response to organic substance	228	21.3	1.51E-85	4.1492969	6.96E-82	3.48E-82	2.85E-82
GOTERM_BP_FAT	GO:0010941~regulation of cell death	240	22.4	3.27E-83	3.863923881	1.51E-79	5.03E-80	6.17E-80
GOTERM_BP_FAT	GO:0042127~regulation of cell proliferation	235	21.9	1.11E-82	3.918032726	5.10E-79	1.28E-79	2.09E-79
GOTERM_BP_FAT	GO:0043067~regulation of programmed cell death	238	22.2	5.46E-82	3.845881133	2.52E-78	5.03E-79	1.03E-78
GOTERM_BP_FAT	GO:0042981~regulation of apoptosis	234	21.8	8.41E-80	3.818868799	3.88E-76	6.47E-77	1.59E-76
GOTERM_BP_FAT	GO:0007267~cell-cell signaling	190	17.7	1.92E-70	4.155059812	8.86E-67	1.27E-67	3.62E-67
GOTERM_BP_FAT	GO:0007610~behavior	167	15.6	5.32E-70	4.672169153	2.46E-66	3.07E-67	1.00E-66
GOTERM_BP_FAT	GO:0008284~positive regulation of cell proliferation	150	14	9.72E-64	4.754073012	4.48E-60	4.98E-61	1.83E-60
GOTERM_BP_FAT	GO:0009719~response to endogenous stimulus	146	13.6	1.22E-61	4.73012657	5.62E-58	5.62E-59	2.30E-58
GOTERM_BP_FAT	GO:0044057~regulation of system process	127	11.9	4.65E-61	5.392872725	2.15E-57	1.95E-58	8.78E-58
GOTERM_BP_FAT	GO:0042592~homeostatic process	198	18.5	8.49E-59	3.459395232	3.92E-55	3.27E-56	1.60E-55
GOTERM_BP_FAT	GO:0044093~positive regulation of molecular function	170	15.9	1.92E-56	3.806503511	8.84E-53	6.80E-54	3.61E-53
GOTERM_BP_FAT	GO:0051240~positive regulation of multicellular organismal process	108	10.1	1.36E-55	5.807762637	6.28E-52	4.48E-53	2.57E-52
GOTERM_BP_FAT	GO:0010647~positive regulation of cell communication	125	11.7	1.79E-55	4.985274131	8.24E-52	5.49E-53	3.37E-52
GOTERM_BP_FAT	GO:0051174~regulation of phosphorus metabolic process	151	14.1	3.07E-54	4.085170038	1.42E-50	8.85E-52	5.79E-51
GOTERM_BP_FAT	GO:0019220~regulation of phosphate metabolic process	151	14.1	3.07E-54	4.085170038	1.42E-50	8.85E-52	5.79E-51
GOTERM_BP_FAT	GO:0009725~response to hormone stimulus	130	12.1	1.19E-53	4.647851217	5.49E-50	3.23E-51	2.25E-50
GOTERM_BP_FAT	GO:0042325~regulation of phosphorylation	147	13.7	1.49E-53	4.139104082	6.87E-50	3.82E-51	2.81E-50

GOTERM_BP_FAT	GO:0032101~regulation of response to external stimulus	86	8.03	4.51E-53	7.097023711	2.08E-49	1.09E-50	8.50E-50
GOTERM_BP_FAT	GO:0043085~positive regulation of catalytic activity	155	14.5	6.05E-53	3.911139297	2.79E-49	1.40E-50	1.14E-49
GOTERM_BP_FAT	GO:0007166~cell surface receptor linked signal transduction	323	30.2	1.75E-52	2.283491923	8.09E-49	3.85E-50	3.31E-49
GOTERM_BP_FAT	GO:0048878~chemical homeostasis	153	14.3	2.17E-52	3.920995999	1.00E-48	4.55E-50	4.10E-49
GOTERM_BP_FAT	GO:0060548~negative regulation of cell death	126	11.8	2.76E-51	4.59243453	1.27E-47	5.53E-49	5.21E-48
GOTERM_BP_FAT	GO:0055082~cellular chemical homeostasis	129	12	8.05E-51	4.454316198	3.71E-47	1.55E-48	1.52E-47
GOTERM_BP_FAT	GO:0006954~inflammatory response	119	11.1	9.49E-51	4.804393046	4.38E-47	1.75E-48	1.79E-47
GOTERM_BP_FAT	GO:0043069~negative regulation of programmed cell death	125	11.7	1.39E-50	4.568677407	6.42E-47	2.47E-48	2.63E-47
GOTERM_BP_FAT	GO:0006873~cellular ion homeostasis	127	11.9	5.01E-50	4.455608749	2.31E-46	8.56E-48	9.46E-47
GOTERM_BP_FAT	GO:0051094~positive regulation of developmental process	109	10.2	8.57E-50	5.144659442	3.95E-46	1.41E-47	1.62E-46
GOTERM_BP_FAT	GO:0019725~cellular homeostasis	142	13.3	1.28E-49	3.998318229	5.90E-46	2.03E-47	2.41E-46
GOTERM_BP_FAT	GO:0007242~intracellular signaling cascade	249	23.2	2.18E-49	2.601265236	1.01E-45	3.35E-47	4.11E-46
GOTERM_BP_FAT	GO:0006928~cell motion	143	13.4	2.86E-49	3.950184287	1.32E-45	4.26E-47	5.41E-46
GOTERM_BP_FAT	GO:0031644~regulation of neurological system process	81	7.56	5.37E-49	6.946539625	2.48E-45	7.75E-47	1.01E-45
GOTERM_BP_FAT	GO:0043066~negative regulation of apoptosis	122	11.4	8.65E-49	4.522009787	3.99E-45	1.21E-46	1.63E-45
GOTERM_BP_FAT	GO:0050801~ion homeostasis	130	12.1	1.42E-47	4.170565762	6.54E-44	1.92E-45	2.68E-44
GOTERM_BP_FAT	GO:0048545~response to steroid hormone stimulus	87	8.12	1.23E-45	5.945562561	5.69E-42	1.62E-43	2.33E-42
GOTERM_BP_FAT	GO:0006952~defense response	159	14.8	2.00E-45	3.392320977	9.23E-42	2.56E-43	3.77E-42
GOTERM_BP_FAT	GO:0019226~transmission of nerve impulse	117	10.9	3.35E-45	4.386243592	1.54E-41	4.17E-43	6.32E-42
GOTERM_BP_FAT	GO:0009967~positive regulation of signal transduction	107	9.99	4.46E-45	4.759229973	2.06E-41	5.41E-43	8.41E-42

GOTERM_BP_FAT	GO:0051969~regulation of transmission of nerve impulse	76	7.1	5.72E-45	6.783771122	2.64E-41	6.76E-43	1.08E-41
GOTERM_BP_FAT	GO:0030182~neuron differentiation	129	12	3.77E-43	3.86447524	1.74E-39	4.34E-41	7.11E-40
GOTERM_BP_FAT	GO:0051241~negative regulation of multicellular organismal process	78	7.28	9.07E-43	6.240590476	4.18E-39	1.02E-40	1.71E-39
GOTERM_BP_FAT	GO:0040012~regulation of locomotion	84	7.84	1.35E-42	5.740543162	6.23E-39	1.48E-40	2.55E-39
GOTERM_BP_FAT	GO:0007268~synaptic transmission	104	9.71	5.02E-42	4.579225226	2.31E-38	5.38E-40	9.47E-39
GOTERM_BP_FAT	GO:0016477~cell migration	98	9.15	2.61E-40	4.658991552	1.20E-36	2.74E-38	4.92E-37
GOTERM_BP_FAT	GO:0045597~positive regulation of cell differentiation	89	8.31	3.55E-40	5.099521811	1.64E-36	3.64E-38	6.69E-37
GOTERM_BP_FAT	GO:0050804~regulation of synaptic transmission	69	6.44	4.16E-40	6.657100474	1.92E-36	4.17E-38	7.85E-37
GOTERM_BP_FAT	GO:0009628~response to abiotic stimulus	113	10.6	1.33E-39	4.029076878	6.15E-36	1.31E-37	2.51E-36
