# Supplementary material for: Molecular Architecture of Spinal Cord Injury Protein Interaction Network
Source: PLoS One. 2015 Aug 4;10(8):e0135024. doi: 10.1371/journal.pone.0135024 (PMC4524728; doi:10.1371/journal.pone.0135024)
Supplement: S5 Table — (PDF) [file pone.0135024.s007.pdf]

Supplementary Table V. Gene Ontology (GO) cellular components over-representation in the entire 1083 accessions performed through DAVID.

Category	Term	Count	%	PValue	Fold Enrichment	Bonferroni	Benjamini	FDR
GOTERM_CC_FAT	GO:0005615~extracellular space	201	18.767507	3.69E-69	3.870619873	1.93E-66	1.93E-66	5.35E-66
GOTERM_CC_FAT	GO:0044421~extracellular region part	234	21.8487395	2.77E-64	3.215286378	1.44E-61	7.22E-62	4.01E-61
GOTERM_CC_FAT	GO:0031226~intrinsic to plasma membrane	246	22.96918768	2.30E-51	2.670753864	1.20E-48	4.01E-49	3.34E-48
GOTERM_CC_FAT	GO:0044459~plasma membrane part	351	32.77310924	1.19E-49	2.101685149	6.22E-47	1.55E-47	1.73E-46
GOTERM_CC_FAT	GO:0005576~extracellular region	331	30.90569561	1.51E-49	2.172235828	7.87E-47	1.57E-47	2.18E-46
GOTERM_CC_FAT	GO:0005887~integral to plasma membrane	237	22.12885154	4.55E-48	2.631521614	2.38E-45	3.96E-46	6.59E-45
GOTERM_CC_FAT	GO:0043005~neuron projection	114	10.6442577	3.17E-44	4.396972824	1.65E-41	2.36E-42	4.59E-41
GOTERM_CC_FAT	GO:0005886~plasma membrane	475	44.35107376	3.04E-40	1.658905553	1.59E-37	1.98E-38	4.40E-37
GOTERM_CC_FAT	GO:0030424~axon	72	6.722689076	6.83E-38	5.973246101	3.57E-35	3.96E-36	9.90E-35
GOTERM_CC_FAT	GO:0000267~cell fraction	191	17.83380019	1.92E-30	2.326376203	1.00E-27	1.00E-28	2.78E-27
GOTERM_CC_FAT	GO:0042995~cell projection	143	13.35200747	2.51E-29	2.706314694	1.31E-26	1.19E-27	3.64E-26
GOTERM_CC_FAT	GO:0009986~cell surface	94	8.776844071	2.27E-28	3.563064185	1.18E-25	9.86E-27	3.28E-25
GOTERM_CC_FAT	GO:0043025~cell soma	62	5.78898226	1.04E-26	4.868077055	5.41E-24	4.16E-25	1.50E-23
GOTERM_CC_FAT	GO:0030425~dendrite	56	5.22875817	2.00E-22	4.531849291	1.04E-19	7.46E-21	2.90E-19
GOTERM_CC_FAT	GO:0031988~membrane-bounded vesicle	112	10.45751634	1.89E-21	2.601026178	9.85E-19	6.57E-20	2.73E-18
GOTERM_CC_FAT	GO:0045202~synapse	84	7.843137255	2.74E-21	3.121231413	1.43E-18	8.95E-20	3.97E-18
GOTERM_CC_FAT	GO:0031982~vesicle	123	11.48459384	8.09E-21	2.421616376	4.22E-18	2.48E-19	1.17E-17
GOTERM_CC_FAT	GO:0009897~external side of plasma membrane	55	5.135387488	1.18E-20	4.267650094	6.14E-18	3.41E-19	1.70E-17
GOTERM_CC_FAT	GO:0060205~cytoplasmic membrane-bounded vesicle lumen	28	2.614379085	7.31E-20	8.394220846	3.82E-17	2.01E-18	1.06E-16
GOTERM_CC_FAT	GO:0045121~membrane raft	49	4.575163399	1.26E-19	4.519965071	6.59E-17	3.29E-18	1.83E-16
GOTERM_CC_FAT	GO:0031983~vesicle lumen	28	2.614379085	3.79E-19	8.029254722	1.98E-16	9.43E-18	5.50E-16
GOTERM_CC_FAT	GO:0016023~cytoplasmic membrane-bounded vesicle	105	9.803921569	4.67E-19	2.518266254	2.44E-16	1.11E-17	6.77E-16
GOTERM_CC_FAT	GO:0005625~soluble fraction	74	6.909430439	8.69E-19	3.118619703	4.53E-16	1.97E-17	1.26E-15
GOTERM_CC_FAT	GO:0030141~secretory granule	54	5.042016807	1.19E-18	3.957275542	6.22E-16	2.59E-17	1.73E-15
GOTERM_CC_FAT	GO:0031410~cytoplasmic vesicle	115	10.73762838	1.27E-18	2.362859228	6.60E-16	2.64E-17	1.83E-15

GOTERM_CC_FAT	GO:0044456~synapse part	64	5.975723623	1.60E-18	3.431783668	8.33E-16	3.20E-17	2.31E-15
GOTERM_CC_FAT	GO:0033267~axon part	29	2.707749767	7.41E-18	7.084011772	3.87E-15	1.43E-16	1.07E-14
GOTERM_CC_FAT	GO:0005626~insoluble fraction	134	12.51167134	2.68E-17	2.10677363	1.40E-14	5.00E-16	3.88E-14
GOTERM_CC_FAT	GO:0044433~cytoplasmic vesicle part	53	4.948646125	3.80E-17	3.738602562	1.98E-14	6.83E-16	5.50E-14
GOTERM_CC_FAT	GO:0031093~platelet alpha granule lumen	25	2.33426704	3.98E-17	8.043242971	2.08E-14	6.93E-16	5.77E-14
GOTERM_CC_FAT	GO:0031091~platelet alpha granule	28	2.614379085	3.13E-16	6.595459236	1.74E-13	5.66E-15	4.77E-13
GOTERM_CC_FAT	GO:0043235~receptor complex	40	3.734827264	4.27E-16	4.548592577	2.32E-13	7.22E-15	6.44E-13
GOTERM_CC_FAT	GO:0005624~membrane fraction	124	11.57796452	1.93E-14	2.021846589	1.01E-11	3.06E-13	2.80E-11
GOTERM_CC_FAT	GO:0045211~postsynaptic membrane	41	3.828197946	2.01E-14	4.006130795	1.05E-11	3.09E-13	2.91E-11
GOTERM_CC_FAT	GO:0044463~cell projection part	55	5.135387488	6.67E-14	3.100429556	3.48E-11	9.95E-13	9.67E-11
GOTERM_CC_FAT	GO:0043679~nerve terminal	21	1.960784314	1.47E-13	7.486737512	7.66E-11	2.13E-12	2.12E-10
GOTERM_CC_FAT	GO:0043195~terminal button	17	1.587301587	1.11E-12	8.969824561	5.78E-10	1.56E-11	1.60E-09
GOTERM_CC_FAT	GO:0043204~perikaryon	17	1.587301587	5.48E-11	7.474853801	2.86E-08	7.53E-10	7.95E-08
GOTERM_CC_FAT	GO:0031012~extracellular matrix	63	5.882352941	1.05E-10	2.408776417	5.46E-08	1.40E-09	1.52E-07
GOTERM_CC_FAT	GO:0008328~ionotropic glutamate receptor complex	12	1.120448179	1.31E-10	11.30650155	6.84E-08	1.71E-09	1.90E-07
GOTERM_CC_FAT	GO:0031594~neuromuscular junction	14	1.307189542	6.51E-10	8.394220846	3.40E-07	8.29E-09	9.44E-07
GOTERM_CC_FAT	GO:0014069~postsynaptic density	24	2.240896359	1.02E-09	4.458902019	5.33E-07	1.27E-08	1.48E-06
GOTERM_CC_FAT	GO:0005829~cytosol	159	14.84593838	2.57E-09	1.576959426	1.34E-06	3.12E-08	3.73E-06
GOTERM_CC_FAT	GO:0005901~caveola	20	1.867413632	2.95E-09	5.073430182	1.54E-06	3.50E-08	4.28E-06
GOTERM_CC_FAT	GO:0042734~presynaptic membrane	15	1.400560224	4.92E-09	6.822888865	2.57E-06	5.70E-08	7.12E-06
GOTERM_CC_FAT	GO:0005578~proteinaceous extracellular matrix	53	4.948646125	1.11E-07	2.184745872	5.80E-05	1.26E-06	1.61E-04
GOTERM_CC_FAT	GO:0031225~anchored to membrane	40	3.734827264	4.62E-07	2.398348813	2.41E-04	5.13E-06	6.70E-04
GOTERM_CC_FAT	GO:0032838~cell projection cytoplasm	7	0.653594771	1.22E-06	13.19091847	6.35E-04	1.32E-05	0.00176
