# Supplementary material for: Molecular Architecture of Spinal Cord Injury Protein Interaction Network
Source: PLoS One. 2015 Aug 4;10(8):e0135024. doi: 10.1371/journal.pone.0135024 (PMC4524728; doi:10.1371/journal.pone.0135024)
Supplement: S6 Table — (PDF) [file pone.0135024.s008.pdf]

Supplementary Table VI. KEGG pathway enrichment on the entire 1083 accessions performed through DAVID.								
Category	Term	Count	%	PValue	Fold Enrichment	Bonferroni	Benjamini	FDR
KEGG_PATHWAY	hsa05200:Pathways in cancer	130	12	5.80E-31	2.73831025	9.80E-29	9.80E-29	7.07E-28
KEGG_PATHWAY	hsa04080:Neuroactive ligand-receptor interaction	104	9.7	1.39E-25	2.806768	2.35E-23	1.17E-23	1.69E-22
KEGG_PATHWAY	hsa04610:Complement and coagulation cascades	45	4.2	3.66E-21	4.5058483	6.19E-19	2.06E-19	4.46E-18
KEGG_PATHWAY	hsa04210:Apoptosis	50	4.7	4.22E-20	3.97067091	7.12E-18	1.78E-18	5.14E-17
KEGG_PATHWAY	hsa04620:Toll-like receptor signaling pathway	54	5	9.15E-20	3.69390336	1.55E-17	3.09E-18	1.12E-16
KEGG_PATHWAY	hsa04060:Cytokine-cytokine receptor interaction	95	8.9	3.03E-19	2.50515993	5.12E-17	8.53E-18	3.69E-16
KEGG_PATHWAY	hsa04020:Calcium signaling pathway	73	6.8	1.57E-18	2.86565125	2.65E-16	3.78E-17	1.91E-15
KEGG_PATHWAY	hsa04722:Neurotrophin signaling pathway	59	5.5	2.06E-18	3.28733126	3.48E-16	4.35E-17	2.51E-15
KEGG_PATHWAY	hsa05215:Prostate cancer	48	4.5	8.45E-18	3.72618466	1.43E-15	1.59E-16	1.03E-14
KEGG_PATHWAY	hsa05212:Pancreatic cancer	42	3.9	3.05E-17	4.03023098	5.16E-15	5.16E-16	3.72E-14
KEGG_PATHWAY	hsa05014:Amyotrophic lateral sclerosis (ALS)	35	3.3	9.81E-17	4.56252564	1.88E-14	1.67E-15	1.33E-13
KEGG_PATHWAY	hsa04010:MAPK signaling pathway	89	8.3	2.79E-15	2.30298913	4.69E-13	3.91E-14	3.39E-12
KEGG_PATHWAY	hsa04370:VEGF signaling pathway	40	3.7	1.29E-14	3.68478261	2.18E-12	1.67E-13	1.57E-11
KEGG_PATHWAY	hsa05214:Glioma	36	3.4	2.14E-14	3.94798137	3.62E-12	2.59E-13	2.61E-11
KEGG_PATHWAY	hsa05218:Melanoma	38	3.5	5.74E-14	3.6977572	9.70E-12	6.47E-13	7.00E-11
KEGG_PATHWAY	hsa04510:Focal adhesion	71	6.6	1.16E-13	2.44048102	1.97E-11	1.23E-12	1.42E-10
KEGG_PATHWAY	hsa05223:Non-small cell lung cancer	32	3	2.03E-13	4.0942029	3.44E-11	2.02E-12	2.48E-10
KEGG_PATHWAY	hsa04360:Axon guidance	53	4.9	2.85E-13	2.838568	4.82E-11	2.68E-12	3.48E-10
KEGG_PATHWAY	hsa05020:Prion diseases	25	2.3	4.24E-13	4.93497671	7.16E-11	3.77E-12	5.17E-10
KEGG_PATHWAY	hsa04920:Adipocytokine signaling pathway	35	3.3	1.69E-12	3.60916207	2.85E-10	1.43E-11	2.06E-09
KEGG_PATHWAY	hsa04012:ErbB signaling pathway	40	3.7	5.77E-12	3.17653673	9.75E-10	4.64E-11	7.03E-09
KEGG_PATHWAY	hsa05222:Small cell lung cancer	39	3.6	7.70E-12	3.20773486	1.30E-09	5.91E-11	9.38E-09
KEGG_PATHWAY	hsa05210:Colorectal cancer	39	3.6	7.70E-12	3.20773486	1.30E-09	5.91E-11	9.38E-09
KEGG_PATHWAY	hsa04912:GnRH signaling pathway	42	3.9	2.50E-11	2.96098602	4.23E-09	1.84E-10	3.05E-08
KEGG_PATHWAY	hsa05219:Bladder cancer	25	2.3	1.30E-10	4.11248059	2.21E-08	9.19E-10	1.59E-07

KEGG_PATHWAY	hsa04621:NOD-like receptor signaling pathway	31	2.9	1.64E-10	3.4544837	2.77E-08	1.11E-09	2.00E-07
KEGG_PATHWAY	hsa05221:Acute myeloid leukemia	29	2.7	7.25E-10	3.4544837	1.23E-07	4.72E-09	8.84E-07
KEGG_PATHWAY	hsa04660:T cell receptor signaling pathway	42	3.9	9.17E-10	2.68682065	1.55E-07	5.74E-09	1.12E-06
KEGG_PATHWAY	hsa04540:Gap junction	36	3.4	5.42E-09	2.7946385	9.16E-07	3.27E-08	6.61E-06
KEGG_PATHWAY	hsa05213:Endometrial cancer	26	2.4	6.72E-09	3.4544837	1.14E-06	3.92E-08	8.19E-06
KEGG_PATHWAY	hsa04664:Fc epsilon RI signaling pathway	33	3.1	7.27E-09	2.92302467	1.23E-06	4.09E-08	8.86E-06
KEGG_PATHWAY	hsa04640:Hematopoietic cell lineage	35	3.3	7.67E-09	2.81178905	1.30E-06	4.18E-08	9.34E-06
KEGG_PATHWAY	hsa04062:Chemokine signaling pathway	56	5.2	6.16E-08	2.06899558	1.04E-05	3.25E-07	7.51E-05
KEGG_PATHWAY	hsa05220:Chronic myeloid leukemia	30	2.8	1.75E-07	2.76358696	2.96E-05	8.97E-07	2.13E-04
KEGG_PATHWAY	hsa04720:Long-term potentiation	28	2.6	2.47E-07	2.84486893	4.18E-05	1.23E-06	3.02E-04
KEGG_PATHWAY	hsa04910:Insulin signaling pathway	43	4	4.45E-07	2.20063406	7.52E-05	2.15E-06	5.42E-04
KEGG_PATHWAY	hsa04914:Progesterone-mediated oocyte maturation	31	2.9	1.45E-06	2.49044173	2.44E-04	6.78E-06	0.001761342
KEGG_PATHWAY	hsa04630:Jak-STAT signaling pathway	46	4.3	1.52E-06	2.05040323	2.56E-04	6.93E-06	0.001849379
KEGG_PATHWAY	hsa05216:Thyroid cancer	16	1.5	2.35E-06	3.81184408	3.96E-04	1.04E-05	0.002858424
KEGG_PATHWAY	hsa04662:B cell receptor signaling pathway	28	2.6	2.43E-06	2.57934783	4.10E-04	1.05E-05	0.002956013
KEGG_PATHWAY	hsa05010:Alzheimer's disease	47	4.4	2.76E-06	1.99215624	4.67E-04	1.17E-05	0.003365885
KEGG_PATHWAY	hsa05120:Epithelial cell signaling in Helicobacter pylori infection	26	2.4	3.69E-06	2.641664	6.24E-04	1.52E-05	0.004503097
KEGG_PATHWAY	hsa05211:Renal cell carcinoma	26	2.4	6.76E-06	2.56618789	0.001141046	2.72E-05	0.008233181
KEGG_PATHWAY	hsa04622:RIG-I-like receptor signaling pathway	26	2.4	9.03E-06	2.5300444	0.00152452	3.55E-05	0.011002081
KEGG_PATHWAY	hsa04930:Type II diabetes mellitus	20	1.9	1.12E-05	2.93998612	0.00189161	4.30E-05	0.013653611
KEGG_PATHWAY	hsa04670:Leukocyte transendothelial migration	36	3.4	1.34E-05	2.10782056	0.002267543	5.04E-05	0.016369947
KEGG_PATHWAY	hsa04730:Long-term depression	25	2.3	1.73E-05	2.50324905	0.002917261	6.35E-05	0.021066778
KEGG_PATHWAY	hsa04270:Vascular smooth muscle contraction	34	3.2	2.75E-05	2.0973651	0.004643059	9.90E-05	0.033556438
KEGG_PATHWAY	hsa04115:p53 signaling pathway	23	2.1	1.34E-04	2.33685662	0.022374111	4.71E-04	0.16305354
KEGG_PATHWAY	hsa04150:mTOR signaling pathway	19	1.8	2.08E-04	2.52443039	0.03460841	7.19E-04	0.25368242
KEGG_PATHWAY	hsa04916:Melanogenesis	29	2.7	2.40E-04	2.02383893	0.039747462	8.11E-04	0.29206955
KEGG_PATHWAY	hsa04810:Regulation of actin cytoskeleton	51	4.8	2.89E-04	1.63887133	0.047633744	9.57E-04	0.351349974
KEGG_PATHWAY	hsa04350:TGF-beta signaling pathway	25	2.3	9.71E-04	1.98533546	0.151439853	0.003153	1.177270698

KEGG_PATHWAY	hsa04940:Type I diabetes mellitus	15	1.4	0.00159	2.46748835	0.235620561	0.005057	1.919051188
KEGG_PATHWAY	hsa05410:Hypertrophic cardiomyopathy (HCM)	24	2.2	0.00163	1.95076726	0.241373894	0.005103	1.972476963
KEGG_PATHWAY	hsa04650:Natural killer cell mediated cytotoxicity	33	3.1	0.00196	1.71425507	0.281988785	0.006005	2.3606917
KEGG_PATHWAY	hsa04114:Oocyte meiosis	28	2.6	0.00318	1.75864625	0.416083051	0.009561	3.805538413
KEGG_PATHWAY	hsa05330:Allograft rejection	13	1.2	0.00338	2.49490489	0.436060831	0.009999	4.046736038
KEGG_PATHWAY	hsa04110:Cell cycle	30	2.8	0.00547	1.65815217	0.604157247	0.015851	6.464867965
KEGG_PATHWAY	hsa05322:Systemic lupus erythematosus	25	2.3	0.00621	1.74468874	0.650912312	0.01768	7.308895218
KEGG_PATHWAY	hsa04514:Cell adhesion molecules (CAMs)	31	2.9	0.00652	1.62256052	0.668827329	0.01825	7.66039104
KEGG_PATHWAY	hsa04672:Intestinal immune network for IgA production	15	1.4	0.00767	2.11499002	0.727957273	0.021115	8.960883794
KEGG_PATHWAY	hsa04623:Cytosolic DNA-sensing pathway	16	1.5	0.00933	2.00988142	0.79495301	0.025233	10.79826404
KEGG_PATHWAY	hsa05416:Viral myocarditis	19	1.8	0.0104	1.8488786	0.828991526	0.027643	11.95839518
KEGG_PATHWAY	hsa04310:Wnt signaling pathway	33	3.1	0.01451	1.50990678	0.915424077	0.03786	16.3170777
KEGG_PATHWAY	hsa05414:Dilated cardiomyopathy	22	2.1	0.01985	1.65214438	0.96624702	0.050798	21.68100846
KEGG_PATHWAY	hsa04520:Adherens junction	19	1.8	0.02366	1.70481014	0.982527619	0.059478	25.31306049
KEGG_PATHWAY	hsa04960:Aldosterone-regulated sodium reabsorption	12	1.1	0.02733	2.0221368	0.990754233	0.067517	28.66362881
KEGG_PATHWAY	hsa04666:Fc gamma R-mediated phagocytosis	22	2.1	0.02782	1.5999714	0.991508213	0.067725	29.09991497
KEGG_PATHWAY	hsa04512:ECM-receptor interaction	20	1.9	0.02833	1.64499224	0.992229064	0.067976	29.55204132
KEGG_PATHWAY	hsa05130:Pathogenic Escherichia coli infection	15	1.4	0.02907	1.81814931	0.993160245	0.068738	30.19752904
KEGG_PATHWAY	hsa05332:Graft-versus-host disease	11	1	0.0461	1.94868311	0.999656409	0.106259	43.74104914
KEGG_PATHWAY	hsa05217:Basal cell carcinoma	14	1.3	0.04611	1.75864625	0.999656947	0.104883	43.74740913
KEGG_PATHWAY	hsa04340:Hedgehog signaling pathway	14	1.3	0.05245	1.72724185	0.99988879	0.11725	48.13649827
