# Supplementary material for: Molecular Architecture of Spinal Cord Injury Protein Interaction Network
Source: PLoS One. 2015 Aug 4;10(8):e0135024. doi: 10.1371/journal.pone.0135024 (PMC4524728; doi:10.1371/journal.pone.0135024)
Supplement: S7 Table — (PDF) [file pone.0135024.s009.pdf]

Supplementary Table VII. KEGG pathway enrichment on rich-club accessions performed through DAVID.

Category	Term	Count	%	PValue	Fold Enrichment	Bonferroni	Benjamini	FDR
KEGG_PATHWAY	hsa05200:Pathways in cancer	83	20	1.09E-28	3.806961683	1.43E-26	1.43E-26	1.27E-25
KEGG_PATHWAY	hsa04060:Cytokine-cytokine receptor interaction	65	16	1.18E-21	3.732384028	1.54E-19	7.71E-20	1.37E-18
KEGG_PATHWAY	hsa04620:Toll-like receptor signaling pathway	38	9.1	3.75E-19	5.660261292	4.91E-17	1.64E-17	4.37E-16
KEGG_PATHWAY	hsa04080:Neuroactive ligand-receptor interaction	59	14	5.89E-18	3.467259153	7.71E-16	1.93E-16	6.86E-15
KEGG_PATHWAY	hsa04210:Apoptosis	33	7.9	9.33E-17	5.706488472	1.45E-14	2.44E-15	1.33E-13
KEGG_PATHWAY	hsa04062:Chemokine signaling pathway	46	11	5.19E-15	3.700756257	6.84E-13	8.55E-14	6.08E-12
KEGG_PATHWAY	hsa04722:Neurotrophin signaling pathway	37	8.9	6.61E-15	4.489048483	8.73E-13	9.69E-14	7.77E-12
KEGG_PATHWAY	hsa04510:Focal adhesion	43	10	7.59E-12	3.218449174	9.95E-10	8.29E-11	8.85E-09
KEGG_PATHWAY	hsa04020:Calcium signaling pathway	39	9.3	2.93E-11	3.333697552	3.84E-09	2.95E-10	3.42E-08
KEGG_PATHWAY	hsa04610:Complement and coagulation cascades	23	5.5	2.00E-10	5.014792899	2.62E-08	1.38E-09	2.33E-07
KEGG_PATHWAY	hsa04630:Jak-STAT signaling pathway	34	8.1	9.52E-10	3.300057263	1.25E-07	5.94E-09	1.11E-06
KEGG_PATHWAY	hsa04621:NOD-like receptor signaling pathway	21	5	1.09E-09	5.095676656	1.42E-07	6.46E-09	1.27E-06
KEGG_PATHWAY	hsa04660:T cell receptor signaling pathway	27	6.5	4.08E-09	3.761094675	5.35E-07	2.32E-08	4.76E-06
KEGG_PATHWAY	hsa04920:Adipocytokine signaling pathway	21	5	5.02E-09	4.715402279	6.58E-07	2.74E-08	5.86E-06
KEGG_PATHWAY	hsa04540:Gap junction	22	5.3	1.94E-07	3.718835184	2.55E-05	8.78E-07	2.27E-04
KEGG_PATHWAY	hsa04010:MAPK signaling pathway	42	10	2.03E-07	2.366531481	2.65E-05	8.85E-07	2.36E-04
KEGG_PATHWAY	hsa04664:Fc epsilon RI signaling pathway	20	4.8	4.43E-07	3.857533	5.81E-05	1.87E-06	5.17E-04
KEGG_PATHWAY	hsa04370:VEGF signaling pathway	19	4.5	1.14E-06	3.811242604	1.49E-04	4.51E-06	0.0013244
KEGG_PATHWAY	hsa05020:Prion diseases	13	3.1	1.23E-06	5.587912088	1.61E-04	4.74E-06	0.0014336
KEGG_PATHWAY	hsa04670:Leukocyte transendothelial migration	24	5.7	1.94E-06	3.059873634	2.54E-04	7.25E-06	0.0022596
KEGG_PATHWAY	hsa04914:Progesterone-mediated oocyte maturation	20	4.8	2.21E-06	3.498692721	2.89E-04	8.03E-06	0.0025717
KEGG_PATHWAY	hsa04622:RIG-I-like receptor signaling pathway	18	4.3	2.35E-06	3.814067839	3.08E-04	8.32E-06	0.0027395
KEGG_PATHWAY	hsa05211:Renal cell carcinoma	17	4.1	8.92E-06	3.653634827	0.0011682	3.08E-05	0.0104043
KEGG_PATHWAY	hsa04910:Insulin signaling pathway	24	5.7	2.04E-05	2.674556213	0.0026752	6.87E-05	0.0238429

KEGG_PATHWAY	hsa04810:Regulation of actin cytoskeleton	32	7.7	2.54E-05	2.239163341	0.0033265	8.33E-05	0.0296571
KEGG_PATHWAY	hsa04623:Cytosolic DNA-sensing pathway	14	3.3	4.29E-05	3.829478214	0.0056092	1.37E-04	0.05006
KEGG_PATHWAY	hsa05216:Thyroid cancer	10	2.4	6.78E-05	5.187716792	0.0088389	2.11E-04	0.079001
KEGG_PATHWAY	hsa04115:p53 signaling pathway	15	3.6	1.11E-04	3.318612948	0.0144778	3.39E-04	0.1297362
KEGG_PATHWAY	hsa04650:Natural killer cell mediated cytotoxicity	22	5.3	1.47E-04	2.488543845	0.0190732	4.38E-04	0.1712794
KEGG_PATHWAY	hsa04930:Type II diabetes mellitus	12	2.9	1.84E-04	3.841117966	0.0238267	5.36E-04	0.2144381
KEGG_PATHWAY	hsa04912:GnRH signaling pathway	18	4.3	1.99E-04	2.76325323	0.0256834	5.65E-04	0.2313482
KEGG_PATHWAY	hsa04662:B cell receptor signaling pathway	15	3.6	3.31E-04	3.00887574	0.0424667	9.23E-04	0.3855468
KEGG_PATHWAY	hsa04110:Cell cycle	20	4.8	4.99E-04	2.407100592	0.0632329	0.0013599	0.5797848
KEGG_PATHWAY	hsa04940:Type I diabetes mellitus	10	2.4	0.0014	3.581994928	0.1676889	0.0037389	1.6206431
KEGG_PATHWAY	hsa04720:Long-term potentiation	13	3.1	0.00147	2.876131222	0.1748946	0.0038375	1.6967613
KEGG_PATHWAY	hsa04150:mTOR signaling pathway	11	2.6	0.00185	3.182464725	0.215084	0.0047373	2.1327565
KEGG_PATHWAY	hsa04916:Melanogenesis	16	3.8	0.00198	2.431414739	0.228992	0.0049886	2.2883846
KEGG_PATHWAY	hsa05330:Allograft rejection	9	2.2	0.00201	3.761094675	0.2315336	0.0049567	2.3171006
KEGG_PATHWAY	hsa05416:Viral myocarditis	13	3.1	0.00215	2.75460455	0.2461126	0.005218	2.4835126
KEGG_PATHWAY	hsa04144:Endocytosis	24	5.7	0.00217	1.962310265	0.2473718	0.0051537	2.4980222
KEGG_PATHWAY	hsa04514:Cell adhesion molecules (CAMs)	19	4.5	0.00249	2.165478752	0.2787889	0.0058191	2.8674079
KEGG_PATHWAY	hsa04672:Intestinal immune network for IgA production	10	2.4	0.00428	3.070281367	0.4297956	0.009807	4.8777111
KEGG_PATHWAY	hsa04520:Adherens junction	13	3.1	0.00432	2.53996004	0.4329024	0.0097321	4.9239622
KEGG_PATHWAY	hsa04114:Oocyte meiosis	16	3.8	0.00557	2.188273265	0.5189714	0.0123273	6.306935
KEGG_PATHWAY	hsa05010:Alzheimer's disease	20	4.8	0.01079	1.845936037	0.7587075	0.0234172	11.887971
KEGG_PATHWAY	hsa04350:TGF-beta signaling pathway	13	3.1	0.0115	2.24801061	0.7802668	0.0245356	12.619045
KEGG_PATHWAY	hsa05130:Pathogenic Escherichia coli infection	10	2.4	0.01175	2.639364684	0.7875014	0.0246715	12.879072
KEGG_PATHWAY	hsa04730:Long-term depression	11	2.6	0.0146	2.398379213	0.854311	0.0301132	15.75778
KEGG_PATHWAY	hsa04666:Fc gamma R-mediated phagocytosis	13	3.1	0.02207	2.058704453	0.9462656	0.0446551	22.915083
KEGG_PATHWAY	hsa04614:Renin-angiotensin system	5	1.2	0.02264	4.424817264	0.9501988	0.0451008	23.43492
KEGG_PATHWAY	hsa05332:Graft-versus-host disease	7	1.7	0.04133	2.7002731	0.9960294	0.0803576	38.869988
KEGG_PATHWAY	hsa04360:Axon guidance	15	3.6	0.04512	1.74934636	0.9976375	0.086315	41.631052
KEGG_PATHWAY	hsa04512:ECM-receptor interaction	11	2.6	0.04956	1.97009721	0.9987169	0.0932779	44.7183

KEGG_PATHWAY	hsa04960:Aldosterone-regulated sodium reabsorption	7	1.7	0.05104	2.568552461	0.9989536	0.0946688	45.712379
KEGG_PATHWAY	hsa04270:Vascular smooth muscle contraction	13	3.1	0.06573	1.746222527	0.9998646	0.1194835	54.747302
KEGG_PATHWAY	hsa05414:Dilated cardiomyopathy	11	2.6	0.08169	1.79878441	0.9999858	0.1454964	62.982081
