# Supplementary material for: Molecular Architecture of Spinal Cord Injury Protein Interaction Network
Source: PLoS One. 2015 Aug 4;10(8):e0135024. doi: 10.1371/journal.pone.0135024 (PMC4524728; doi:10.1371/journal.pone.0135024)
Supplement: S8 Table — (PDF) [file pone.0135024.s010.pdf]

Supplementary Table VIII. Distribution of core rich-club proteins across biological pathways using PantherDB.

Pathway	Percent genes
**Pathways of Cell Death**	
Apoptosis signaling pathway (P00006)	5.3%
p53 pathway (P00059)	4.5%
Ras Pathway (P04393)	2.4%
FAS signaling pathway (P00020)	1.7%
JAK/STAT signaling pathway (P00038)	1.4%
p38 MAPK pathway (P05918)	1.2%
**Hormone Signaling Pathways**	
CCKR signaling map (P06959)	8.4%
Gonadotropin releasing hormone receptor pathway (P06664)	6.5%
**Growth Factor Signaling**	
Angiogenesis (P00005)	5.3%
EGF receptor signaling pathway (P00018)	4.2%
PDGF signaling pathway (P00047)	4.1%
FGF signaling pathway (P00021)	3.2%
VEGF signaling pathway (P00056)	2.6%
TGF-beta signaling pathway (P00052)	2.3%
Insulin/IGF pathway	1.5%
**Immune and Inflammatory Pathways**	
Inflammation mediated by chemokine and cytokine signaling pathway (P00031)	7.8%
Interleukin signaling pathway (P00036)	4.7%
Blood coagulation (P00011)	2.9%
Toll receptor signaling pathway (P00054)	2.7%
T cell activation (P00053)	2.7%
B cell activation (P00010)	2.6%
Interferon-gamma signaling pathway (P00035)	1.5%
Plasminogen activating cascade (P00050)	1.5%
Oxidative stress response (P00046)	1.2%
**Cell Signaling Pathways**	
Heterotrimeric G-protein signaling pathway (P00027)	6.0%
Integrin signalling pathway (P00034)	3.6%
PI3 kinase pathway (P00048)	2.9%
Axon Guidance	2.3%
Endothelin signaling pathway (P00019)	2.3%
Wnt signaling pathway (P00057)	1.4%
