# Supplementary material for: Molecular Architecture of Spinal Cord Injury Protein Interaction Network
Source: PLoS One. 2015 Aug 4;10(8):e0135024. doi: 10.1371/journal.pone.0135024 (PMC4524728; doi:10.1371/journal.pone.0135024)
Supplement: S9 Table — (PDF) [file pone.0135024.s011.pdf]

Supplementary Table IX. Weighted degrees of the different rich-club modules within the module interaction network.

Module	Weighted Degree
Developmental Processes	2670
Immune System Process	2407
Cell Communication	1651
Response To Stress	907
G-Protein Coupled Receptor Signaling Pathway	866
Inflammatory Response	848
Cell Surface Receptor Linked Signal Transduction	693
Platelet Activation	676
Apoptotic Processes	655
Adenylate Cyclase-Modulating G-Protein Coupled Receptor Signaling Pathway	512
Signal Transduction	474
Non-Enriched Module	431
Angiogenesis	384
Cell-Cell Signaling	250
Toll-Like Receptor Signaling Pathway	244
Response To Chemical	240
Regulation Of Apoptotic Signaling Pathway	229
Response To Hypoxia	216
Response To Growth Factor	199
Cellular Response To Organic Substance	157
Carbohydrate Derivative Metabolic Process	152
Extracellular Matrix Organization	152
Synaptic Transmission	134
Regulation Of Nervous System Development	110
Steroid Hormone Mediated Signaling Pathway	87
Activation Of Protein Kinase Activity	66
